# Nanovesicles loaded with a TGF-β receptor 1 inhibitor overcome immune resistance to potentiate cancer immunotherapy

**DOI:** 10.1038/s41467-023-39035-x

**Published:** 2023-06-16

**Authors:** Mengxue Zhou, Jiaxin Wang, Jiaxing Pan, Hui Wang, Lujia Huang, Bo Hou, Yi Lai, Fengyang Wang, Qingxiang Guan, Feng Wang, Zhiai Xu, Haijun Yu

**Affiliations:** 1 Center of Pharmaceutics, Shanghai Institute of Materia Medica, Chinese Academy of Sciences, Shanghai, 201203 China; 2 College of Chemistry and Chemical Engineering, Inner Mongolia University, Huhhot, 010021 China; 3 Shanghai Tenth People’s Hospital, Tongji University School of Medicine, Shanghai, 200072 China; 4 School of Pharmacy, Jilin University, Changchun, 130021 China; 5 Department of Gastroenterology, Huadong Hospital, Shanghai Medical College, Fudan University, Shanghai, 200040 China; 6 School of Chemistry and Molecular Engineering, East China Normal University, Shanghai, 200241 China

**Keywords:** Nanotechnology in cancer, Immunization, Cancer microenvironment

## Abstract

The immune-excluded tumors (IETs) show limited response to current immunotherapy due to intrinsic and adaptive immune resistance. In this study, it is identified that inhibition of transforming growth factor-β (TGF-β) receptor 1 can relieve tumor fibrosis, thus facilitating the recruitment of tumor-infiltrating T lymphocytes. Subsequently, a nanovesicle is constructed for tumor-specific co-delivery of a TGF-β inhibitor (LY2157299, LY) and the photosensitizer pyropheophorbide a (PPa). The LY-loaded nanovesicles suppress tumor fibrosis to promote intratumoral infiltration of T lymphocytes. Furthermore, PPa chelated with gadolinium ion is capable of fluorescence, photoacoustic and magnetic resonance triple-modal imaging-guided photodynamic therapy, to induce immunogenic death of tumor cells and elicit antitumor immunity in preclinical cancer models in female mice. These nanovesicles are further armored with a lipophilic prodrug of the bromodomain-containing protein 4 inhibitor (i.e., JQ1) to abolish programmed death ligand 1 expression of tumor cells and overcome adaptive immune resistance. This study may pave the way for nanomedicine-based immunotherapy of the IETs.

## Abstract (web summary)

Targeting the TGF-β signaling pathway has been exploited to relieve immunosuppression in the tumor microenvironment. Here the authors describe the design of a nanoplatform integrating the TGF-β receptor 1 inhibitor LY2157299 and the ROS-responsive JQ1 pro-drug, promoting anti-tumor immune responses in preclinical cancer models.

## Introduction

Immunotherapy that specifically attacks tumor cells is the preferred method for tumor regression^1,2^. Over the past decade, progress has been made in designing various approaches to activate the immune system and eradicate tumor cells^3^. However, the immunotherapy of the immune-excluded tumors (IETs) is severely impaired by the low immunogenicity of tumors, insufficient intratumoral infiltration of T lymphocytes, and the immunosuppressive tumor microenvironment (ITM)^4–6^. The transforming growth factor-β (TGF-β), a pleiotropic cytokine plays diverse roles in cancer immunity, in particular, it has an immunosuppressive effect that is overexpressed in advanced tumors and dramatically attenuates the antitumor performance of cytotoxic T lymphocytes (CTLs)^7,8^. For example, TGF-β1, a member of the TGF-β superfamily, can promote the activation of cancer-associated fibroblasts (CAFs) to cause uncontrolled deposition of extracellular matrix (ECM) and induce tumor fibrosis by the TGF-β/Smad signaling pathway^9^. This results in a “physical barrier” to hinder the intratumor infiltration of the CTLs, therefore suppressing the protective immune responses^10^, which is one of the crucial mechanisms accounting for intrinsic immune resistance. Targeting the TGF-β signaling pathway to inactivate CAFs and breach the “physical barrier” might be a promising strategy to overcome the exclusion of CTLs and improve the therapeutic efficacy of cancer immunotherapy^11^.

Apart from the intrinsic immune resistance caused by hyperactivation of TGF-β signaling, the tumor-infiltrating CTLs secrete interferon-gamma (IFN-γ) to activate the JAK/STAT (Janus kinase/signal transducer and activator of transcription) signaling pathway, then elevate the expression of the programmed death ligand 1 (PD-L1) and indoleamine 2,3-dioxygenase 1 (IDO-1), which in turn inactivates CTLs and leads to inducible immune evasion^12–14^. This negative regulatory mechanism accounts for the failure of anti-tumor immunotherapy^15^. Therefore, it remains a formidable challenge to override the intrinsic immune resistance induced by the TGF-β signaling pathway and avoid the inducible immune evasion caused by IFN-γ, hence enforcing the immunotherapy of IETs.

**Fig. 1:**
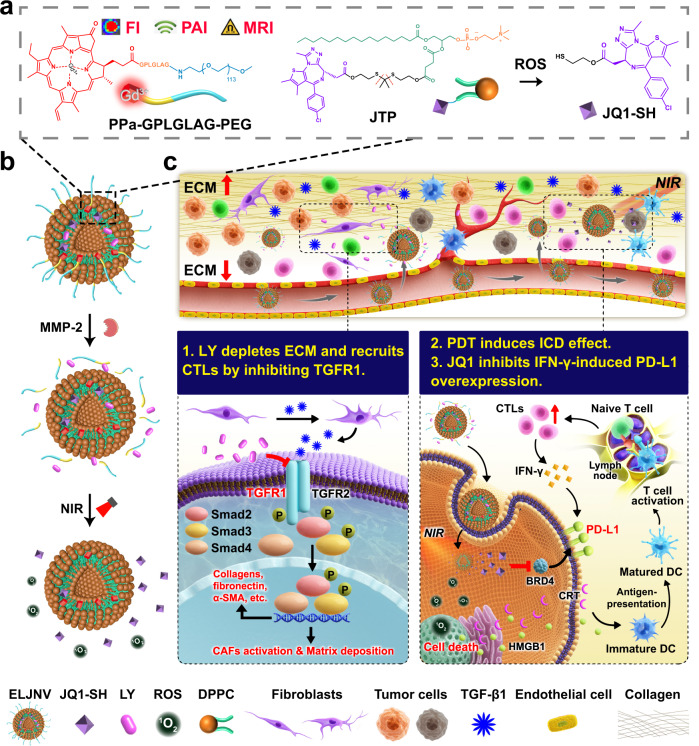
Schematic of the nanovesicles to avoid the immune resistance of immune-excluded tumors (IETs). **a** The chemical structure of the nanovesicles integrating a phospholipid prodrug of JQ1, photosensitizer pyropheophorbide a (PPa), and transforming growth factor β receptor 1 (TGFR1) inhibitor LY2157299 (LY). **b** Schematic illustration of the cascade drug release of the nanovesicles. Matrix metallopeptidase-2 (MMP-2) cleaved the poly(ethylene glycol) (PEG) corona and induced the LY release. PPa generated singlet oxygen upon near-infrared (NIR) laser irradiation to release JQ1-SH. **c** Diagram illustrating the mechanism of the nanovesicles to overcome immune resistance. The nanovesicles (ELJNV) breach the physical barrier and enhance the tumor-specific immune response upon the 671 nm laser irradiation to overcome the intrinsic immune resistance and simultaneously suppress the interferon-γ (IFN-γ)-induced inducible immune resistance in vivo. FI fluorescence imaging, PAI photoacoustic imaging, MRI nuclear magnetic resonance imaging, ROS reactive oxygen species, ECM extracellular matrix, CTLs cytotoxic T lymphocytes, α-SMA α-smooth muscle actin, PDT photodynamic therapy, ICD immunogenic cell death, PD-L1 programmed cell death 1, BRD4 bromodomain-containing protein 4, HMGB1 high mobility group box protein 1, CRT calreticulin, DC dendritic cell, CAFs cancer-associated fibroblasts, DPPC 1,2-dipalmitoyl-sn-glycero-3-phosphocholine, JTP JQ1-thioketal (TK)-pPC, TGF-β1 transforming growth factor β1, ^1^O_2_ singlet oxygen, Gd^3+^ gadolinium ion.

To this end, this study proposes a versatile nanoplatform for boosting cancer immunotherapy by addressing both intrinsic and inducible immune resistance mechanisms. First, it is validated that LY2157299 (LY), an inhibitor of TGF-β receptor 1 (TGFR1) suppresses TGF-β1-induced CAF activation and tumor fibrosis by inhibiting the TGF-β/Smad signaling pathway, thereby paving the way to promote intratumoral infiltration of T lymphocytes. Subsequently, tumor enzyme-activatable nanovesicles integrating LY and gadolinium (Gd^3+^)-chelated pyropheophorbide a (PPa), are engineered. The nanovesicles undergo multi-model molecular imaging, including photoacoustic and magnetic resonance and fluorescence imaging, which guide precise near-infrared (NIR) laser irradiation at the tumor site to induce reactive oxygen species (ROS) generation and trigger immunogenic cell death (ICD) of the tumor cells, thus improving the immunogenicity of the tumor cells and recruiting tumor-infiltrating CTLs. The nanovesicles are further armored with a ROS-responsive JQ1 prodrug to achieve spatiotemporally tunable JQ1 release and inhibit IFN-γ-inducible PD-L1 overexpression, which relieves adaptive immune resistance. This study demonstrates that prodrug nanovesicles promote the immunotherapy of IETs by overcoming intrinsic and inducible immune resistance, and might open an avenue for the immunotherapy of IETs (Fig. 1).

## Results

### LY inhibited tumor fibrosis and recruited tumor-infiltrating T cells by downregulating the TGF-β signaling pathway

**Fig. 2:**
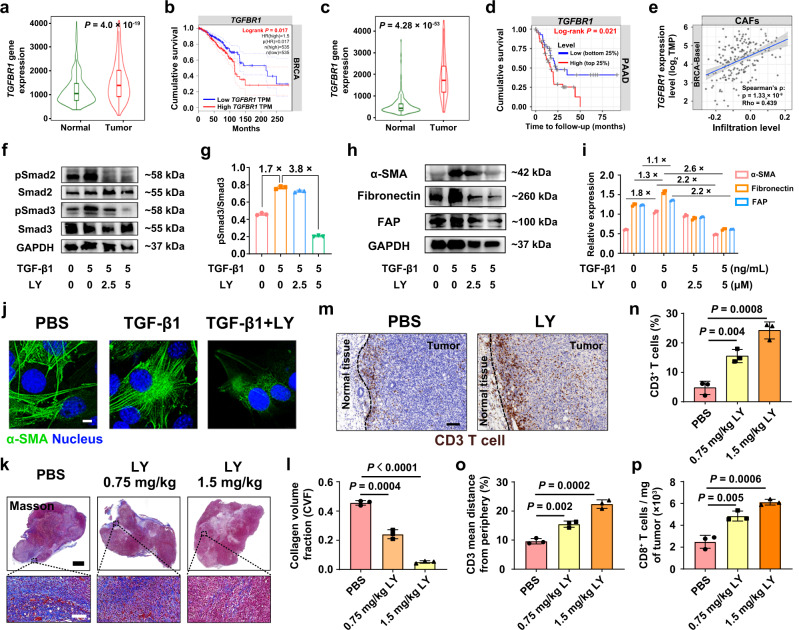
LY promoted the tumor infiltration of T cells by blocking the TGF-β signaling pathway. **a**, **c** We compared the *TGFBR1* gene expression of **a** breast invasive carcinoma tissues (from 1097 patients) with normal breast tissues (from 403 patients) and **c** pancreatic adenocarcinoma tissues (from 177 patients) with normal pancreatic tissues (from 252 patients) using Mann–Whitney test. Plots were downloaded from the online database TNMplot. **b**, **d** Cumulative survival analysis of **b** breast cancer (BRCA, *n* = 535 patients) and **d** pancreatic adenocarcinoma patients (179 patients with 93 dying) with low or high *TGFBR1* expression using log-rank Mantel–Cox test. Plots were downloaded from the online database **b** GEPIA and **d** Timer. **e** Correlation between *TGFBR1* expression and the intratumoral infiltration level of CAFs in triple-negative breast cancer (TNBC) from the cancer genome atlas (TCGA) dataset using Spearman test (*n* = 191). **f**–**i** Western blot (WB) assay of LY blocking the TGF-β signaling pathway in NIH3T3 cells, and GAPDH-normalized protein expressions from (**f**, **h**) (*n* = 3 biologically independent samples). **j** Confocal laser-scanning microscopy (CLSM) examinations of TGF-β1-induced α-SMA expression (Scale bar = 8 μm, *n* = 3 biologically independent samples). **k** Masson trichrome staining (*n* = 3 mice). Scale bar = 2.5 mm (top panel) and 100 μm (bottom panel). **l** Semi-quantitative assay of collagen volume fraction (CVF) in (**k**) (*n* = 3). **m** Representative immunohistochemical staining images of CD3^+^ T lymphocytes of tumor periphery and center (*n* = 3 mice). Scale bar = 100 μm. **n** Flow cytometric analysis of the intratumoral infiltration of CD3^+^ T lymphocytes (*n* = 3). **o** Quantification of tumor-infiltrating lymphocyte (TIL) localization by immunohistochemistry (*n* = 3 mice). **p** Normalized tumor-infiltrating CD8^+^ T cells (*n* = 3 mice). Error bars represent mean ± SD. Shaded error bands depict the standard error. Violin plots represent the median, interquartile range, upper whisker, maximum, and minimum. *P* values in (**l**, **n**–**p**) derived from the Student’s *t*-test (two-tailed, two-sample unequal variance). The experiment was repeated independently three times with similar results in (**j**, **k**) and (**m**). Source data are provided as a Source Data file.

TGF-β family ligands usually initiate signaling by binding to TGF-β receptors (TGFRs), including TGFR1 and TGFR2, leading to phosphorylation of Smad transcription factors^16,17^. To identify the immune suppression role of the TGF-β signaling pathway, the cancer genome atlas (TCGA) databases analyze demonstrated that *TGFBR1* is significantly upregulated in a broad spectrum of solid tumors including breast invasive carcinoma and pancreatic adenocarcinoma tissues compared to their normal adjacent tissues (Fig. 2a, c, and Supplementary Fig. 1). Notably, the relative expression of *TGFBR1* was negatively correlated with the cumulative survival of breast cancer (BRCA) and pancreatic cancer patients (Fig. 2b, d). In addition, *TGFBR1* can increase the infiltration levels of the regulatory T cells (Tregs) and CAFs in both triple-negative breast cancer (TNBC) and pancreatic cancer tissues, and more importantly, suppress the intratumoral infiltration levels of T lymphocytes and activated dendritic cells (DCs) (Fig. 2e and Supplementary Fig. 2).

TCGA analysis of TGF-β target genes (e.g., *COL1A1*, *COL3A1*, *COL5A2*, *COL6A1*, *COL6A3*, *TIMP1*, and *CTGF*) further identified overactivation of TGF-β signaling pathway in a broad spectrum of solid tumors, in particular in breast invasive carcinoma and pancreatic adenocarcinoma tissues (Supplementary Figs. 3–8). All these genes are positively correlated with intratumoral infiltration levels of Tregs and CAFs, while negatively correlated with the infiltration levels of tumor-infiltrating effector T lymphocytes and activated DCs in both TNBC and pancreatic cancer tissues (Supplementary Figs. 9, 10). These results demonstrate that patients with the activated TGF-β signaling pathway have a poor prognosis and suppressive immune microenvironment, suggesting the promising potential of TGF-β signaling pathway as a target for cancer immunotherapy.

The TGF-β/Smad signaling pathway has been widely recognized as a key factor in tumor fibrosis^7^, and TGF-β is an important regulator of CAF activation and differentiation^18^. To validate whether TGF-β1 activates the fibroblasts and the TGF-β/Smad signaling pathway in vitro^19^, NIH3T3 cells were used as a model for fibroblast cells. Western blot (WB) assays showed that TGF-β1 promoted alpha-smooth muscle actin (α-SMA, a CAF-related protein) and phospho-Smad2/3 (pSmad2/3) expression (Supplementary Fig. 11a) in NIH3T3 cells. For instance, TGF-β1 stimulated α-SMA expression by 1.3-fold at 5.0 ng/mL (Supplementary Fig. 11b) and pSmad2/3 expression by 1.9 and 3.4-fold at 10 ng/mL and 2.5 ng/mL respectively, compared to the PBS group (Supplementary Fig. 11c, d).

However, the TGF-β1-induced CAFs activation and tumor fibrosis restrict T cells to the stroma and prevent them from accumulating in the surrounding of cancer cells through the dense ECM, which caused intrinsic immune resistance^11^. An attempt was made to use the inhibitor of TGFR1, LY to reverse the activation of the fibroblasts and relieve the activated TGF-β signaling pathway-induced intrinsic immune resistance. WB data showed that 5 μM LY efficiently reduced pSmad2/3 levels by 3.8- and 1.8-fold compared with the TGF-β1-activated groups, respectively (Fig. 2f, g, and Supplementary Fig. 11e), demonstrating that LY reversed TGF-β1-enhanced pSmad2/3 expression in NIH3T3 cells and effectively blocked the TGF-β/Smad signaling pathway.

WB results demonstrated that LY dramatically reduced the α-SMA, fibroblast activation protein (FAP), and fibronectin protein levels in NIH3T3 cells by 2.2, 2.2, and 2.6-fold, respectively (Fig. 2h, i), compared with their TGF-β1-activated groups. Similarly, LY inhibited α-SMA expression in the NIH3T3 cells as confirmed by immunofluorescence (IF) staining (Fig. 2j). These results revealed that LY reversed the TGF-β1-induced activation of the fibroblasts and blocked the TGF-β/Smad signaling pathway in vitro, thus providing crucial prerequisites for relieving tumor fibrosis and the intrinsic immune resistance induced by the activated TGF-β signaling pathway. Western blot assay in Panc02 tumor-derived primary CAFs further validated that LY reversed TGF-β1-induced expression of α-SMA, fibronectin and FAP, and consequently inactivated pSmad2/3 (Supplementary Fig. 12).

To further prove the inhibitory effect of LY on tumor fibrosis in vivo, we established a subcutaneous TNBC tumor model by injecting murine 4T1 tumor cells into the right mammary fat pad of the immune-competent BALB/c mice. LY was intratumorally injected at a dose of 0.75 or 1.5 mg/kg when the tumor volume reached 100 mm^3^. One week after the drug administration, Masson’s staining of the tumor sections showed that 1.5 mg/kg LY significantly relieved collagen fiber generation in the tumor tissues (Fig. 2k, l). Moreover, immunohistochemistry (IHC) and flow cytometry examination displayed that LY significantly increased the tumor infiltration of CD3^+^ T cells (CD45^+^CD3^+^) and CD8^+^ T cells (CD45^+^CD3^+^CD8^+^). Quantitative analysis of histopathology demonstrated that CD3^+^ T cells distribution significantly changed following intratumoral LY (1.5 mg/kg) therapy, and the mean distance between CD3^+^ T cells from the stromal border was increased, indicating that LY inhibited tumor fibrosis and relieved the dense “physical barrier” of the tumor tissue to promote the intratumoral infiltration of the T lymphocytes (Fig. 2m–p and Supplementary Fig. 11f). Noticeably, LY treatment at a dose of 1.5 mg/kg dramatically reduced ~2.0-fold of M2-type tumor-associated macrophages (TAM) to increase M1-type macrophages by ~5.0-fold, indicating that TGFR1 inhibition can also relieve TAMs-induced immune resistance (Supplementary Fig. 13).

### Construction and characterization of LY-loaded nanovesicles

**Fig. 3:**
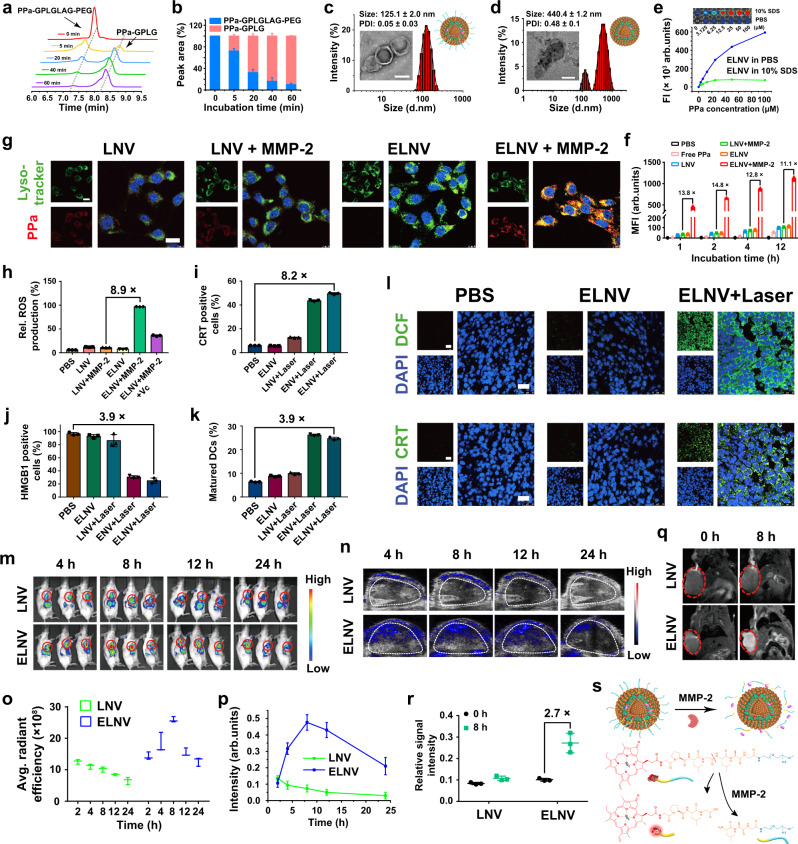
Physio-chemical characterizations and multimodality imaging of the nanovesicles in vitro and in vivo. **a**, **b** Time-dependent **a** high-performance liquid chromatography (HPLC) chromatogram and **b** degradation ratio of PPa-GPLGLAG-PEG after incubation with MMP-2 (*n* = 3 independent experiments). **c**, **d** Hydrodynamic diameters and representative transmission electron microscope (TEM) images of **c** ELNV and **d** ELNV nanovesicles incubated with MMP-2. Scale bars = 100 nm. **e** Fluorescence property of ELNV (*n* = 3 independent experiments). The inset graph shows the fluorescence imaging of ELNV. **f**, **g** Flow cytometry and CLSM analysis of the cellular uptake of PPa in different groups (*n* = 3 biologically independent samples). Scale bars = 20 μm. **h** Flow cytometric analysis of intracellular ROS generation in 4T1 cells (*n* = 3 biologically independent samples). **i**, **j** PDT-triggered CRT exposure and HMGB1 efflux in 4T1 cells (*n* = 3 biologically independent samples). **k** The percentage of PDT-induced DC maturation in vitro. Immature DCs were incubated with pre-treated tumor cells for 24 h (*n* = 3 biologically independent samples). All samples were pretreated with 40 μg/mL MMP-2 before adding to the cells in (**f**–**k**). **l** CLSM examination of ROS generation and CRT exposure in 4T1 tumor ex vivo. Scale bars = 25 μm. **m**, **n** Fluorescence and PA images of 4T1 tumor-bearing mice after intravenous injection with LNV or ELNV for different times. The red or white circles indicate the tumor sites. **o**, **p** Time course quantifications of the **o** fluorescence and **p** PA signal intensity at tumor region in (**m**) and (**n**), respectively (*n* = 3 mice). **q** *T*_1_-weighted MRI of tumor-bearing mice before and 8 h after intravenous injection with LNV and ELNV. Red circles indicate tumor sites. **r** Quantification of *T*_1_-weighted MRI signal intensity in the tumor region in (**q**) (*n* = 3 mice). **s** Mechanism of enzyme-activatable multimodality imaging of ELNV. Error bars represent mean ± SD. The experiment was repeated independently 3 times with similar results in (**c**, **d**) and (**l**). DCF dichlorodihydrofluorescein, Avg. average, Rel. relative. Source data are provided as a Source Data file.

Given the remarkable reversion and inhibitory effect of LY on CAFs and the TGF-β/Smad signaling pathway, the next was to accomplish the tumor-specific delivery of LY for recruiting tumor-specific T lymphocytes. MMP-2 plays key roles in angiogenesis, tumor invasion, and metastasis^20^, which is highly expressed in a wide variety of solid tumors including human and murine breast and pancreatic carcinomas (Supplementary Figs. 14–16). Thus, an MMP-2-sensitive nanovesicle was engineered by integrating the photosensitizers Pyropheophorbide a (PPa) and LY into a nanoplatform. PPa was covalently conjugated to a poly(ethylene glycol) (PEG) chain using an enzyme-sensitive peptide spacer (GPLGLAG) through a two-step amidation reaction. Moreover, MMP-2-insensitive PPa-PEG_5k_ was synthesized and used as the negative control. The successful synthesis of PPa-GPLGLAG-PEG_5k_ and PPa-PEG_5k_ was verified by proton nuclear magnetic resonance (^1^H-NMR) and matrix-assisted laser desorption/ionization time-of-flight mass spectrometry (MALDI-TOF MS) (Supplementary Figs. 17–19). High-performance liquid chromatography (HPLC) and MALDI-TOF MS analysis revealed that ~90% of PPa-GPLGLAG-PEG_5k_ was degraded at the Leu-Gly site after a 60 min incubation with 40 μg/mL MMP-2, verifying the higher MMP-2 sensitivity to the GPLGLAG peptide spacer (Fig. 3a, b, and Supplementary Fig. 20).

LY-loaded enzyme-sensitive nanovesicles (ELNV) and their enzyme-insensitive analogs (LNV) were then prepared using the film hydration method with an LY encapsulation efficiency of up to 94% (Supplementary Table 5). Transmission electron microscopy (TEM) and dynamic light scattering (DLS) revealed the spherical morphology and 125.1 ± 2.0 nm hydration particle size of the ELNV nanovesicles (Fig. 3c). The PPa encapsulation efficiency and loading ratio were determined to be >98.0% and ~2.0% in both ELNV and LNV nanovesicles, respectively (Supplementary Table 2). The ELNV nanovesicles displayed constant particle size and size distribution after 24 h incubation with 10% fetal bovine serum (FBS) solution at 37 °C or 6-days storage at 4 °C (Supplementary Fig. 21a, b), implying superior serum stability of the nanovesicles.

To demonstrate the de-PEGylation properties of the ELNV and LNV nanovesicles, the dissociation of the spatial structure of ELNV was visualized by TEM and DLS (Fig. 3d). ELNV displayed increased size and PDI upon MMP-2 incubation, indicating de-PEGylation (Supplementary Fig. 21c, d). In contrast, the morphology and structure of the LNV vesicles remained unchanged (Supplementary Fig. 21e). A negligible amount of LY was released from the LNV nanovesicles in the presence of MMP-2. In contrast, MMP-2 incubation remarkably accelerated LY release from the ELNV nanovesicles (Supplementary Fig. 21f), owing to dePEGylation-induced destabilization of the ELNV nanovesicles. PPa was barely released from the ELNV and LNV nanovesicles since PPa was stably encapsulated inside the lipid bilayer via hydrophobic interaction (Supplementary Fig. 22).

Fluorescence imaging (FI) of the ELNV nanovesicles showed aggregation-caused quenching of the PPa fluorescence signal, while the fluorescence was restored ~8-fold in 10% sodium dodecyl sulfate solution at 100 μM PPa (Fig. 3e, and Supplementary Fig. 23a). Furthermore, singlet oxygen sensor green (SOSG) as a ROS probe was used to investigate the photoactivity of the nanovesicles by monitoring reactive oxygen species generation^21,22^. ELNV nanovesicles induced singlet oxygen (^1^O_2_) generation at a PPa concentration- and the photodensity-dependent manner in the PBS containing 10% SDS (Supplementary Fig. 23b, c), indicating that dissociated nanovesicles have superior photodynamic activity for antitumor therapy.

ELNV showed negligible cytotoxicity in 4T1 cells in vitro (Supplementary Fig. 23d). Moreover, flow cytometry measurements showed that the cellular uptake of ELNV positively correlates with the incubation time, and the intracellular PPa intensity of the ELNV + MMP-2 group was 11.1-fold higher than that of the LNV + MMP-2 group after 12 h (Fig. 3f). CLSM images further showed the highest PPa fluorescence intensity in the MMP-2 + ELNV group, which co-localized with lysosomes (Fig. 3g). This suggests that MMP-2-responsive de-PEGylation enhanced the cellular uptake of ELNV and provided the essential conditions for activating the tumor-specific immune response.

Subsequently, we investigated ROS generation in the ELNV nanovesicles in 4T1 cells. The photodynamic therapy (PDT)-induced ROS generation in the ELNV + MMP-2 group was 8.9-times higher than that in the LNV + MMP-2 group under the 671 nm laser irradiation, due to rapid uptake by the cells of the dePEGylated ELNV (Fig. 3h, Supplementary Fig. 24a, b). After incubation with MMP-2, the ELNV nanovesicles induced ^1^O_2_ production in vitro in a PPa concentration- and photodensity-dependent manner (Supplementary Fig. 24c). The generation of cytotoxic ROS is critical for the photodynamic cancer cell death. The results showed 38.57 ± 2.5% cell survival based only on the PDT effect of the ELNV treatment at a PPa concentration of 5 μM and photodensity of 150 mW/cm^2^ (Supplementary Fig. 24d). This indicated that PDT alone was adequate to kill tumor cells at a safe dose. Noticeably, ELNV nanovesicles displayed much higher photoactivity than free PPa in vitro due to increased cellular uptake when delivered by the nanovesicles (Supplementary Fig. 25, Fig. 3h).

Next, we investigated the ability of PDT to induce ICD by determining the cell-surface expression of calreticulin (CRT) and nucleus high mobility group protein B1 (HMGB1) efflux in vitro^23,24^. There was almost no CRT signal in the PBS, ELNV, and LNV + Laser groups, whereas the ENV + Laser or ELNV + Laser groups dramatically promoted CRT expression on the surface of the tumor cells (Supplementary Fig. 26a), indicating that enzyme-sensitive nanovesicles, whether loaded with LY or not, can both induce cell apoptosis and CRT exposure on the surface of 4T1 cells. Similarly, the intracellular HMGB1 fluorescence was 3.9 times lower (Fig. 3j) in the ELNV + Laser group than in the PBS group. Flow cytometry assay revealed that ELNV + Laser increased the percentage of CRT-positive cells in the group by 8.2-fold relative to PBS (Fig. 3i, and Supplementary Fig. 26a, b). DCs are specialized antigen-presenting immune cells^25^. DC maturation is an indicator of ICD-induced antitumor immune responses^26^. The ELNV + Laser groups induced 24.7 ± 0.6% DCs maturation (Fig. 3k, and Supplementary Fig. 26c), which was 3.9-fold higher than that of the PBS group. The above results demonstrated that PDT with ELNV could promote CRT exposure, HMGB1 release, and DC maturation, thus increasing the immunogenicity of 4T1 cells.

To validate the PDT-activated ICD effect in vivo, ROS production and CRT exposure on the cell surface of the tumor cells were assessed before and after laser irradiation. Consistent with the in vitro results, upon NIR laser irradiation, the enzyme-activated nanovesicles induced more significant ROS generation and CRT exposure in the tumor than in the control groups, indicating that the ELNV + Laser group could activate the anti-cancer immune responses in vivo (Fig. 3l, and Supplementary Fig. 26d–g). PPa formulated inside the ELNV nanovesicles displayed much higher ICD induction efficiency and phototoxicity than free PPa by increasing intracellular delivery of poorly water-soluble photosensitizer (Supplementary Figs. 27, 28).

### Tumor-specific LY delivery and multimodal imaging of the nanovesicles

To investigate the pharmacokinetic profiles and biodistribution of the ELNV nanovesicles in vivo, the ELNV nanovesicles were intravenously (i.v.) injected at an LY dose of 20 mg/kg and PPa dose of 5 mg/kg. ELNV design indeed prolongated the blood-elimination half-life (t_1/2β_) of both LY and PPa in blood circulation (Supplementary Figs. 29a–d). For instance, the PPa and LY in the ELNV nanovesicles have t_1/2β_ of 12.48 ± 3.0 and 17.36 ± 2.0 h, which are 14.5 and 10.7-times longer than that of free PPa and LY, respectively. Moreover, the bioavailability (area under the time-concentration curve, AUC_(0-t)_) of PPa and LY remarkably increased by 14.2 and 30.8-fold in the ELNV group compared to free PPa and LY, respectively (Supplementary Table 4). Furthermore, ELNV enhanced intratumoral distribution of PPa and LY by 29.7 and 19.5-fold respectively in subcutaneous 4T1 tumors at 6 h-post the administration of the drugs. These results indicated that the PEG shell dramatically decreased the clearance of nano-drugs by the mononuclear phagocytic system in blood and significantly improved nano-drug utilization and stability in blood circulation (Supplementary Fig. 29e). Therefore, the sheddable nanovesicles could increase the tumor accumulation of PPa and LY compared with free PPa and LY, respectively, and diminish the accumulation of drugs in normal tissues, thus reducing toxic side effects^27,28^.

FI in vivo showed that ELNV nanovesicles gradually accumulated at the tumor sites and reached a peak 8 h after i.v. injection. In contrast, the LNV group was marginally distributed at the tumor site, which suggests that PEG in the lipid layer hindered cellular uptake of the nanovesicles (Fig. 3m, o, and Supplementary Fig. 29f). The FI of the tumor tissue and major organs (i.e., heart, liver, spleen, lung, and kidney) ex vivo 24 h after injection revealed significant tumor accumulation of the ELNV nanovesicles (Supplementary Fig. 30a). The IF staining of the tumor sections further proved that ELNV nanovesicles with laser irradiation penetrated over the perivascular region to the deep parts of a tumor, and the improved penetration would also lead to better therapeutic efficacies (Supplementary Fig. 30b, c).

Moreover, the tumor accumulation of the ELNV nanovesicles was investigated using in vivo photoacoustic imaging (PAI)^29^. The PAI signal showed a linear enhancement with increasing concentration of the ELNV nanovesicles in vitro, and similar phenomena were observed in the LNV nanovesicles (Supplementary Fig. 30d). The PAI signal of the ELNV nanovesicles in the tumor tissue increased over time and reached a maximum after 8 h, which agreed well with the fluorescence imaging data (Fig. 3n). The quantification analysis of the PAI signal at the tumor region 8 h after injection revealed a 6.8-times higher tumor distribution of the ELNV nanovesicles than the LNV counterpart (Fig. 3p).

Next, the *T*_1_-weighted magnetic resonance imaging (MRI) phantom of Gd^3+^-loaded ELNV nanovesicles was examined in a 9.4 T small-animal magnetic field (Supplementary Table 3). The MRI signal intensity of the ELNV nanovesicles increased as a function of Gd^3+^ concentration upon MMP-2 treatment; however, the enzyme-insensitive nanovesicles showed a much lower MR signal (Supplementary Fig. 30e, f). Figure 3q showed that the ELNV nanovesicles generated much higher *T*_1_ contrast in 4T1 tumor-bearing mice than LNV because of the enzyme-activatable MRI property of the ELNV nanovesicles. The Gd^3+^-concentration-normalized *T*_1_ signal of the ELNV group was 2.7-times higher than that of the LNV group after 8 h (Fig. 3r). These results demonstrate the potential of ELNV nanovesicles for FI, MRI, and PAI three-modal imaging-guided precise PDT (Fig. 3s)^30^.

### ELNV nanovesicles attenuated tumor fibrosis and facilitated tumor infiltration of CTLs

**Fig. 4:**
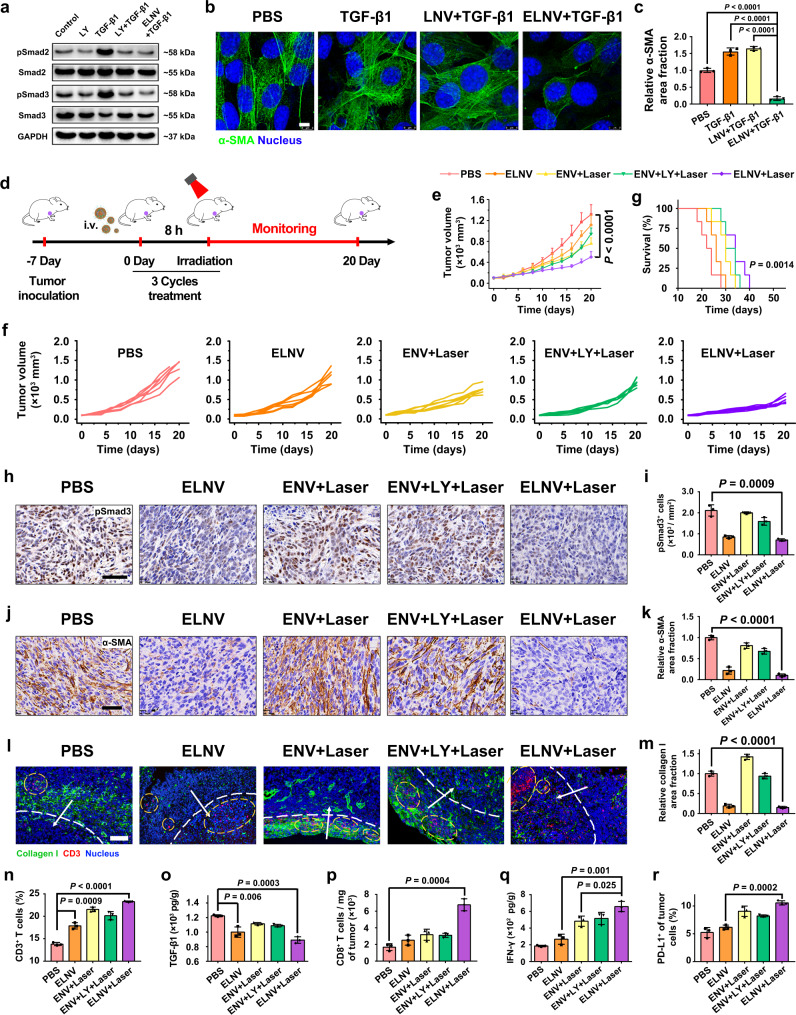
ELNV nanovesicles breached the tumor fibrosis and promoted the antitumor immunity in TNBC. **a** WB analysis of ELNV-mediated blocking of TGF-β signaling pathway. The experiment was repeated independently three times with similar results. **b** CLSM examinations of α-SMA expression (Scale bar = 8 μm). **c** Semi-quantitative analysis of the relative α-SMA area fraction in (**b**) by Image J (*n* = 3 biologically independent samples, *P* = 6.11 × 10^−5^, 3.48 × 10^−5^ and 5.80 × 10^−6^). **d** Treatment schedule of ELNV-mediated combination photoimmunotherapy. **e**–**g** Anti-tumor effective of ELNV nanovesicles. **e**, **f** Tumor growth and **g** survival curves of 4T1 tumor-bearing mice (*n* = 6 mice, *P* = 2.01 × 10^−5^). **h** Immunohistochemical staining of pSmad3 in the excised tumors. Scale bar = 50 μm. **i** Semi-quantitative analysis of pSmad3^+^ cells (*n* = 3 mice). **j** Immunohistochemical staining of α-SMA in the excised tumors. Scale bar = 50 μm. **k** Semi-quantitative analysis of the relative α-SMA area fraction (*n* = 3 mice, *P* = 1.46 × 10^−5^). **l** IF staining of collagen I and CD3^+^ T lymphocytes in 4T1 tumors. Scale bar = 100 μm. The white dotted lines distinguish the peripheral regions from the central regions of the tumors. White arrows indicate the directions of the CD3^+^ T cell infiltration, and yellow circles highlight the regions of CD3^+^ T cells. **m** Semi-quantitative analysis of relative collagen I area fraction (*n* = 3 mice, *P* = 2.79 × 10^−5^). **n** Flow cytometric analysis of the intratumoral infiltration of CD3^+^ T lymphocytes (*n* = 3 mice, *P* = 8.73 × 10^−4^ and 1.94 × 10^−6^). **o** ELISA analysis of intratumoral TGF-β1 secretion (*n* = 3 mice). **p** Normalized tumor-infiltrating CD8^+^ T cells (*n* = 3 mice). **q** ELISA analysis of intratumoral IFN-γ secretion (*n* = 3 mice). **r** The frequency of PD-L1 expression on the surface of tumor cells (*n* = 3 mice). Error bars represent mean ± SD. *P* values in all panels except **g** derived from the Student’s *t*-test (two-tailed, two-sample unequal variance). In panel **g**, the Log-rank (Mantel–Cox) test was used. Source data are provided as a Source Data file.

To further explore the impacts of ELNV nanovesicles on TGF-β/Smad signaling pathway and whether ELNV could also reverse fibroblasts activation, a WB assay was performed to detect the expression of the downstream effector protein of the TGF-β/Smad signaling pathway. First, the cytotoxicity experiment results of NIH3T3 fibroblast showed good biocompatibility with the ELNV nanovesicles (Supplementary Fig. 31a). WB assay showed that the ELNV nanovesicles significantly downregulated pSmad2/3 expression in NIH3T3 cells in vitro due to the MMP-2-triggered rapid LY release from the nanovesicles (Fig. 4a, Supplementary Fig. 31b, c), thus blocking the TGF-β signaling pathway. In addition, ELNV nanovesicles also reversed the expression of α-SMA, FAP, and fibronectin in NIH3T3 cells (Supplementary Fig. 31d, e). Moreover, IF staining showed a dramatic α-SMA reduction in the ELNV + TGF-β1 group, which agreed well with the WB assay (Fig. 4b, c). These results indicate that the ELNV nanovesicles effectively blocked the TGF-β/Smad signaling pathway to attenuate tumor fibrosis and reverse CAFs activation to reduce ECM deposition.

The antitumor effects of PDT on ELNV nanovesicles were next examined in a subcutaneous 4T1 breast cancer mouse model (Fig. 4d). Compared with the controls (including the single drug nanovesicle, ENV + Laser group), ELNV with laser potently inhibited tumor growth; however, the tumors redeveloped at the end of the treatment (Fig. 4e, f and Supplementary Fig. 32a, b). Thus, the mice with ELNV + Laser treatment did not prolong survival significantly, with all animals lost within 40 days (Fig. 4g). Hematoxylin and eosin (H&E) staining confirmed the tumor-killing effect of the ELNV with laser irradiation (Supplementary Fig. 32g). Negligible body weight changes were observed during the experiment period (Supplementary Fig. 32c). ELNV + Laser group significantly suppressed the metastasis of 4T1 cells to the lungs (Supplementary Fig. 32d–f). Collectively, these results indicate that ELNV nanovesicles, at least in part, suppressed primary tumor growth and metastasis in mouse models of breast carcinoma.

The moderate antitumor activity of ELNV nanovesicles prompted us to further explore the mechanisms underlying their immunomodulatory effects. Immunohistochemical staining revealed that the expression of the phosphorylated Smad3 was significantly reduced in the ELNV and ELNV + Laser groups (Fig. 4h, i), suggesting that ELNV effectively blocked the TGF-β/Smad signaling pathway in tumor tissues. ELNV and ELNV + Laser treatments significantly reduced the α-SMA, FAP, fibronectin, and Collagen I content, signifying the deactivation of CAFs (Fig. 4j, l, Supplementary Fig. 33a, c). For example, ELNV + Laser treatment reduced 89.7, 68.3, 53.3, and 84.9% of α-SMA, FAP, fibronectin, and collagen I expression, respectively, compared to the PBS group (Fig. 4k, m, Supplementary Fig. 33b, d). Collectively, these results demonstrated that ELNV + Laser treatment reversed the TGF-β1-triggered activation of CAFs and fibrosis in TNBC tumors. Furthermore, ELNV and ELNV + Laser treatments significantly increased the number of infiltrating CD3^+^ T cells compared with PBS (Fig. 4l, n), whereas ELNV + Laser-induced higher T cell infiltration than ELNV alone. This result revealed that promoting tumor infiltration of T lymphocytes requires two preconditions: First, ELNV nanovesicles need to stimulate the immune system to prime and recruit T lymphocytes to the tumor sites; second, ELNV nanovesicles should be able to break the biophysical barrier of solid tumor to enable the infiltration of CTLs^31^. It was worth noting that PDT by ENV or free LY + ENV nanovesicle slightly affected collagen I expressed and marginally reduced the ECM burden. In contrast, LY-loaded ELNV nanovesicles dramatically reduced collagen I level in vivo, and subsequently promoted intratumoral infiltration of CD8^+^ T lymphocytes (Fig. 4p), suggesting the crucial role of nanovesicle-delivered LY for promoting intratumoral infiltration of CTLs.

Next, ICD-induced immune response was evaluated by measuring DC maturation in the lymph nodes in vivo^32^. The weight of the lymph nodes was markedly increased after PDT (Supplementary Fig. 34a), indicating the effective induction of an antitumor immune response in vivo, irrespective of LY. The ELNV + Laser group displayed a 2.4 and 2.1-times higher maturated DC ratio (CD11c^+^CD80^+^CD86^+^, 21.9 ± 1.5%) than that of the PBS and ELNV groups, respectively (Supplementary Fig. 34b, c), proving that photoimmunotherapy significantly promoted DC maturation.

TGF-β1 secretion in tumors was determined by the enzyme-linked immunosorbent assay (ELISA). Surprisingly, TGF-β1 expression in tumor tissues decreased in the ELNV groups irrespective of NIR (Fig. 4o). Therefore, this may be caused by the fact that ELNV nanovesicles reverse the activation of CAFs and inhibit tumor fibrosis through the TGF-β/Smad signaling pathway, which further reduces TGF-β1 secretion in tumors. The ENV + Laser and ELNV + Laser groups had more tumor infiltration of CD8^+^ T cells than the PBS and ELNV groups. However, the number of CD8^+^ T cells in the ENV + Laser group was lower than that in the ELNV + Laser group, suggesting that reducing extracellular matrix deposition is beneficial to the tumor tissue infiltration of CD8^+^ T cells (Fig. 4p, and Supplementary Fig. 34d). The ELNV + Laser group induced the highest percentage of tumor-infiltrating effector T cells (CD45^+^CD3^+^CD8^+^IFN-γ^+^) (up to 17.3 ± 1.3%), which therefore activated a strong antitumor immunity and suppressed the tumor growth (Supplementary Fig. 34e, f).

Tregs are the major components respond for immune suppression and can also secret TGF-β1^33^. PDT consumes oxygen and induces hypoxia condition in the tumor microenvironment, causes M2 polarization of TAM, and upregulates Tregs by secreting TGF-β^16^. The proportion of Tregs (CD3^+^CD4^+^Foxp3^+^) from 21.0 ± 1.6% in the PBS group to 38.6 ± 4.8% in the ENV + Laser group, which was reduced to 18.8 ± 1.3% in the ELNV + Laser group, demonstrated that PDT upregulated the number of Tregs, while the ELNV + Laser group could improve the PDT-induced ITM, which caused 2.6-times higher CD8^+^ T cells/Tregs ratio of ELNV + Laser group than that of the ENV + Laser group (Supplementary Fig. 34g, h), suggesting that TGF-β1 inhibition in the ELNV + Laser group reduces tumor-infiltrating Tregs.

Intratumoral IFN-γ secretion was assessed by ELISA for the evaluation of immunostimulatory effects^34,35^. Figure 4q shows that in general, IFN-γ secretion significantly increased in the ENV/ELNV + Laser group compared with the PBS and ELNV groups, and the amount of IFN-γ in the ELNV + Laser group was 3.5-fold higher than that in the PBS group. Administered together, the photodynamic immunotherapy of ELNV could relieve tumor fibrosis, induce the ICD effect in tumors, promote the maturation and infiltration of T cells and ultimately enhance the antitumor immune response in vivo. However, the tumor-infiltrating effector T cells induced the overexpression of PD-L1 on the surface of tumor cells by secreting IFN-γ, which in turn, caused inducible immune resistance and recurrence of the tumor^36^. Flow cytometry data indicated that the ELNV + Laser group indeed upregulated PD-L1 on the surface of tumor cells over twofold that of the PBS group (Fig. 4r, and Supplementary Fig. 34i). Hence, although PDT with the ELNV nanovesicles provided the recruitment signals of tumor-specific T cells to the tumors, and breached the physical barrier of T cells infiltration, the infiltrated T cells were inactivated by the inducible immune resistance, leading to the failure of immunotherapy^5^.

### Nanovesicles overcame intrinsic and adaptive immune resistance in multiple tumors

Next was to integrate the ROS-responsive JQ1 prodrug into the ELNV nanovesicles to address the IFN-γ-induced PD-L1 activation and adaptive immune resistance. JQ1 is a potent inhibitor of bromodomain-containing protein 4 (BRD4), which abolishes IFN-γ-induced PD-L1 expression on the surface of tumor cells, thus overcoming the acquired immune suppression^37^. To achieve tumor-specific JQ1 delivery and BRD4 inhibition, JQ1 was covalently linked with the hydroxyl group of 1-palmitoyl-2-hydroxy-sn-glycero-3-phosphocholine (p-lysoPC) via a thioketal group (Supplementary Figs. 35–38). Meanwhile, a ROS-insensitive JTP analog, namely JP was synthesized by conjugating JQ1 onto p-lysoPC with a triethylene glycol (Tg) spacer (Supplementary Figs. 39, 40). JTP was readily activated with 671 nm laser-performed PDT to release JQ1-SH. Liquid chromatograph mass spectrometer (LC-MS) analysis revealed that nearly 80% of JTP degraded to JQ1-SH after 5 min of irradiation with the 671 nm laser at a photodensity of 150 mW/cm^2^ (Supplementary Figs. 41a–d), verifying superior ROS sensitivity of the thioketal linker. While JQ1-Tg-pPC displayed satisfying photostability in a high-level ROS environment (Supplementary Fig. 41f–h). The rapid and effective light-response properties of JQ1 release provide a precondition for quickly relieving inducible immune resistance in vivo. The EJNV nanovesicles also have excellent biocompatibility (Supplementary Fig. 41e).

Subsequently, the ELJNV nanovesicles were prepared by the thin-film hydration method^38^. The addition of JQ1-thioketal (TK)-pPC (JTP) did not affect the spherical structure of the ELNV nanovesicles and provided an approach for constructing multifunctional nanovesicles. The PPa trapped within the nanovesicles can induce notable ROS generation upon NIR laser irradiation, in turn, cut off ROS-responsive thioketal linkages in JTP to release JQ1 (Scheme 1)^39^. TEM and DLS results indicated that ELJNV nanovesicles were of uniform nanostructure with an average particle size of ~121.5 ± 4.3 nm (Supplementary Fig. 42a). Upon MMP-2 incubation, the nanovesicles were dePEGylated and fused into heterogeneous nanostructures (Supplementary Fig. 42b). Both ELNV and ELJNV displayed comparable LY release profile in the presence of MMP-2 (Supplementary Fig. 42c), suggesting JTP loading negligibly affected LY release property of the nanovesicles. The ELJNV nanovesicles displayed satisfying serum stability in 10% FBS solution and whole blood serum (Supplementary Fig. 42d, e), which was comparable to the JTP-free ELNV parental nanovesicles, implying their potential for tumor-specific LY and JQ1 co-delivery at the consistent drug loading ratio.

Free JQ1 and JQ1-SH released from JTP comparably inhibit IFN-γ-inducible PD-L1 expression on the surface of the tumor cell membrane in both 4T1 and Panc02 tumor cells in vitro. In contrast, JP or JP + Laser marginally affected IFN-γ-inducible PD-L1 expression in vitro, validating the potential of JTP for PDT-triggered tunable JQ1 release at the tumor site. Flow cytometry examination showed that JTP+Laser instead of JP+Laser highly efficiently inhibits IFN-γ-inducible PD-L1 expression on the surface of the tumor cell membrane as free JQ1 in both 4T1 and Panc02 tumor cells in vitro (Supplementary Fig. 43), validating PDT-activatable BRD4 inhibition profile of JTP prodrug. The 671 nm laser pretreated EJNV nanovesicles highly efficiently inhibitory effect on downregulated IFN-γ-induced PD-L1 overexpression on both surfaces of 4T1 (3.6-fold) or Panc02 cells (6.1-fold), validating the PDT-triggered rapid and sensitive drug release of the nanovesicles to abolish PD-L1 expression in vitro (Supplementary Fig. 44).

**Fig. 5:**
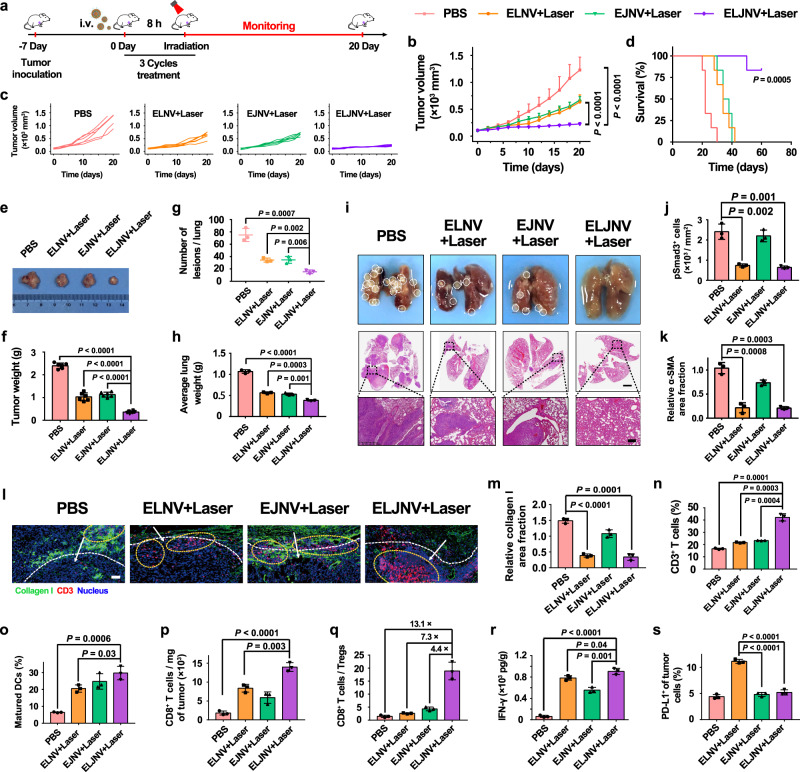
ELJNV nanovesicles enhanced the antitumor immune responses by blocking the TGF-β signaling pathway and PD-L1 expression in TNBC tumors. **a** Treatment protocol of ELJNV-mediated combination photoimmunotherapy. **b**–**i** Anti-tumor effective of ELJNV nanovesicles. **b**, **c** Tumor growth and **d** survival curves of mice with TNBC tumors (*n* = 6 mice, *P* = 2.81 × 10^−6^ and 2.10 × 10^−5^). **e** Representative photographs and **f** the average weights of excised tumors (*n* = 6 mice, *P* = 4.08 × 10^−12^, 4.63 × 10^−6^ and 2.31 × 10^−8^). **g** The average number of lung tumors and **h** lung weights (*n* = 6 mice, *P* = 7.19 × 10^−4^, 1.76 × 10^−3^ and 6.02 × 10^−3^ in (**g**), *P* = 1.17 × 10^−5^, 2.70 × 10^−4^ and 1.14 × 10^−3^ in (**h**)). **i** Typical images and H&E staining of lung tissues. Scale bar = 2 mm and 200 μm (enlarged image). **j**, **k** Semi-quantitative analysis of pSmad3^+^ cells and relative α-SMA area fraction in the excised tumors (*n* = 3 mice). **l** IF staining of collagen I and CD3^+^ T lymphocytes. Scale bar = 50 μm. The meanings of white dotted lines, arrows, and yellow circles are the same as in Fig. 4l. **m** Semi-quantitative analysis of the relative collagen I area fraction (*n* = 3 mice, *P* = 2.85 × 10^−5^ and 1.04 × 10^−4^). **n** Flow cytometric analysis of the intratumoral infiltration of CD3^+^ T lymphocytes (*n* = 3 mice). **o** The frequency of maturated DCs in the tumor-draining lymph nodes (*n* = 3 mice). **p** Normalized tumor-infiltrating CD8^+^ T cells (*n* = 3 mice, *P* = 7.83 × 10^−5^ and 3.39 × 10^−3^). **q** The ratio of CD8^+^ T cell-to-Treg (*n* = 3 mice). **r** ELISA analysis of IFN-γ in tumor tissues (*n* = 3 mice, *P* = 1.86 × 10^−5^, 4.24 × 10^−2^ and 1.40 × 10^−3^). **s** The frequency of PD-L1 expression on tumor cells in vivo (*n* = 3 mice, *P* = 3.35 × 10^−5^ and 8.11 × 10^−5^). Error bars represent mean ± SD. *P* values in all panels except **d** derived from the Student’s *t*-test (two-tailed, two-sample unequal variance). In panel **d**, Log-rank (Mantel–Cox) test was used. Source data are provided as a Source Data file.

The efficacy of ELJNV in suppressing 4T1 tumor growth was investigated in BALB/c mice (Fig. 5a). After treatment, the mice were sacrificed, and the tumors were excised. The tumor volumes, tumor representative images, tumor weights, and mice survival curves showed that the ELJNV + Laser group potently inhibited tumor growth (Fig. 5b–f) and significantly prolonged survival (Fig. 5d). In contrast, the ELNV + Laser and EJNV + Laser groups did not inhibit tumor growth at a later stage of treatment (Fig. 5b, c). Meanwhile, the body weights of the mice after different treatments remained almost constant (Supplementary Fig. 45a), suggesting minimal systemic toxicity of the treatments. The excised lungs were weighed and imaged. The results showed that there was almost no lung metastasis in mice treated with ELJNV + Laser (Fig. 5g–i). Furthermore, H&E staining suggested that the damage to healthy tissues, including the liver, was minimal in all treatments (Supplementary Fig. 45b). In contrast, H&E staining demonstrated obvious tumor cell apoptosis/necrosis by ELJNV + Laser (Supplementary Fig. 45c), which was consistent with the inhibition of tumor growth by ELJNV + Laser in mice (Fig. 5b, c). These results validated the potent inhibition of 4T1 tumor growth by ELJNV nanovesicles.

The expression of pSmad3 in the tumor sections was significantly decreased in the ELNV and ELJNV groups (Fig. 5j, and Supplementary Fig. 45d), indicating that the ELNV and ELJNV nanovesicles dramatically inhibited TGF-β1-induced fibrosis through the TGF-β/Smad signaling pathway in vivo. Furthermore, ELJNV nanovesicles also reduced the expression of α-SMA, FAP, fibronectin, and collagen I in tumor tissues (Fig. 5k–m, and Supplementary Fig. 45e–i). For instance, the fibronectin area decreased by 83.3% in the ELJVN + Laser group compared to that in the PBS group. Therefore, ELJNV reverses the activation of CAFs, breaches the ECM barrier, and enables T-cell transmigration in vivo. These analyses are in accordance with the above experimental results. Subsequently, the enhancement of the antitumor immune response upon relieving the “physical barrier” and inhibiting the overexpression of PD-L1 on the surface of tumor cells was evaluated. The expression of collagen I was significantly decreased in both ELNV + Laser and ELJNV + Laser groups, whereas the number of CD3^+^ T cells infiltrated in the ELJNV + Laser group was much higher than that in the ELNV + Laser group (Fig. 5l–n).

Flow cytometry data demonstrated that ELJNV + Laser recruited 42.2 ± 3.0% of CD3^+^ T cells in tumor tissues, which was a 2.0 and 1.8-fold increase compared with the ELNV + Laser and EJNV + Laser groups, respectively (Fig. 5n). This result demonstrated that the blockade of PD-L1 significantly reinvigorated CD3^+^ T cell activities, thereby inducing more infiltrating T cells and promoting antitumor immune responses. However, because of the hindrance of the dense extracellular matrix, almost no T cells were trafficked into the tumor in the EJNV + Laser group. Moreover, the weight of the inguinal and axillary lymph nodes in the ELJNV + Laser group was 1.5 and 1.6 times higher than that in the ELNV + Laser and EJNV + Laser groups, respectively (Supplementary Fig. 46a). DCs maturation in tumor-draining lymph nodes of the PBS group (6.5 ± 0.3%) was upregulated by 4.6-fold after ELJNV + Laser treatment (29.7 ± 4.0%) (Fig. 5o, Supplementary Fig. 46b).

Moreover, ELJNV + Laser treatment also significantly promoted the intratumoral infiltration fraction of CD8^+^ and IFN-γ^+^CD8^+^ effector T lymphocytes, which increased from 22.1 ± 3.6 and 3.5 ± 0.7% to 78.0 ± 2.8 and 28.9 ± 4.2%, respectively, compared with the PBS groups (Fig. 5p, and Supplementary Fig. 46c–e). The ELJNV nanovesicles with laser also dramatically inhibited the ratio of Tregs (4.2 ± 0.6%), which decreased 4.6-fold compared to that in the PBS group (Supplementary Fig. 46f). Therefore, the ratio of CD8^+^ T cells to Tregs in the tumor tissues of the ELJNV + Laser group dramatically increased 13.1-fold compared to that in the PBS group (Fig. 5q), as previously shown.

ELISA showed that PDT with ELNV, EJNV or ELJNV led to intratumoral secretion of large amounts of IFN-γ (Fig. 5r), indicating the promotion of tumor inflammation and indirect killing of tumor cells. For instance, the IFN-γ content in the ELNV + Laser and ELJNV + Laser groups was 12.4 and 14.3-fold higher than that in the PBS group, respectively. However, IFN-γ is well known for its ability to upregulate PD-L1 expression^40^. In turn, PD-L1 binds to PD-1, which is expressed on the T cell surface, to inhibit T cell activity^41^. Flow cytometry examination showed that PD-L1 expression on the surface of the tumor cell in the ELNV + Laser group was dramatically upregulated, while EJNV or ELJNV groups showed little upregulation of PD-L1 expression (Fig. 5s, and Supplementary Fig. 46g). This is due to the spatiotemporal release of the BRD4 inhibitor JQ1 from ELJNV in a laser-light-controlled manner, which simultaneously causes antitumor immunity and blocks the PD-L1/PD-1 checkpoint to prevent tumor recurrence and metastasis.

**Fig. 6:**
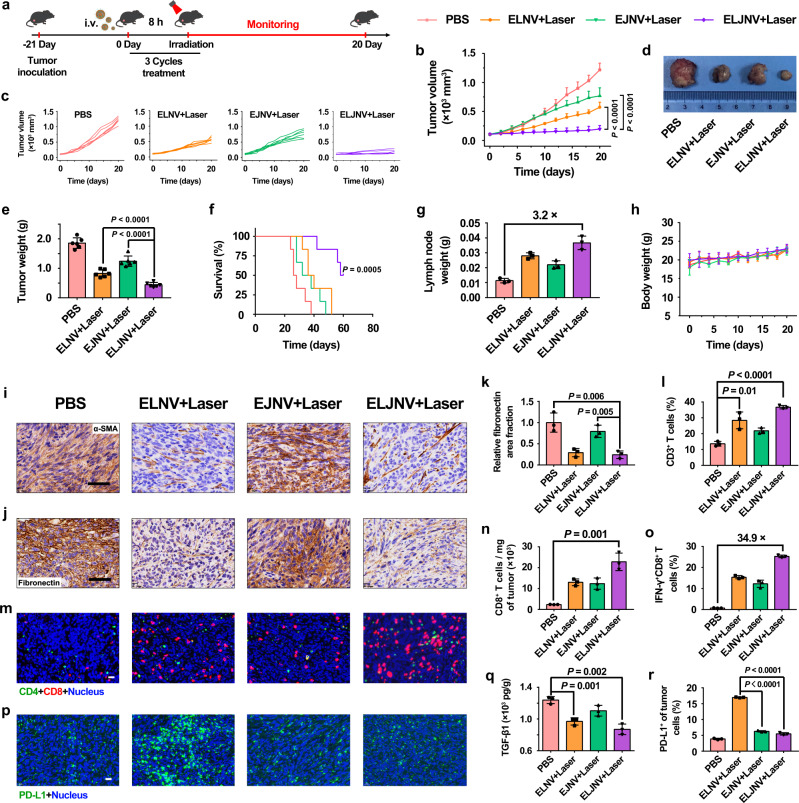
ELJNV nanovesicles overcame the intrinsic and inducible immune resistance in Panc02 tumors. **a** Treatment schedule of ELJNV-mediated combination immunotherapy in Panc02 tumor-bearing mice. **b**, **c** Tumor growth profiles of mice under different conditions (*n* = 6 mice, *P* = 3.17 × 10^−6^ and 5.09 × 10^−6^). **d** Typical photographs of excised tumors from different groups. **e** Average tumor weights of mice after the different treatments (*n* = 6 mice, *P* = 6.96 × 10^−5^ and 1.33 × 10^−6^). **f** Survival curves of tumor-bearing mice post different treatments. **g** The weight of lymph nodes in Panc02 tumor-bearing mice (*n* = 3 mice). **h** Averaged body weight of C57BL/6 mice under different conditions (*n* = 6 mice). **i** Immunohistochemical staining of the intratumoral α-SMA expression. Scale bar = 50 μm. **j** Immunohistochemical staining of the intratumoral fibronectin expression. Scale bar = 50 μm. **k** Semi-quantitative analysis of the relative fibronectin area fraction by Image J (*n* = 3 mice). **l** Flow cytometric examination of the tumor infiltrating CD3^+^ T lymphocytes in Panc02 tumor post different treatments (*n* = 3 mice, *P* = 9.69 × 10^−3^ and 2.75 × 10^−5^). **m** IF staining of CD4^+^ and CD8^+^ T lymphocytes in Panc02 tumor sections 3 days post the final treatments. Scale bar = 20 μm. **n** Normalized tumor infiltrating CD8^+^ T cells in Panc02 tumor-bearing mice (*n* = 3 mice). **o** Flow cytometric quantification of intratumoral infiltration of IFN-γ^+^CD8^+^ T cells in Panc02 tumor-bearing mice (*n* = 3 mice). **p** IF staining and **r** flow cytometry analysis of intratumoral PD-L1 expression in Panc02 tumors after different treatments (*n* = 3 mice, *P* = 2.04 × 10^−7^ and 9.31 × 10^−7^). Scale bar = 20 μm. **q** ELISA analysis of intratumoral TGF-β1 under different conditions (*n* = 3 mice). Error bars represent mean ± SD. *P* values in all panels except **f** derived from the Student’s *t*-test (two-tailed, two-sample unequal variance). In panel **f**, the Log-rank (Mantel–Cox) test was used. Source data are provided as a Source Data file.

Pancreatic cancer is one of the most lethal human cancers^42^. The abnormally abundant ECM is the most prominent pathologic feature of the pancreatic cancer microenvironment^43^, which similar to the tight cordon sanitaire, impedes the drug tumor penetration and reduces the efficacy of the chemotherapeutic agent. The antitumor performance of the ELJNV nanovesicles was therefore evaluated in vivo in the subcutaneous Panc02 murine pancreatic tumor model (Fig. 6a). Pancreatic cancer appeared to exhibit a better tumor inhibition effect compared with TNBC in the ELJNV + Laser group because the PD-L1 expression on the surface of the Panc02 tumor cells was more increased (Fig. 6b–e, Supplementary Fig. 44). H&E staining of tumor sections after treatment showed that the ELJNV + Laser group had the largest area of tumor necrosis (Supplementary Fig. 47a). Moreover, ELJNV + Laser significantly prolonged the survival of tumor-bearing mice without causing body weight loss (Fig. 6f, h). The immunohistochemical staining and flow cytometry assay of mouse tumor tissues at the end of the treatment illustrated that ELNV and ELJNV significantly decreased the expression of α-SMA, FAP, and fibronectin, thereby relieving the tumor fibrosis and promoting the tumor-infiltration of CD3^+^ T cells (Fig. 6i–l, and Supplementary Fig. 47b–d). These results were consistent with the stronger inhibitory effect of ELJNV on 4T1 breast tumors.

Furthermore, the in vivo activation of immune responses was caused by ELJNV-mediated immunogenic PDT^44^. Specifically, the ELJNV + Laser group showed a ~3.2-fold increase in LN weight compared to the PBS group (Fig. 6g). DC maturation was significantly enhanced by the ELJNV + Laser treatments, and the percentage of matured DCs reached 23.9 ± 1.3% (Supplementary Fig. 48a,b), indicating 8.0-times higher DC maturation ratio of the ELJNV + Laser group than that in the PBS group (3.0 ± 0.7%). Flow cytometry assay and IF staining demonstrated that the fraction of CD8^+^ T cells increased up to 59.8 ± 2.0% in the ELJNV + Laser group, 2.5-times higher than the PBS group (Fig. 6m, n, and Supplementary Fig. 48c). IFN-γ^+^CD8^+^ T cells demonstrated 34.9-times increase in the ELJNV + Laser group compared to the PBS group, which dramatically activated the antitumor immune response (Fig. 6o, and Supplementary Fig. 48d). The proportion of Tregs was significantly reduced from 17.4 ± 1.5% in the PBS group to 1.7 ± 0.2% in the ELJNV + Laser group and substantially improved the CD8^+^ T cell to Treg ratio compared with the PBS group 25.4-fold (Supplementary Fig. 48e–g). Interestingly, TGF-β1 secretion was also decreased in Panc02 tumors, which may be attributed to the effective relief of tumor fibrosis and the reduction of Tregs, as described previously (Fig. 6q). In addition, IF staining and flow cytometry results of PD-L1 expression demonstrated that the ELJNV + Laser group could also block the overexpression of PD-L1 caused by IFN-γ in situ by efficiently releasing JQ1, overcoming the inducible immune resistance, and relieving the immunosuppressive tumor microenvironment in pancreatic cancer (Fig. 6p, r, and Supplementary Fig. 48h).

**Fig. 7:**
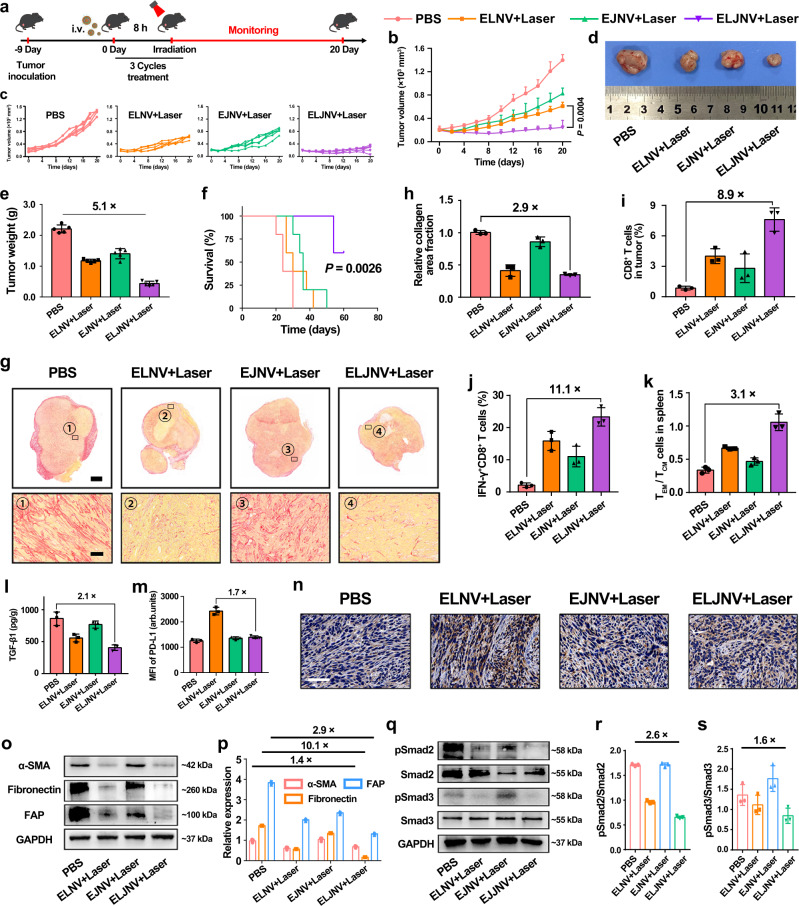
ELJNV nanovesicles migrated the intrinsic and inducible immune resistance in the KPC pancreatic tumor model. **a** Treatment schedule of ELJNV-mediated combination immunotherapy of KPC tumor. **b**, **c** Tumor growth curves of KPC tumor-bearing mice upon different treatments (*n* = 5 mice). **d** Representative photographs of KPC tumors collected at the end of the antitumor study. **e** Averaged tumor weights examined at the end of the antitumor study (*n* = 5 mice). **f** Survival curves of KPC tumor-bearing mice post different treatments. **g** Sirius red staining of tumor sections. Up panel (Scale bar = 2 mm), bottom panel (Scale bar = 50 μm). **h** Quantification of fibrosis by Sirius Red staining (*n* = 3 mice). **i** Flow cytometric quantification of intratumoral infiltration of CD8^+^ T cells in KPC tumor-bearing mice (*n* = 3 mice). **j** Flow cytometric quantification of intratumoral infiltration of IFN-γ^+^CD8^+^ T cells in KPC tumor-bearing mice (*n* = 3 mice). **k** Flow cytometry-determined central memory T lymphocytes (T_CM_) and the effector memory T lymphocytes (T_EM_) ratios in the spleens of KPC tumor-bearing C57BL/6 mice 21 days post-treatment (*n* = 3 mice). **l** ELISA analysis of intratumoral TGF-β1 secretion (*n* = 3 mice). **m** Flow cytometry analysis and **n** immunohistochemistry staining of intratumoral PD-L1 expression in KPC tumors after different treatments (*n* = 3 mice). **o** Western blot assay and **p** semi-quantification of α-SMA, FAP, and fibronectin expression in the tumor tissue at the end antitumor study (*n* = 3 mice). **q** Western blot assay and **r**, **s** semi-quantification of ELJNV-induced inhibition TGF-β/Smad signaling pathway in KPC tumors (*n* = 3 mice). Error bars represent mean ± SD. *P* values in all panels except **f** derived from the Student’s *t*-test (two-tailed, two-sample unequal variance). In panel **f**, the Log-rank (Mantel–Cox) test was used. Source data are provided as a Source Data file.

To validate the immune modulation effects and antitumor performance of ELJNV nanovesicles, a subcutaneous KPC pancreatic tumor model with a more immune-excluded microenvironment than Panc02 tumor was next established in C57BL/6 mouse (Fig. 7a). ELJNV + Laser noticeably extended the survival of the tumor-bearing mice and inhibited tumor growth (Fig. 7b–f). This favorable therapeutic performance was accompanied by a markable reduction of fibrosis and decreased collagen I expression in ELJNV-treated tumors (Fig. 7g, h). Compared to the control group, ELJNV + Laser increased the tumor-infiltrating CD8^+^ T cells and IFN-γ^+^CD8^+^ T cells by 8.9- and 11.1-fold and decreased Tregs by 5.6-fold, respectively (Fig. 7i, j and Supplementary Fig. 49a–f). These data indicated that ELJNV-based photo-immune and antifibrotic therapy dramatically elicited a protective immune response and relieved the immunosuppressive tumor microenvironment in KPC tumors. Furthermore, ELJNV + Laser increased the central memory T cells (T_CM_, CD45^+^CD3^+^CD8^+^CD44^+^CD62L^+^) to effector memory T cells (T_EM_, CD45^+^CD3^+^CD8^+^CD44^+^CD62L^−^) ratio by 3.1-fold in the spleens of KPC tumor-bearing mice (Fig. 7k and Supplementary Fig. 49g), suggesting that ELJNV markedly induced immune memory to prevent tumor relapse and distant metastasis^45^. The mechanistic studies showed that ELJNV treatment reduced TGF-β1 secretion and PD-L1 expression in KPC tumors (Fig. 7l–n and Supplementary Fig. 49h, i), and thus facilitated intratumor infiltration of CTLs (Supplementary Fig. 49j). WB analysis of the tumor lysate further demonstrated that ELJNV inhibited the TGF-β/Smad signaling pathway and downregulated their downstream protein expression (Fig. 7o–s).

To further demonstrate the potential of the nanovesicles for immunotherapy, we investigated their antitumor performance in subcutaneous B16-F10 melanoma and MC38 tumor models (both are well-defined hot tumors). LY-loaded ELJNV nanovesicles highly efficiently inhibited tumor growth of both B16-F10 melanoma and MC38 colorectal tumors (Supplementary Figs. 50, 51), suggesting their promising potential for highly efficient immunotherapy of a wide spectrum of solid tumors.

## Discussion

The immunosuppressive cytokine TGF-β1 is highly expressed in advanced tumors, which promotes tumor immune escape and distant metastasis^40,41^. In particular, TGF-β1 has been identified to induce dense desmoplasia surrounding the solid tumor as a physical barrier to restrict intratumoral infiltration of CTLs^46^. The infiltrating CTLs secreted IFN-γ could continue to upregulate PD-L1 on the tumor cells, as a potential negative feedback mechanism of tumor immune response, thereby generating the adaptive immune resistance^47,48^. To circumvent the intrinsic and adaptive immune resistance, an MMP-2-sheddable prodrug nanovesicle was thus engineered for tumor-specific co-delivery of multiple regimens including LY, PPa, and a ROS-liable JQ1 prodrug. The lipid-based nanovesicles were employed in this due to their unique potential for clinical translation including satisfying biosafety of the lipid ingredients, high drug loading capacity, and elongated blood circulation^49,50^. LY was selected to reverse CAFs activation and suppress tumor fibrosis by blocking the TGF-β signaling pathway, which promoted intratumoral infiltration of CLTs and overcame the intrinsic immune resistance. PPa-performed PDT to generate ROS and release JQ1 in a spatiotemporally tunable manner. Subsequently, JQ1 blocked IFN-γ-induced PD-L1 upregulation and overcame the adaptive immune resistance. Therefore, the nanovesicles exert an improved immunotherapy efficacy in a broad spectrum of solid tumors including 4T1 murine breast, Panc02/KPC pancreatic, B16-F10 melanoma, and MC38 colorectal tumors.

The combination of nanoparticle-based PDT and free-delivery LY was recently exploited to promote the intratumoral accumulation of nanoparticles by decreasing collagen deposition, alleviating solid stress, and decompressing tumor blood vessels^51^. However, systemic administration of free LY might cause severe side effects since TGF-β signaling is widely activated in normal tissues. In this study, LY was specifically delivered to the tumor with the nanovesicles for inactivating CAFs and eliciting an antitumor immune response, which is expected to avoid the reverse effect caused by non-specific TGF-β1 inhibition.

BRD4 is an important transcriptional and epigenetic regulator for oncogene transcription. BRD4 inhibitor JQ1 may regress tumor growth by other mechanisms in addition to PD-L1 inhibition. For instance, JQ1 can inhibit the cell cycle and induce apoptosis of tumor cells as reported in the tumor model of NUT midline carcinoma^52^. Second, JQ1 inhibits BET bromodomain–promoter interactions and subsequently reduces MYC transcript and protein expression, which results in G1 phase arrest and extensive apoptosis in a variety of leukemia and lymphoma cell lines^53^. Furthermore, JQ1-mediated BET inhibition could suppress the transcription of several downstream oncogenes (e.g., *BCL-2*, *C-Myc*, and *CDK-6*) to inhibit tumor growth^54^. The above evidence suggested that BRD4-targeted immunotherapy might benefit from multiple tumor inhibition mechanisms.

In summary, bidirectional regulation ELJNV nanovesicles were designed to simultaneously overcome intrinsic and inducible immune resistance. Compared to single-drug nanovesicles and free-delivery drugs, the LY and JQ1 co-delivery nanovesicles displayed several distinct advantages. First, the nanovesicles engineered for PPa and LY co-delivery can specifically accumulate and retain at the tumor site via passive tumor targeting effect and MMP-2-mediated cleavage of PEG corona, thus maximizing their therapeutic benefits while minimizing the side effects. Second, the nanovesicles can perform multi-imaging-guided PDT to elicit the immunogenicity of tumor cells, which is promising for precise immunotherapy of cancer. LY delivered with the nanovesicles can specifically inactivate CAFs and relieve the physical barrier of the immune-excluded tumors, which facilitates intratumoral infiltration of T lymphocytes. It was found that the combination of LY and JQ1 with PDT could elicit immunogenicity of tumor cells, reduce tumor fibrosis, and reduce PD-L1 expression on tumor cells, which synergistically facilitates CTL infiltration and improves the effectiveness of photoimmunotherapy. Furthermore, the combination of antifibrotic therapies and immune-enhancing therapies might pave the way for promoting immunotherapy of a broad spectrum of the tumor, including both the IETs and hot tumors.

## Methods

### Materials

Pyropheophorbide a (PPa) was purchased from Dibai Chem Tech Co., Ltd (Shanghai, China). JQ1 and LY2157299 (LY) were purchased from Selleck Chem Co., Ltd (Shanghai, China). Matrix metalloproteinase-2 (MMP-2) and L-ascorbic acid were purchased from Sigma-Aldrich (Shanghai, China). Fmoc-protected heptapeptide Gly-Pro-Leu-Gly-Leu-Ala-Gly (Fmoc-GPLGLAG) was synthesized by GL Biochem. Co., Ltd (Shanghai, China). Methoxypolyethylene glycol amine (mPEG_5k_-NH_2_) was purchased from Seebio Biotech. Co., Ltd (Shanghai, China). N-(3-(dimethylamino)-propyl)-N-ethylcarbodiimide hydrochloride (EDCI), 1-hydroxybenzotriazole anhydrous (HOBT), 4-dimethylaminopyridine (DMAP), triethylamine (TEA), and trifluoroacetic acid (TFA), N,N-Diisopropylethylamine (DIEA), triethylene glycol, anhydrous dichloromethane (DCM), gadolinium(III) chloride hexahydrate anhydrous, N,N-dimethylformamide (DMF) and anhydrous dimethyl sulfoxide (DMSO) were purchased from J&K Scientific Ltd (Beijing, China). 2′, 7′-dichlorofluorescein diacetate (DCFH-DA) was purchased from Sigma-Aldrich (Shanghai, China). Singlet oxygen sensor green (SOSG) and cell counting kit-8 were purchased from Dalian Meilun Biotech CO., Ltd (Dalian, China). 1-palmitoyl-2-hydroxy-sn-glycero-3-phosphocholine (p-lysoPC), 1,2-dipalmitoyl-sn-glycero-3-phosphocholine (DPPC), and 1,2-dioctadecanoyl-sn-glycero-3-phosphocholine (DSPC) were obtained from Advanced Vehicle Technology Pharmaceutical Co., Ltd (Shanghai, China). RPMI 1640 cell medium, 0.25% trypsin-EDTA (Phenol Red), penicillin/streptomycin solution, pre-stained rainbow protein marker (10–190 kDa), Bicinchoninic Acid (BCA) protein quantification/concentration determination kit, 4′, 6-diamidino-2-phenylindole dihydrochloride (DAPI), PBS buffer solution and TBST buffer solution (10×) were purchased from Meilun Biotech Co., Ltd (Dalian, China). Lysotracker green was purchased from Life Technologies (Shanghai, China). The mouse lymphocyte separation medium was obtained from Dakewe Biotech (China). Antibodies against GAPDH, calreticulin and high mobility group proteins B1 (HMGB1) were all ordered from Abcam (UK). IFN-γ, GM-CSF, IL-4, and ELISA kits of TGF-β1 and IFN-γ (EMC101g.96) were all purchased from Neobioscicence Technology Co., Ltd (Shenzhen, China).

### Cell lines and animals

Panc02 murine pancreatic tumor cells, 4T1 murine breast cancer cells, B16-F10 murine melanoma tumor cells, and MC38 murine colorectal tumor cells were all obtained from the cell bank of the Chinese Academy of Sciences (Shanghai, China). NIH3T3 cells were purchased from Shanghai Bogoo Biotechnology. Co., Ltd. KPC pancreatic tumor cells were kindly provided by Dr. Y. Huang from SIMM of CAS, China. KPC cells were obtained from the primary KPC tumors in Pdx-Cre; KrasG12D/+; Trp53R172H/+ mice. Panc02, B16-F10, MC38, KPC, and NIH3T3 cells were all maintained in Dulbecco’s modified Eagle’s medium (DMEM, Gibco) supplemented with 10% FBS, 2 mM of L-glutamine, 1 mM of sodium pyruvate, 0.1 mM of non-essential amino acids, and 1% Penicillin-Streptomycin at 37 °C in 5% CO_2_. All experiments were performed in the logarithmic phase of cell growth. 4T1 tumor cells were cultured in RPMI 1640 cell medium with 10% FBS, 0.11 g/L of sodium pyruvate, and 2.5 g/L of glucose, and incubated at 37 °C under a humidified atmosphere containing 5% of CO_2_. The primary CAFs were obtained from Panc02 tumor xenograft.

For in vivo studies, BALB/c mice (female, 6~8 weeks, 18~20 g), C57BL/6 (female, 6~8 weeks, 18~20 g), and BALB/c nude mice (female, 6~8 weeks, 18~20 g) were obtained from Shanghai Experimental Animal Center (Shanghai, China). All mice were kept under the pathogen-free condition and used by following the animal experimental guidelines approved by the Institute of Animal Care and Use Committee, Shanghai Institute of Materia Medica, Chinese Academy of Sciences. Animals were housed under SPF conditions and maintained at ~25 °C in a humidity-controlled environment with a 12 h light/dark cycle, with free access to standard food and water.

### Fabrication and characterization of the ELNV and ELJNV nanovesicles

To prepare the ELNV nanovesicles, DPPC, DSPC, PPa-GPLGLAG-PEG_5k_, and LY were mixed with the mass ratio of 3: 0.78: 1.15: 0.4 and dissolved by the mixed solvent of chloroform and methanol (v/v = 1:9). The mixture was vacuum dried to form lipid film, which was rehydrated in PBS (pH 7.4) for 5 min at 50 °C and squeezed through 200 and 100 nm polycarbonate filters to get uniform ELNV nanovesicles. Meanwhile, LNV (enzyme-insensitive nanovesicles, replaced PPa-GPLGLAG-PEG_5k_ with PPa-PEG_5k_) and ENV (without LY) nanovesicles were prepared by the same method as above.

To fabricate the EJNV nanovesicles, 1,2-dioctadecanoyl-sn-glycero-3-phosphocholine (DSPC) used above was replaced by the equal quality of JTP and dissolved by the same mixed solvent of chloroform and methanol (volume/volume [v/v] = 1:9). Other preparation steps are the same as ELNV nanovesicles. Meanwhile, LJNV (JNV with LY) and ELJNV (EJNV with LY) nanovesicles were prepared by the same method as above. The size distribution and morphology of the nanovesicles were examined by DLS (Zetasizer Nano ZS90, Malvern Instrument, UK) and TEM measurements (Talos L120C, USA, 120 kV), respectively. The LY loading ratio (DL%) and encapsulation efficiency (EE%) were measured using HPLC. Laser-induced ROS generation in PBS or 10% SDS was evaluated by using SOSG as a fluorescence ROS probe (excitation/emission [Ex/Em] = 504/525 nm).

### Biodistributions of nanovesicles in 4T1 tumor-bearing mice in vivo

4T1 breast tumor model was generated by subcutaneously (s.c.) injecting 3 × 10^6^ 4T1 tumor cells into the right fat pad of BALB/c mice. LNV or ELNV (PPa concentration, 3 mg/kg) was i.v. injected when the tumor volume reached 200 mm^3^ (*n* = 3). The fluorescence images in vivo were taken by IVIS imaging system. For immunofluorescent staining ex vivo, tumor tissues were fixed in 4% paraformaldehyde for 24 h. 20% sucrose solution was used to dehydrate tissues for 24 h, and 30% sucrose solution was used to further dehydrate tissues for another 36 h. Afterward, the tumor tissues were embedded in O.C.T. (Optimal Cutting Temperature) and sectioned into 5 μm slices (Leica CM1950, Leica Biosystems Inc., USA). To visualize the tumor blood vessels, tumors were stained with anti-CD31 antibody (Abcam, ab281583, 1:50) and Alexa Fluor 488 conjugated secondary antibody (Abcam, ab150077, 1:500).

To quantitative biodistribution of PPa and LY, free PPa/LY, LNV, and ELNV nanovesicles were i.v. injected into tumor-bearing BALB/c mice at PPa dose of 5 mg/kg and LY dose of 20 mg/kg (*n* = 3). At 1, 2, 4, 6, 12, 24 h post-injection, the major organs and tumors were harvested and then the PPa and LY contents were analyzed by HPLC and fluorescent photospectrometer, respectively.

### WB assay and IF staining of α-SMA expression in vitro

To evaluate that TGF-β1 activates the fibroblasts through the TGF-β/Smad signaling pathway, NIH3T3 cells were incubated in 6-well plates at a density of 3 × 10^5^ cells/well for 24 h. The cells were treated with gradient concentrations of TGF-β1 for 24 h. The cells were collected, and the expressions of α-SMA (ab124964, 1:10000), Smad2 (ab33875, 1:1000), pSmad2 (ab280888, 1:1000), Smad3 (ab84177, 1:500), and pSmad3 (ab52903, 1:2000) were analyzed by WB (Image Lab 3.0).

To explore LY-attenuated CAFs activation via inhibiting the TGF-β/Smad signaling pathway, the primary CAFs collected from Panc02 tumor xenograft or NIH3T3 cells were incubated in 6-well plates at a density of 3 × 10^5^ cells/well for 24 h. The cells were pre-treated with indicated concentrations of LY for 12 h followed by the incubation of 5 ng/mL TGF-β1 for another 24 h. Then α-SMA, fibronectin, FAP, Smad2, pSmad2, Smad3, and pSmad3 expression were analyzed by WB. Similarly, we further studied the ELNV (LY concentration of 5 μM) nanovesicles attenuate NIH3T3 activation and their underlying impacts on TGF-β/Smad signaling pathway. The CAFs-related proteins in NIH3T3 (e.g., α-SMA, fibronectin, FAP), and the key proteins in the TGF-β signaling pathway (e.g., Smad2, pSmad2, Smad3, pSmad3) were analyzed by WB. To visualize the α-SMA marker in NIH3T3 cells, the cells were treated with 5 ng/mL TGF-β1 and 5 ng/mL TGF-β1 plus 5 μM LY by the previously described method^16^, then the cells were stained with anti-alpha smooth muscle actin antibody (Abcam, ab124964, 1:250) and Alexa Fluor 488 conjugated second antibody (Abcam, ab150077, 1:500). CLSM was performed to investigate α-SMA expression ex vivo.

### Masson trichrome and immunohistochemical staining of the tumor fibrosis ex vivo

To evaluate whether LY inhibits tumor fibrosis and promotes lymphocyte infiltration in the tumor, 4T1 tumor-bearing mice were intratumor injected with LY (0.75 or 1.5 mg/kg) every other day. After one week, all mice were sacrificed and the tumors were sampled, Masson trichrome staining and immunohistochemical staining with anti-CD3 antibody (Abcam, ab135372, 1:150) were subsequently performed on the tumor sections (*n* = 3), finally measured by KF-PRO-120 digital pathology slide scanner (KFBIO Co., Ltd.). The depth of penetration into tumor sections of CD3^+^ T cells was estimated by ZEISS software. For evaluation of the CD3^+^ T cells (CD45^+^CD3^+^) and CD8^+^ T cells (CD45^+^CD3^+^CD8^+^), the T lymphocytes were stained with anti-CD45-APC (BD, 559864, 1:100), anti-CD3-PerCP-Cy5.5 (BD, 551163, 1:100) antibodies according to the manufacturer’s protocols, and further examined with flow cytometry.

### Cytotoxicity and phototoxicity of ELNV nanovesicles in vitro

CCK-8 assay was used to detect the cytotoxicity of the nanovesicles. 4T1 tumor cells and 3T3 fibroblast cells were incubated in 96-well plates (5 × 10^3^ cells/well) for 24 h. The cells were then treated with ELNV nanovesicles at 5, 15, 37.5, 75, 150, 300, and 600 μg/mL for 24 h. To detect the cytotoxicity of ELJNV nanovesicles, 4T1 cells were incubated in 96-well plates (5 × 10^3^ cells/well) for 24 h. Then the cells were treated with ELJNV nanovesicles for 24 h at PPa concentrations of 0.5, 1, 2, 5, 10, or 20 μM. CCK-8 assay was performed to explore the phototoxicity of free PPa and ELNV in vitro. 4T1 cells were incubated in 96-well plates for 24 h at a density of 5000 cells/well. After incubating with different concentrations of free PPa or PPa in ELNV nanovesicles (i.e., 1.25, 2.5, and 5 μM) for 12 h, the cells were washed and illuminated with 671 nm laser for 30 s at photodensity of 50, 150, 300 or 500 mW/cm^2^. Afterward, the cells were collected for another 24 h and finally detected by CCK-8 assay.

### Immunogenic cell death in vitro and in vivo

The ICD effect of ENV and ELNV were detected by analyzing CRT exposure and nuclear HMGB1 efflux in 4T1 cells in vitro. 4T1 cells were treated with PBS, free PPa + Laser, ELNV, ENV + Laser, ENV + LY + Laser, and ELNV + Laser at an identical PPa concentration of 5.0 μM for 12 h (*n* = 3). The cells were then irradiated with a 671 nm laser (150 mW/cm^2^) for 30 s, and continually incubated for 4 h at 37 °C. Afterward, the cells were stained with an anti-CRT primary antibody (Abcam, ab92516, 1:50 for flow cytometry and 1:300 for IF) and Alexa488-conjugated secondary antibody (Abcam, ab150077, 1:500) for 30 min. The cells were then analyzed by flow cytometry and CLSM. For detecting HMGB1 efflux by CLSM, the cells were permeabilized in 0.1% Triton X-100 for 5 min and then blocked in 5% FBS for 1 h at RT before staining with anti-HMGB1 antibody (Abcam, ab18256, 1 µg/mL).

To examine CRT exposure in vivo, subcutaneous 4T1 tumor-bearing BALB/c mice were divided into four groups when tumor volume reached 100 mm^3^. The mice were then treated with PBS, ELNV, ENV + Laser, or ELNV + Laser. All the formulations were i.v. injected at an equal LY dose of 20 mg/kg and a PPa dose of 5 mg/kg. At 8 h post-treatment, the tumor tissues were irradiated with a 671 nm laser for 5 min (400 mW/cm^2^). The tumors were collected 12 h post-irradiation, and the tumor tissue slices were immunostained with anti-CRT primary antibody (Abcam, ab92516, 1:300) and Alexa488-conjugated monoclonal secondary antibody (Abcam, ab150077, 1:500) and measured by CLSM.

### DC maturation in vitro

To explore PDT-induced DC maturation in vitro, the bone marrow dendritic cells (BMDCs) were obtained from BALB/c mice. The 4T1 cells were pretreated by free PPa and the nanovesicles at the identical PPa concentration of 5 μM for 24 h, followed by washing the cells with fresh cell culture medium three times and immediately irradiated with 150 mW/cm^2^ 671 nm laser for 1 min. After continuing to culture 4T1 cells for another 4 h, the BMDCs were co-incubated with the above tumor cells for 24 h. The cells were then stained with anti-CD11c-FITC (BD, 557400, 1:100), anti-CD80-PE (BD, 553769, 1:100), and anti-CD86-APC (BD, 558703, 1:100) antibodies for 1 h before flow cytometry.

### Magnetic resonance (MRI) and photoacoustic imaging (PAI)

MRI was performed with 9.4 T BioSpec MRI (Bruker, Germany). The *T*_1_ MR map was generated with a 96-well plate containing 100 μL aliquots of nanovesicles with MMP-2 (40 μg/mL) suspension, ranging in Gd^3+^ concentration from 0 to 0.2 mM. For MRI in vivo, 4T1 tumor-bearing nude mice were i.v. injected with 100 μL of LNV or ELNV nanovesicles at an identical PPa dose of 5.0 mg/kg. After 8 h, the mice were anesthetized by isoflurane, followed by MRI. The instrument parameters were set as follows: TE = 8.5 ms; TR = 1000.0 ms; FOV (field of view) = 3.00 cm; slice thickness = 1.00 mm; matrix size = 256 × 256.

PA imaging in vitro and in vivo was conducted on a multispectral optoacoustic tomography (MSOT) small-animal scanner (InVision 256-TF, iThera Medical, Germany) at an excitation wavelength of 680 nm. Polyethylene tubes (i.d. ∼3.0 mm) were held submerged in an ultrasonic coupling agent, and the ultrasound transducer was placed across them. The nanovesicle solution was added to the tubes, and the PA measurements were conducted at three locations across the tubes. The average PA signal intensity for each group was then plotted against the nanovesicle concentration to give the relative PA signal intensities. For MSOT imaging in vivo, the female nude mice bearing subcutaneous 4T1 cells tumors were anesthetized with 2% isoflurane in oxygen and then located in the prone position. Up i.v. injection of LNV and ELNV at an identical PPa dose of 5.0 mg/kg, the PA images of the tumor area were monitored via an MSOT system.

### IFN-γ-induced programmed death ligand 1 (PD-L1) expression in vitro

To investigate the effect of JQ1 on PD-L1 expression in vitro, 4T1 and Panc02 cells were incubated in six-well plates at a density of 2.5 × 10^5^ cells per well for 24 h. Then the cells were treated with JQ1, JTP, JP, and IFN-γ at the desired concentrations (e.g., 0.2 or 1 μM of JQ1 and 100 ng/mL of IFN-γ) for 24 h. Afterward, the cells were treated with JQ1, JTP, and JP for another 24 h at an identical JQ1 concentration of 200 nM in 4T1 cells or 1000 nM in Panc02 cells. To investigate the effect of EJNV on PD-L1 expression in vitro, the cells were pre-treated with JQ1 or EJNV, as well as, 100 ng/mL of IFN-γ for 24 h, and then incubated with JQ1 or EJNV for another 24 h. JP and JTP in DMSO or EJNV in PBS solution was irradiated with a 671 nm laser (150 mW/cm^2^) for 5 min to release JQ1-SH in the presence of PEG_5k_-PPa (5 μM PPa) before addition into the cell culture medium. Finally, the cells were stained with anti-CD274-APC antibody (BD, 564715, 1:100) and examined by flow cytometry.

### Anti-tumor performance in KPC pancreatic tumor, B16-F10, and MC38 tumor models

A subcutaneous KPC tumor model was established by subcutaneously injecting 1 × 10^7^ KPC tumor cells into the left leg of C57BL/6 mice. The tumor-bearing mice were randomly grouped (*n* = 10) when the tumor volume reached around 150 mm^3^ and treated with PBS, ELNV + Laser, EJNV + Laser, or ELJNV + Laser, at an identical PPa, LY, and JQ1 dose of 5.0, 20 and 15 mg/kg, respectively. The tumors in the ELNV + Laser, EJNV + Laser, and ELJNV + Laser-injected groups were irradiated with a 671 nm laser for 1 min (400 mW/cm^2^) at 8 h post i.v. injection of the nanovesicles. The treatment was repeated triplicately at a time interval of 3 days. Body weight and tumor volume were monitored every 2 days over a whole period of 20 days. The tumor tissues were harvested at the end of the antitumor study and stained with sirius red to evaluate CAFs inactivation in vivo.

To examine nanovesicle-induced intratumoral TGF-β1 secretion, the KPC tumors were collected, digested, and examined by ELISA kits (*n* = 3). α-SMA, FAP, fibronectin, and (p)-Smad2/3 expressions were examined by western-blot assay and semi-quantified with ImageJ software (*n* = 3).

To evaluate whether ELJNV promotes intratumoral infiltration of T lymphocytes IF staining of the KPC tumor sections was performed at the end antitumor study (*n* = 3). The tumor-infiltrating CD3^+^ T cells, CD8^+^ T cells, CD4^+^ T cells, Tregs, and IFN-γ^+^CD8^+^ T cells were analyzed by flow cytometry. Anti-CD45-FITC (BD, 553079), anti-CD3-PerCP-Cy5.5 (BioLegend, 100218), anti-CD4-APC/Cy7 (BD, 552051), anti-CD8-PE (BD, 553032), anti-IFN-γ-APC (BD, 554413), anti-CD25-APC (Invitrogen, 17-0257-42), anti-Foxp3-PE (Invitrogen, 72-5775-40) were used.

To evaluate central memory T lymphocytes (CD45^+^CD3^+^CD8^+^CD44^+^CD62L^+^) and effector memory T lymphocytes (CD45^+^CD3^+^CD8^+^CD44^+^CD62L^−^) in vivo, the spleens of the KPC tumor-bearing mice were harvested 21-days post the last treatment and grounded to the single cell suspension, and then stained with anti-CD45-PE (Multi Sciences, F2104502, 1:100), anti-CD8-PE/Texas red (Abcam, ab25294, 1:100), anti-CD44-APC (BD, 559250, 1:100) and anti-CD62L-FITC (BioLegend, 104405, 1:100) antibodies were used (*n* = 3).

### Pharmacokinetics of prodrug nanovesicles

Free LY/PPa, LNV, and ELNV nanovesicles were intravenously injected into healthy BALB/c female mice at a PPa dose of 5 mg/kg and an LY dose of 20 mg/kg (*n* = 3). Blood samples were collected at 2 min, 10 min, 30 min, 1 h, 2 h, 6 h, 8 h, 12 h, 24 h, and 36 h post-injection. Afterward, the content of PPa and LY in the blood was measured by HPLC.

### Antitumor effect and biosafety assay in vivo

The subcutaneous 4T1 breast tumor model was generated by subcutaneously (s.c.) injecting 3 × 10^6^ 4T1 tumor cells into the right fat pad of BALB/c mice. The pancreatic tumor model was established by s.c. injecting 1 × 10^7^ Panc02 cells on the leg of C57BL/6 mice. 4T1 tumor-bearing mice were randomly grouped (*n* = 12) when the tumor volume reached 150 mm^3^ and treated with PBS, ELNV, ENV + Laser, ENV + LY + Laser, and ELNV + Laser, respectively, at a PPa and LY dose of 5.0 and 20 mg/kg. The tumors in the ENV + Laser, ENV + LY + Laser, and ELNV + Laser-injected groups were locally irradiated with a 671 nm laser for 1 min (400 mW/cm^2^) 8 h post-injection. The treatment was repeated three times at a time interval of 3 days. Body weight and tumor volume were monitored every 2 days (*n* = 6). The mice were sacrificed, and tumors were harvested, weighed and photographed at the end of the antitumor study. The major organs and tumors were analyzed with H&E staining. Lung metastasis was quantified by counting the tumor foci or lung weights.

To demonstrate the efficiency of LY and JQ1 prodrug co-delivery nanovesicles, the 4T1 tumor-bearing BALB/c mice or Panc02 tumor-bearing C57BL/6 mice were randomly grouped (*n* = 12) when the tumor volume reached 150 mm^3^ and treated with PBS, ELNV + Laser, EJNV + Laser, or ELJNV + Laser, respectively, at a PPa, LY, and JQ1 dose of 5.0, 20 and 15 mg/kg. The same treatment was performed 3-weeks after Panc02 cell inoculation on C57BL/6 mice. The mice were euthanized according to the animal ethics of our institute and animal welfare when the tumor volume reached 1500 mm^3^. The tumor volume was calculated by the formula:

$${{{{{\rm{V}}}}}}=({{{{{\rm{L}}}}}}\times {{{{{\rm{W}}}}}}\times {{{{{\rm{W}}}}}})/2,\,({{{{{\rm{L}}}}}},\,{{{{{\rm{the}}}}}}\,{{{{{\rm{longest}}}}}}\,{{{{{\rm{dimension}}}}}};\,{{{{{\rm{W}}}}}},\,{{{{{\rm{the}}}}}}\,{{{{{\rm{shortest}}}}}}\,{{{{{\rm{dimension}}}}}})$$

### Tumor fibrosis and immune response evaluations in vivo

To determine the tumor fibrosis and intratumor-infiltrating T lymphocytes in vivo after being treated with the nanovesicles, the subcutaneous 4T1 breast cancer model and Panc02 pancreatic cancer model were generated and after the tumors were grown up to around 100 mm^3^, the mice were randomly divided into 5 or 4 groups (*n* = 3) and treated with PBS, ELNV, ENV + Laser, ENV + LY + Laser and ELNV + Laser; PBS, ELNV, EJNV + Laser and ELJNV + Laser at an identical PPa dose of 5.0 mg/kg and LY dose of 20.0 mg/kg, 8 h later, irradiated with a 671 nm laser at 400 mW/cm^2^ for 1 min every 3 days and treatment 2 times, respectively. Tumors and tumor-draining LNs were harvested and weighed to obtain lymphocytes on the 3rd-day post the last treatment. To evaluate whether the nanovesicles inhibit tumor fibrosis and promote lymphocyte infiltration in the tumor IF staining with anti-collagen I antibody (Abcam, ab270993, 1:500) and anti-CD3 antibody (Abcam, ab135372, 1:150) was performed on the tumor sections (n = 3). In addition, anti-Smad3 (phospho S423 + S425) antibody (Abcam, ab52903, 1:100), anti-alpha smooth muscle actin antibody (Abcam, ab124964, 1:1000), anti-fibronectin antibody (Abcam, ab2413, 1:100), and anti-Fibroblast activation protein, alpha antibody (Abcam, ab218164, 1:200) were also used to evaluate that LY reversed the activation of CAFs through TGF-β1 signaling pathway in vivo and measured by KF-PRO-120 digital pathology slide scanner (KFBIO Co., Ltd.) or PANNORAMIC MIDI II (3DHISTECH Ltd.). For DC maturation analysis, the lymph nodes were ground into single-cell suspension and stained with anti-CD11c-FITC (BD, 557400, 1:100), anti-CD80-PE (BD, 553769, 1:100), and anti-CD86-APC (BD, 558703, 1:100) antibodies, further examined with flow cytometry. To examine PD-L1 expression on the surface of the tumor cells (CD45^−^CD274^+^), the cells were stained with anti-CD45-APC (BD, 559864, 1:100), and anti-CD274-PE (BD, 558091, 1:100) antibodies, and further examined with flow cytometry.

To evaluate tumor-infiltrating CD3^+^ T cells (CD45^+^CD3^+^), CD8^+^ T cells (CD45^+^CD3^+^CD8^+^), CD4^+^ T cells (CD45^+^CD3^+^CD4^+^), Tregs (CD3^+^CD4^+^ Foxp3^+^) and IFN-γ^+^CD8^+^ T cells (CD45^+^CD3^+^CD8^+^IFN-γ^+^), the T lymphocytes were stained with anti-CD45-APC (BD, 559864, 1:100), anti-CD3-PerCP-Cy5.5 (BD, 551163, 1:100), anti-CD4-FITC (BD, 553046, 1:100), anti-CD8-PE (BD, 553033, 1:100), anti-Foxp3-PE (BD, 560414, 1:100), and anti-IFN-γ-FITC (BD, 554411, 1:100) antibodies according to the manufacturer’s protocols, further examining with flow cytometry. IF staining was performed to visualize CD4^+^ and CD8^+^ T cells and PD-L1 expression in pancreatic tumors.

### IFN-γ and TGF-β1 secretion in the tumor tissues

To probe the nanovesicle-induced IFN-γ and TGF-β1 secretion in tumors, the 4T1 tumor-bearing BALB/c mice or Panc02 tumor-bearing C57BL/6 mice (*n* = 3) were treated with i.v. injection of PBS, ELNV, ENV + Laser, ENV + LY + Laser, ELNV + Laser, EJNV + Laser, or ELJNV + Laser at an identical dose of PPa (5 mg/kg), LY (20 mg/kg) and JQ1 (15 mg/kg). The treatment was repeated two times every three days. Then the tumors were collected on the 3rd day post the final treatment and examined by ELISA kits. The exploration of TGF-β1 secretion in the tumors of C57BL/6 mice was identical to the above-mentioned experiments. Briefly, the Panc02 tumor-bearing C57BL/6 mice (*n* = 3) were treated with i.v. injection of PBS, ELNV + Laser, EJNV + Laser, or ELJNV + Laser at an identical dose of PPa (5 mg/kg), LY (20 mg/kg), and JQ1 (15 mg/kg). The treatment was repeated two times every three days. Then the tumors were collected on the 3rd day post the final treatment and examined by TGF-β1 ELISA kits.

### Statistical analysis

All data were given as Mean ± SD. GraphPad Prism software 8.0 was used for the statistical analyses. Two-tailed Student’s *t*-test was used for the statistical comparison between the two groups. A two-sided log-rank (Mantel–Cox) test was used for the statistical comparison of the survival study. The pharmacokinetic parameters were calculated using the DAS (Data Analysis System) software (version 3.0).

### Reporting summary

Further information on research design is available in the Nature Portfolio Reporting Summary linked to this article.

## Supplementary information

Supplementary Information

Reporting Summary

## Data Availability

The data of Fig. 2a, c and Supplementary Figs. 1, 3–8 were downloaded directly from the online database TNMplot (https://tnmplot.com/analysis/). The data of Fig. 2b and supplementary Fig. 16 were downloaded directly from the online database GEPIA (http://gepia.cancer-pku.cn/). The data of Fig. 2d was downloaded directly from the online database TIMER (https://cistrome.shinyapps.io/timer/). The data of Fig. 2e and Supplementary Figs. 2, 9–10 were downloaded directly from the online database TIMER2.0 (http://timer.comp-genomics.org/). The source data underlying Figs. 2–7, Supplementary Figs. 3–8, 11–13, 19–34, 41–51, Supplementary Tables 2–5 and Western blot are provided with this paper. The authors declare that the data supporting the findings of this study are available within the Article, Supplementary Information and Source data. Source data are provided with this paper.

## Source data

Source Data
