# Supplementary material for: Nanovesicles loaded with a TGF-β receptor 1 inhibitor overcome immune resistance to potentiate cancer immunotherapy
Source: Nat Commun. 2023 Jun 16;14:3593. doi: 10.1038/s41467-023-39035-x (PMC10275881; doi:10.1038/s41467-023-39035-x)
Supplement: Supplementary file 1 — Supplementary Information [file 41467_2023_39035_MOESM1_ESM.pdf]

Supplementary Information for

Nanovesicles loaded with a TGF-β receptor 1 inhibitor overcome immune resistance to potentiate cancer immunotherapy

Mengxue Zhou[1,†], Jiaxin Wang[1,2,†], Jiaxing Pan[1,†], Hui Wang[2,†], Lujia Huang[1], Bo Hou[1], Yi Lai[1], Fengyang Wang[3], Qingxiang Guan[4], Feng Wang[5], Zhiai Xu[6], Haijun Yu[1,*]

[1] Center of Pharmaceutics, Shanghai Institute of Materia Medica, Chinese Academy of Sciences, Shanghai 201203, China;

[2] College of Chemistry and Chemical Engineering, Inner Mongolia University, Huhhot, 010021, China;

[3] Shanghai Tenth People's Hospital, Tongji University School of Medicine, Shanghai 200072, China;

[4] School of Pharmacy, Jilin University, Changchun, 130021, China;

[5] Department of Gastroenterology, Huadong Hospital, Shanghai Medical College, Fudan University, Shanghai 200040, China;

[6] School of Chemistry and Molecular Engineering, East China Normal University, Shanghai 200241, China.

[*] Corresponding authors:

Prof. H. Yu (hjyu@simm.ac.cn), Center of Pharmaceutics, Shanghai Institute of Materia Medica, Chinese Academy of Sciences, Shanghai 201203, China.

† These authors contributed equally to this work.

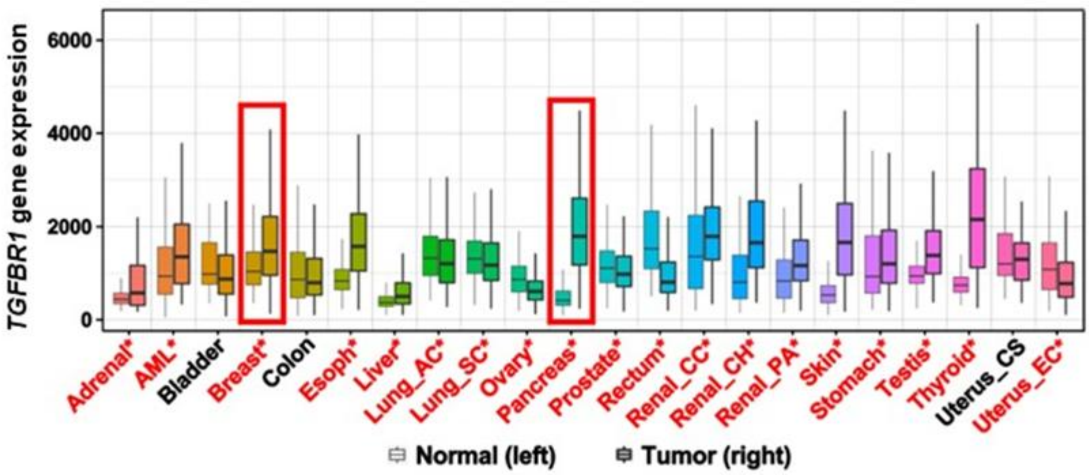

Supplementary Figure 1. *TGFBR1* gene expression in different normal and tumor tissues (n = 15648 normal, 40442 tumor samples). Significant differences by a two-sided Mann–Whitney U test are marked with red color (* $p < 0.01$). Plot was downloaded from the online database TNMplot (https://tnmplot.com/analysis/). Boxplot represents the median, interquartile range, upper whisker, maximum and minimum. Copyright ©: Department of Bioinformatics, Semmelweis University 2021-2023[2].

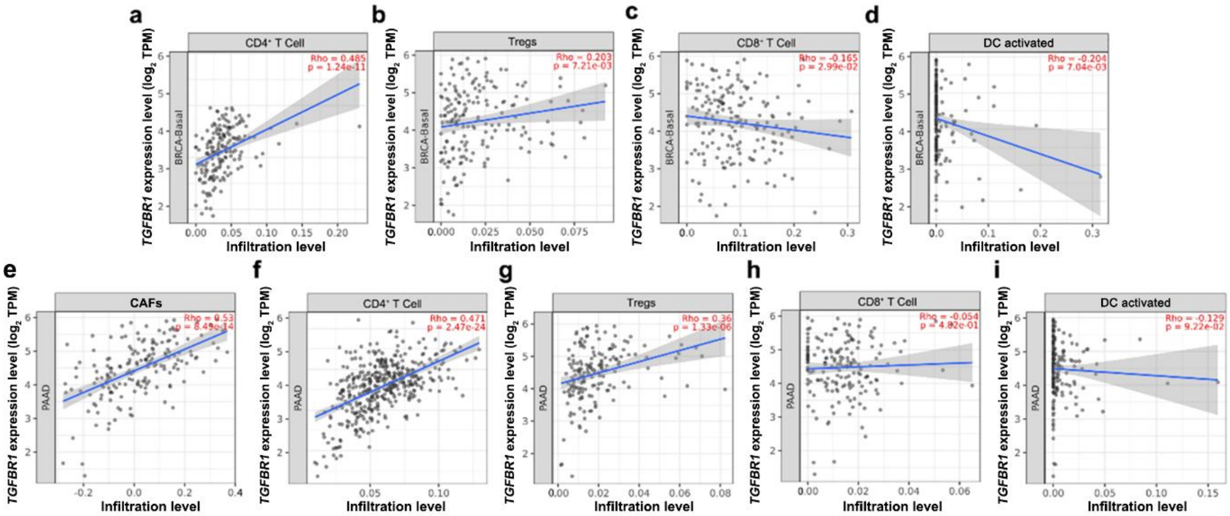

Supplementary Figure 2. Correlation between *TGFBR1* expression with survival and immune cell infiltration level in the TIMER database. (**a-d**) The correlation between *TGFBR1* expression and intratumoral infiltration level of (**a**) CD4^{+} T cells, (**b**) Tregs, (**c**) CD8^{+} T cells, and (**d**) myeloid DC activated in breast invasive carcinoma (BRCA-Basal) patients (n=191). (**e-i**) The correlation between *TGFBR1* expression and intratumoral infiltration level of (**e**) cancer-associated fibroblasts (CAFs), (**f**) CD4^{+} T cell, (**g**) Tregs, (**h**) CD8^{+} T cell, and (**i**) myeloid DC activated in pancreatic adenocarcinoma patients (n=179). Shaded error bands depict the standard error. Spearman test was used to determine the correlation coefficients.

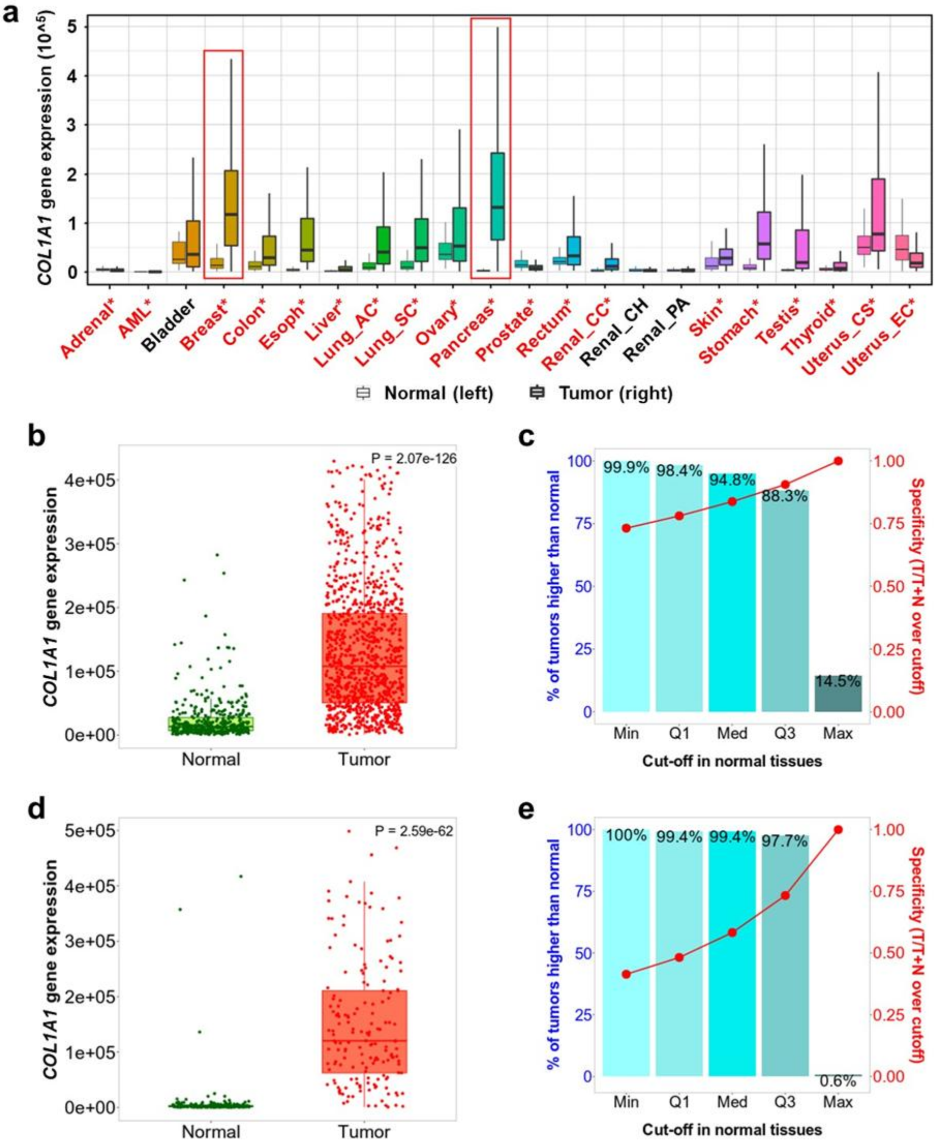

Supplementary Figure 3. *COL1A1* gene expression in normal and tumor tissues. (**a**) *COL1A1* gene expression in different normal and tumor tissues (n = 15648 normal, 40442 tumor samples). Significant differences by a two-sided Mann–Whitney U test are marked with red color (* $p < 0.01$). Plot was downloaded from the online database TNMplot (https://tnmplot.com/analysis/). Boxplot represents the median, interquartile range, upper whisker, maximum and minimum. Copyright ©: Department of Bioinformatics, Semmelweis University 2021-2023[2]. (**b** and **c**) *COL1A1* gene expression profiles of breast invasive carcinoma tissues (n = 1097 patients) with normal breast tissues (n = 403 patients). (**d** and **e**) *COL1A1* gene expression profiles of pancreatic adenocarcinoma tissues (n = 177 patients) with normal pancreatic tissues (n = 252 patients). The bars in **b**, **d** represent the proportions of tumor samples that show higher expression of the selected gene compared to normal samples at each of the quantile cutoff values (minimum, 1st quartile, median, 3rd quartile, maximum). *P* values in **b**, **d** derived from the Mann–Whitney test comparison between groups.

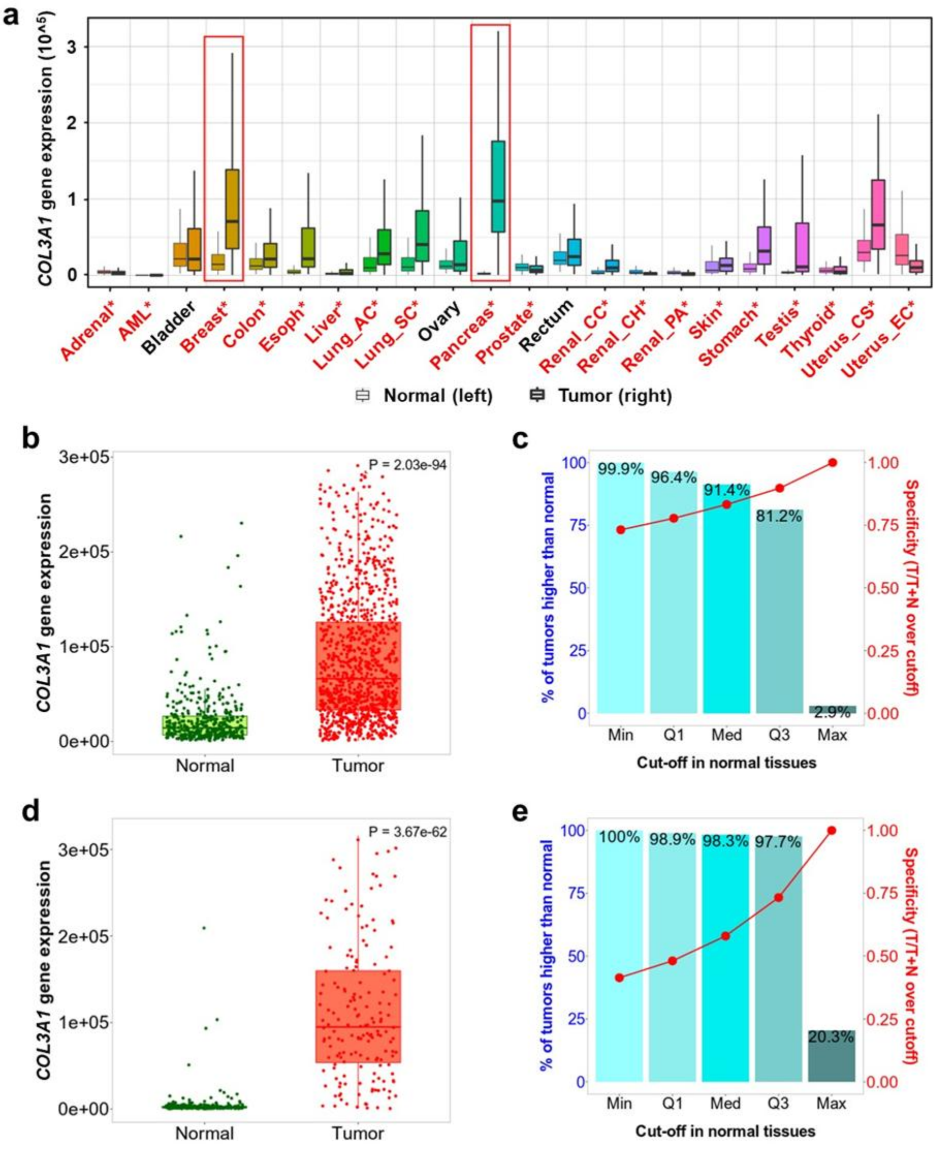

Supplementary Figure 4. *COL3A1* gene expression in normal and tumor tissues. (**a**) *COL3A1* gene expression in different normal and tumor tissues (n = 15648 normal, 40442 tumor samples). Significant differences by a two-sided Mann–Whitney U test are marked with red color (* $p < 0.01$). Plot was downloaded from the online database TNMplot (https://tnmplot.com/analysis/). Boxplot represents the median, interquartile range, upper whisker, maximum and minimum. Copyright ©: Department of Bioinformatics, Semmelweis University 2021-2023[2]. (**b** and **c**) *COL3A1* gene expression profiles of invasive breast carcinoma tissues (n = 1097 patients) with normal breast tissues (n = 403 patients). (**d** and **e**) *COL3A1* gene expression profiles of pancreatic adenocarcinoma tissues (n = 177 patients) with normal pancreatic tissues (n = 252 patients). The bars in **b**, **d** represent the proportions of tumor samples that show higher expression of the selected gene compared to normal samples at each of the quantile cutoff values (minimum, 1st quartile, median, 3rd quartile, maximum). *P* values in **b**, **d** derived from the Mann–Whitney test comparison between groups.

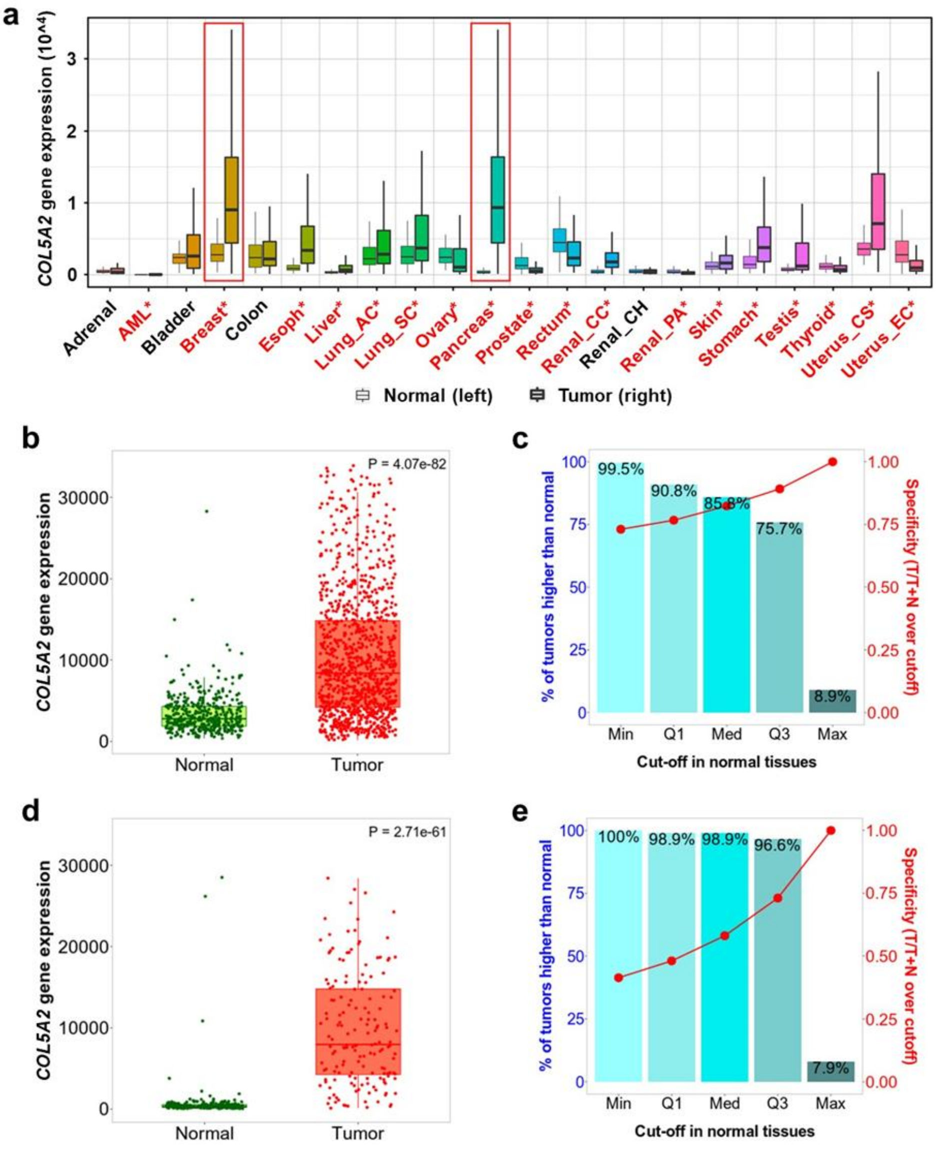

Supplementary Figure 5. *COL5A2* gene expression in normal and tumor tissues. (**a**) *COL5A2* gene expression in different normal and tumor tissues (n = 15648 normal, 40442 tumor samples). Significant differences by a two-sided Mann–Whitney U test are marked with red color (* $p < 0.01$). Plot was downloaded from the online database TNMplot (https://tnmplot.com/analysis/). Boxplot represents the median, interquartile range, upper whisker, maximum and minimum. Copyright ©: Department of Bioinformatics, Semmelweis University 2021-2023[2]. (**b** and **c**) *COL5A2* gene expression profiles of breast invasive carcinoma tissues (n = 1097 patients) with normal breast tissues (n = 403 patients). (**d** and **e**) *COL5A2* gene expression profiles of pancreatic adenocarcinoma tissues (n = 177 patients) with normal pancreatic tissues (n = 252 patients). The bars in **b**, **d** represent the proportions of tumor samples that show higher expression of the selected gene compared to normal samples at each of the quantile cutoff values (minimum, 1st quartile, median, 3rd quartile, maximum). *P* values in **b**, **d** derived from the Mann–Whitney test comparison between groups.

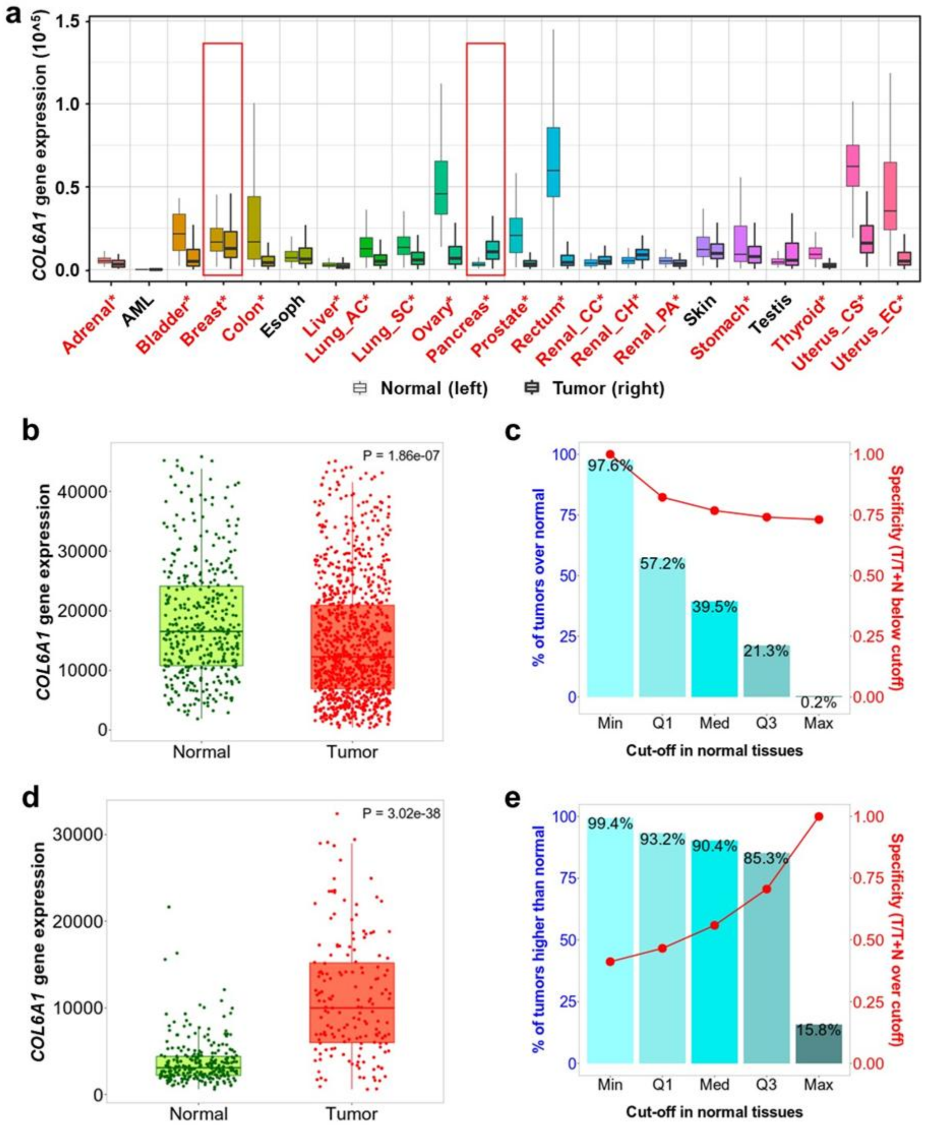

Supplementary Figure 6. ***COL6A1*** **gene expression in normal and tumor tissues.** (**a**) *COL6A1* gene expression in different normal and tumor tissues (n = 15648 normal, 40442 tumor samples). Significant differences by a two-sided Mann–Whitney U test are marked with red color (* $p < 0.01$). Plot was downloaded from the online database TNMplot (https://tnmplot.com/analysis/). Boxplot represents the median, interquartile range, upper whisker, maximum and minimum. Copyright ©: Department of Bioinformatics, Semmelweis University 2021-2023[2]. (**b** and **c**) *COL6A1* gene expression profiles of breast invasive carcinoma tissues (n = 1097 patients) with normal breast tissues (n = 403 patients). (**d** and **e**) *COL6A1* gene expression profiles of pancreatic adenocarcinoma tissues (n = 177 patients) with normal pancreatic tissues (n = 252 patients). The bars in **b**, **d** represent the proportions of tumor samples that show higher expression of the selected gene compared to normal samples at each of the quantile cutoff values (minimum, 1st quartile, median, 3rd quartile, maximum). *P* values in **b**, **d** derived from the Mann–Whitney test comparison between groups.

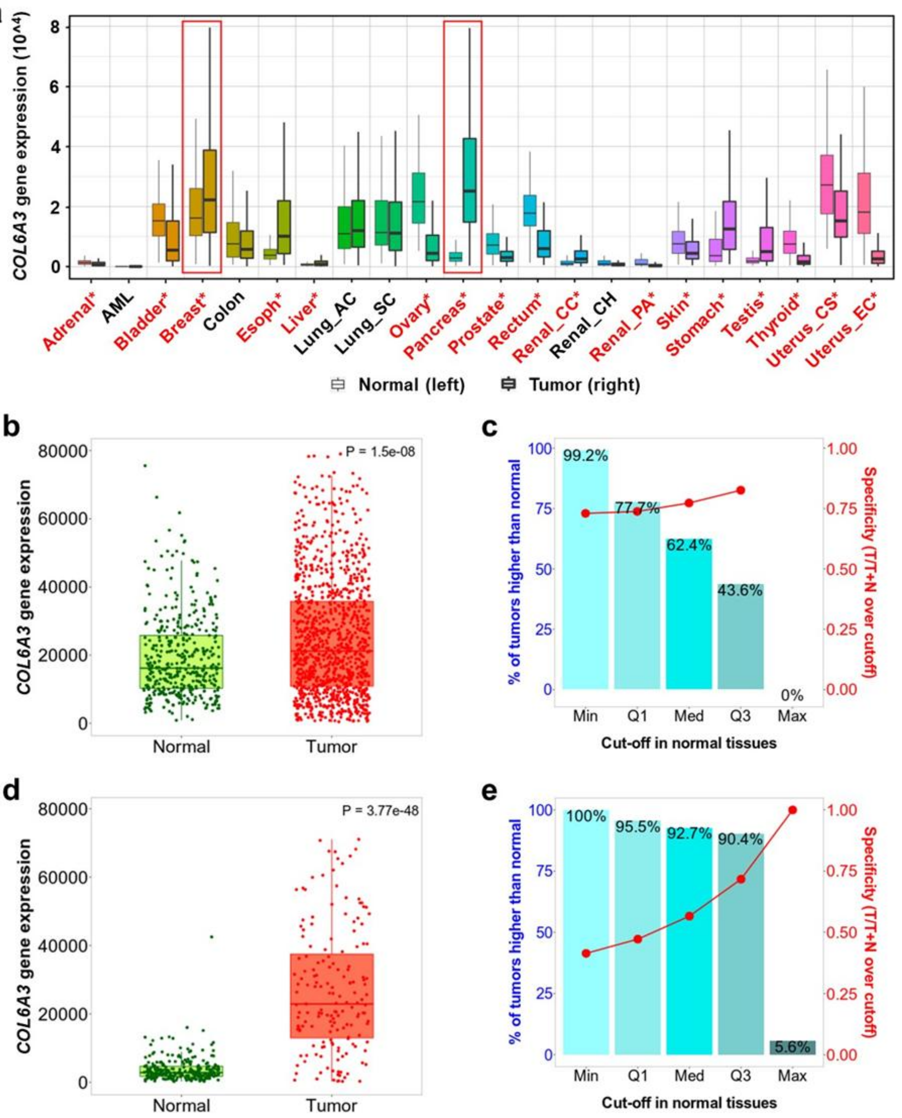

Supplementary Figure 7. ***COL6A3*** **gene expression in normal and tumor tissues.** (**a**) *COL6A3* gene expression in different normal and tumor tissues (n = 15648 normal, 40442 tumor samples). Significant differences by a two-sided Mann–Whitney U test are marked with red color (* $p < 0.01$). Plot was downloaded from the online database TNMplot (https://tnmplot.com/analysis/). Boxplot represents the median, interquartile range, upper whisker, maximum and minimum. Copyright ©: Department of Bioinformatics, Semmelweis University 2021-2023[2]. (**b** and **c**) *COL6A3* gene expression profiles of breast invasive carcinoma tissues (n = 1097 patients) with normal breast tissues (n = 403 patients). (**d** and **e**) *COL6A3* gene expression profiles of pancreatic adenocarcinoma tissues (n = 177 patients) with normal pancreatic tissues (n = 252 patients). The bars in **b**, **d** represent the proportions of tumor samples that show higher expression of the selected gene compared to normal samples at each of the quantile cutoff values (minimum, 1st quartile, median, 3rd quartile, maximum). *P* values in **b**, **d** derived from the Mann–Whitney test comparison between groups.

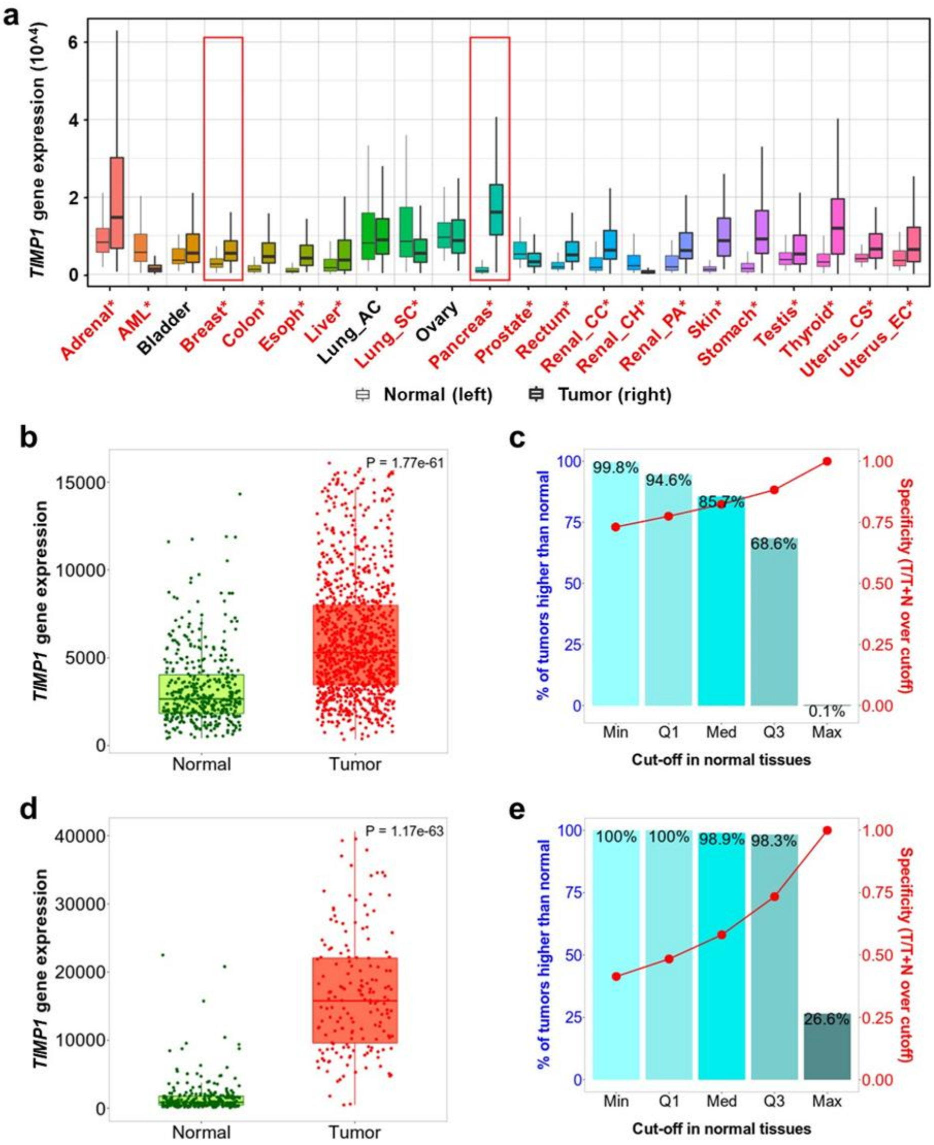

Supplementary Figure 8. *TIMP1* gene expression in normal and tumor tissues. (**a**) *TIMP1* gene expression in different normal and tumor tissues (n = 15648 normal, 40442 tumor samples). Significant differences by a two-sided Mann–Whitney U test are marked with red color (* $p < 0.01$). Plot was downloaded from the online database TNMplot (https://tnmplot.com/analysis/). Boxplot represents the median, interquartile range, upper whisker, maximum and minimum. Copyright ©: Department of Bioinformatics, Semmelweis University 2021-2023[2]. (**b** and **c**) *TIMP1* gene expression profiles of breast invasive carcinoma tissues (n = 1097 patients) with normal breast tissues (n = 403 patients). (**d** and **e**) *TIMP1* gene expression profiles of pancreatic adenocarcinoma tissues (n = 177 patients) with normal pancreatic tissues (n = 252 patients). The bars in **b**, **d** represent the proportions of tumor samples that show higher expression of the selected gene compared to normal samples at each of the quantile cutoff values (minimum, 1st quartile, median, 3rd quartile, maximum). *P* values in **b**, **d** derived from the Mann–Whitney test comparison between groups.

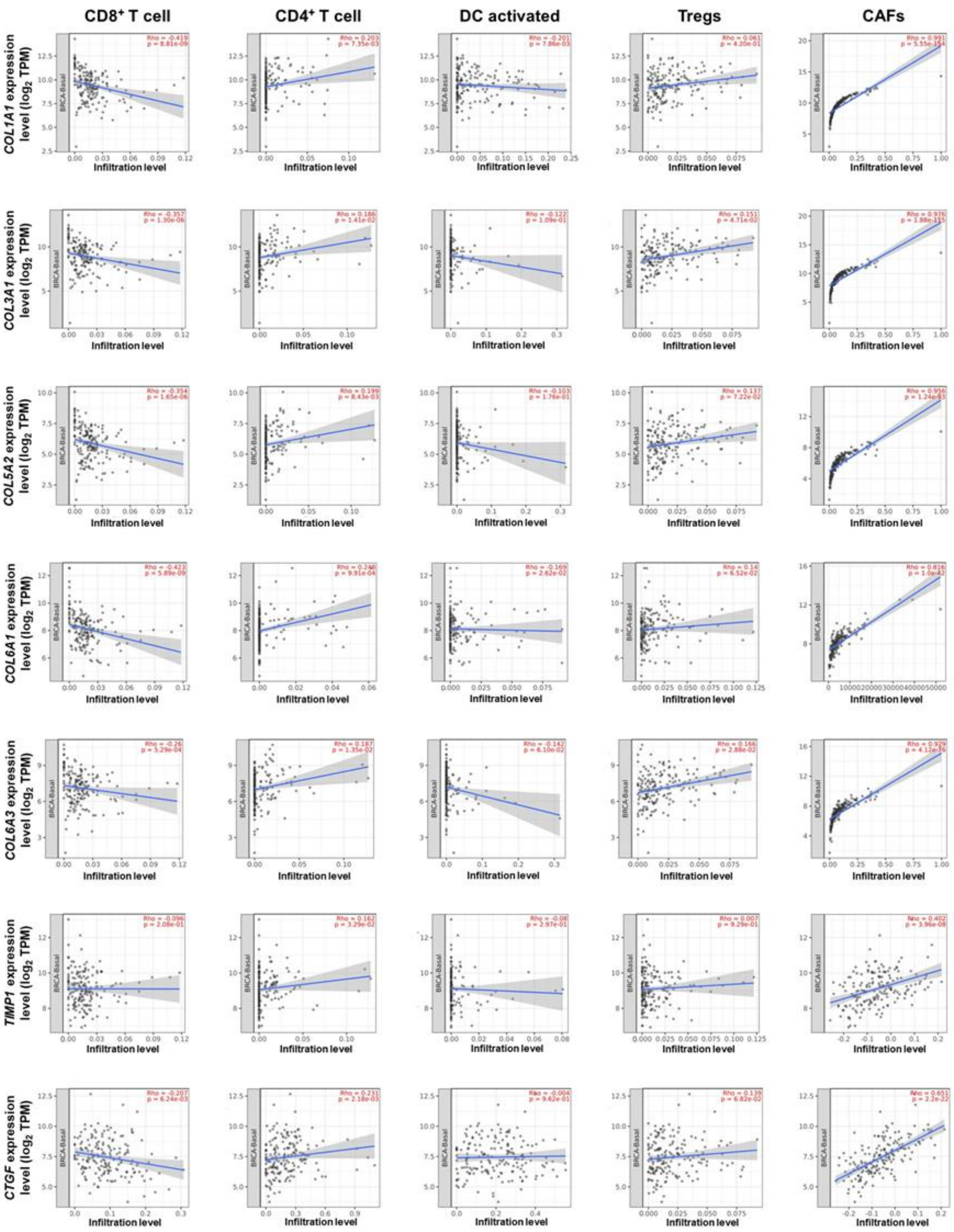

Supplementary Figure 9. The correlations between TGF-β target genes (e.g., *COL1A1*, *COL3A1*, *COL5A2*, *COL6A1*, *COL6A3*, *TIMP1*, *CTGF*) expression and intratumoral infiltration of $CD4^+$ T cell, Tregs, $CD8^+$ T cell, myeloid DC activated and CAFs in breast invasive carcinoma (BRCA-Basal) patients (n = 191) in the TIMER database. Shaded error bands depict the standard error. Spearman test was used to determine the correlation coefficients.

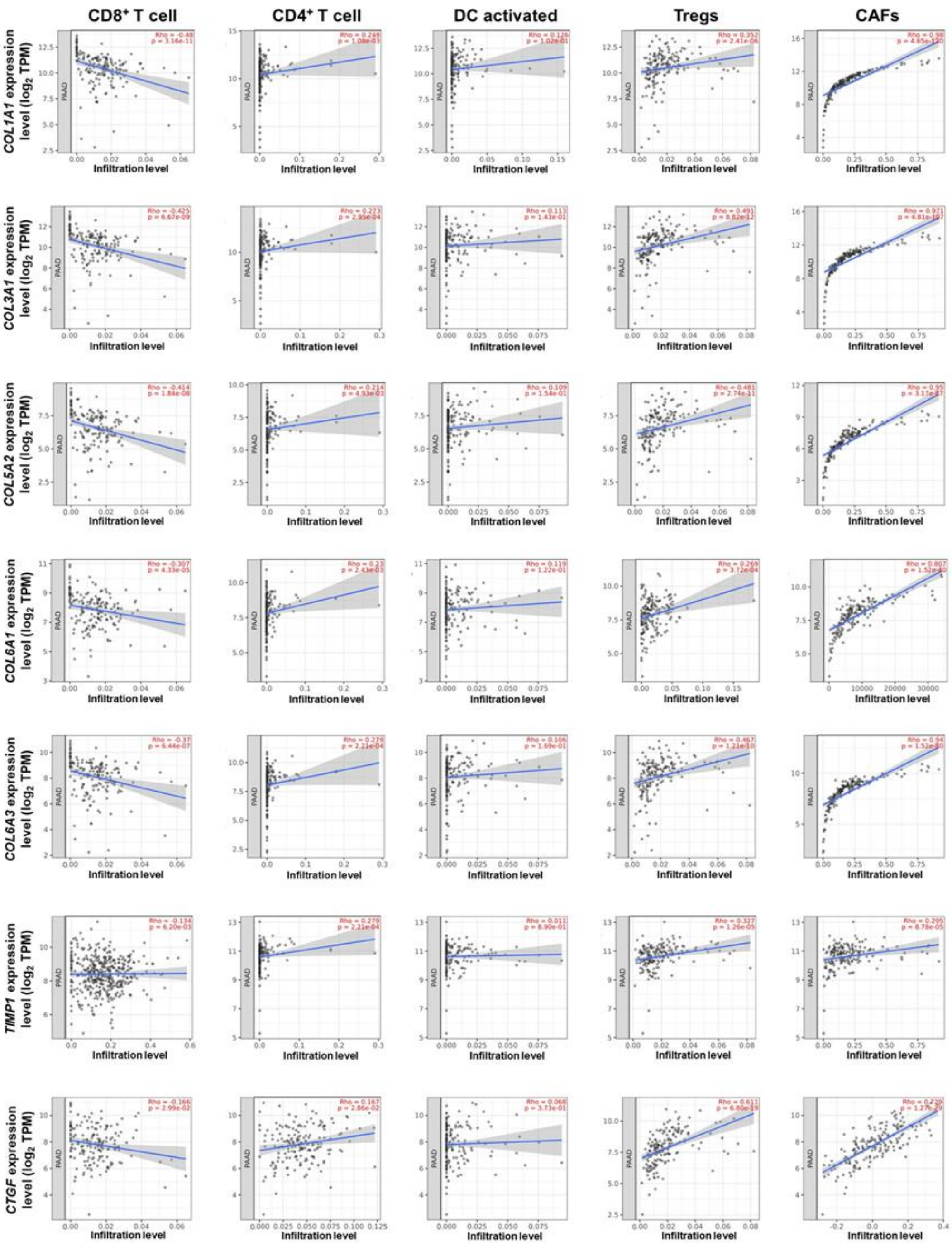

Supplementary Figure 10. The correlations between TGF-β target genes (e.g., *COL1A1*, *COL3A1*, *COL5A2*, *COL6A1*, *COL6A3*, *TIMP1*, *CTGF*) expression and tumor-infiltrating $CD4^+$ T cell, Tregs, $CD8^+$ T cell, myeloid DC activated and CAFs in pancreatic adenocarcinoma tissues (from 179 patients) in the TIMER database. Shaded error bands depict the standard error. Spearman test was used to determine the correlation coefficients.

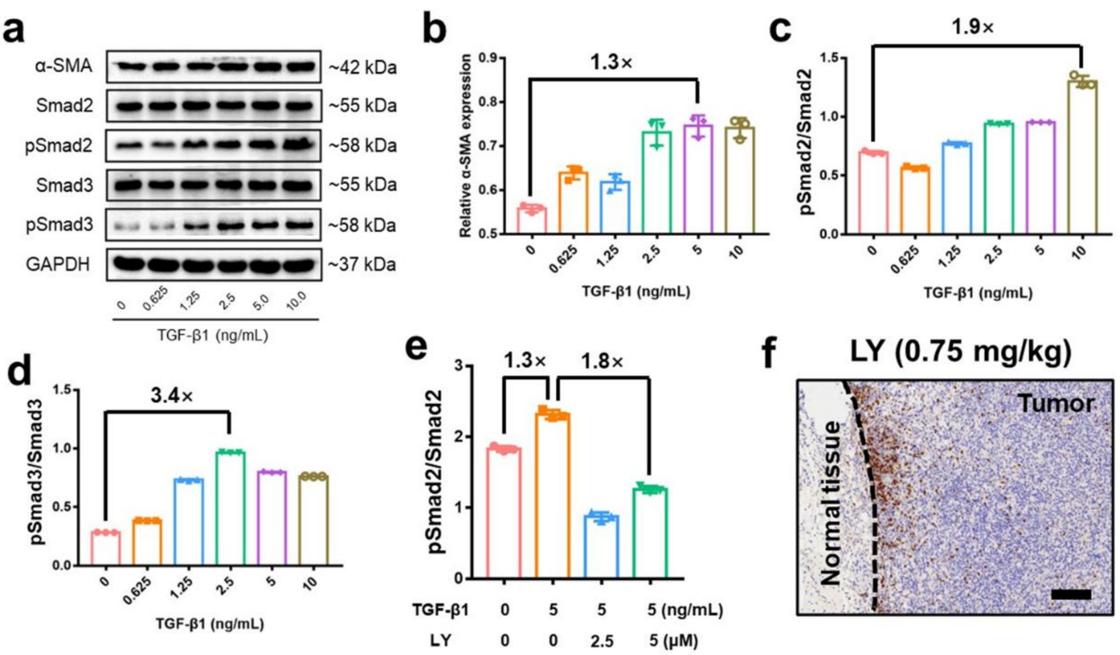

Supplementary Figure 11. WB assay of the influence of TGF-β1 and LY on α-SMA expression and phosphorylated Smad2/3 activation in NIH3T3 cells *in vitro*. (**a**) WB analysis of TGF-β1-induced α-SMA, phosphorylated (p-)Smad2/3 expression in NIH3T3 cells *in vitro* (*n* = 3 biologically independent cells). (**b-d**) GAPDH-normalized (**b**) total α-SMA, (**c**) phosphorylated Smad2/Smad2 and (**d**) phosphorylated Smad3/Smad3 ratios in NIH3T3 cells *in vitro* (n = 3 biologically independent cells). (**e**) LY-suppressed TGF-β1-induced activation of phosphorylated Smad2 in Fig. 2**f** (n = 3 biologically independent cells). (**f**) Representative CD3 staining of tumor periphery and centre upon intratumoral injection of LY (0.75 mg/kg, *n* = 3 mice). Scale bar = 100 μm. Error bars represent mean ± SD. The experiment was repeated independently 3 times with similar results in **a**, **f**.

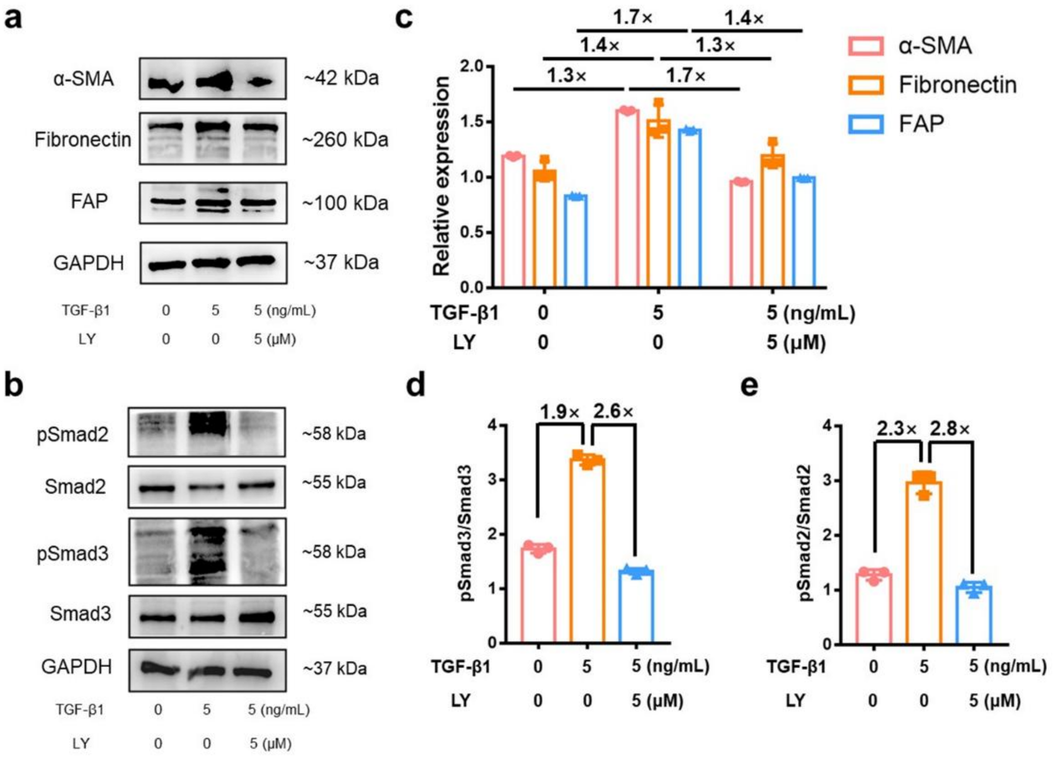

Supplementary Figure 12. (**a** and **b**) WB assays of Smad2/3, pSmad2/3, α-SMA, FAP, and fibronectin expressions upon TGF-β1 and LY incubation in the primary CAFs of Panc02 tumor *in vitro*. (**c**) Normalized α-SMA, FAP, and fibronectin expression in the primary CAFs from (**a**) (mean ± SD, n = 3 biologically independent cells). (**d** and **e**) Normalized pSmad2/Smad2, pSmad3/Smad3 ratios in the primary CAFs from (**b**) (mean ± SD, n = 3 biologically independent cells).

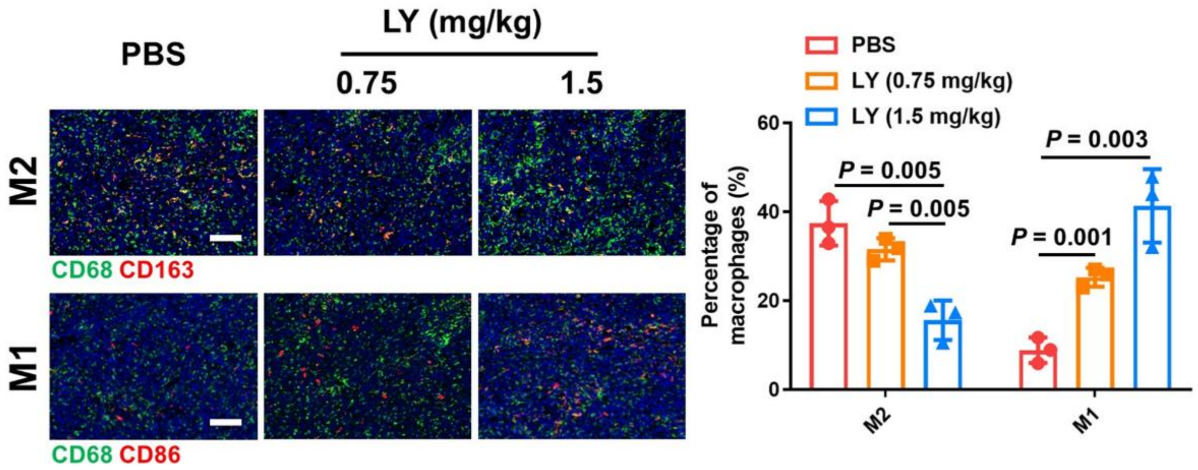

Supplementary Figure 13. Immunofluorescence staining and proportions of the tumor-infiltrating M1 and M2 macrophages with various treatments (scale bar = 100 μm) (mean ± SD, n = 3 mice). *P* values derived from the Student's *t*-test (two-tailed, two-sample unequal variance).

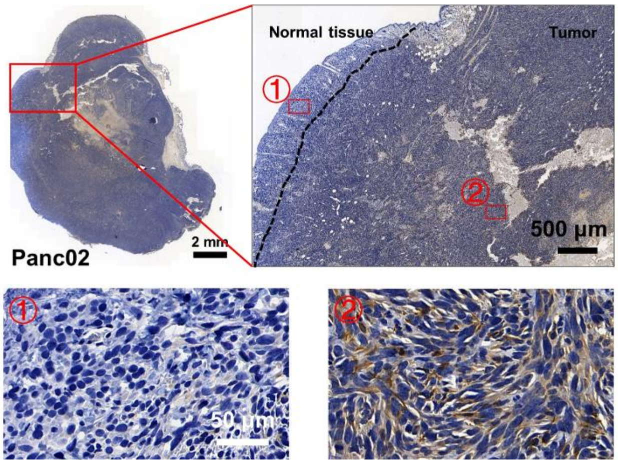

Supplementary Figure 14. Immunohistochemical staining of MMP-2 in a mouse model of Panc02 pancreatic adenocarcinoma tumor ex-vivo (n = 3 mice). The experiment was repeated independently 3 times with similar results.

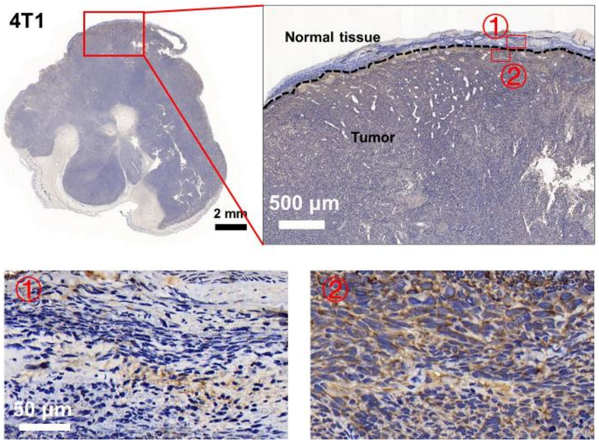

Supplementary Figure 15. Immunohistochemical staining of MMP-2 in a mouse model of 4T1 triple-negative breast cancer (TNBC) tumor ex-vivo (n = 3 mice). The experiment was repeated independently 3 times with similar results.

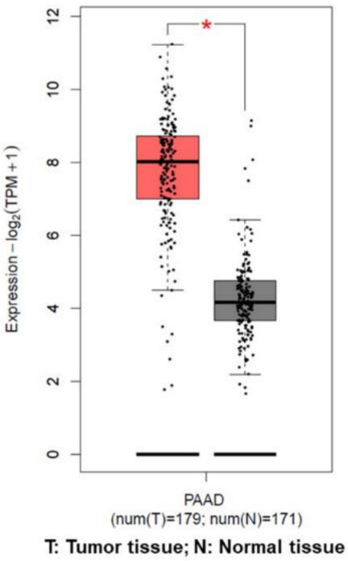

Supplementary Figure 16. *MMP-2* gene expression in pancreatic adenocarcinoma (PAAD) tissues and normal pancreatic tissues in the GEPIA dataset. Boxplot represents the median, interquartile range, upper and lower whisker, maximum and minimum. Plot was downloaded from the online database GEPIA (http://gepia.cancer-pku.cn/detail.php?gene=&clicktag=boxplot). *P*-value cutoff = 0.01 (Student's *t* test, significant difference is marked with red*).

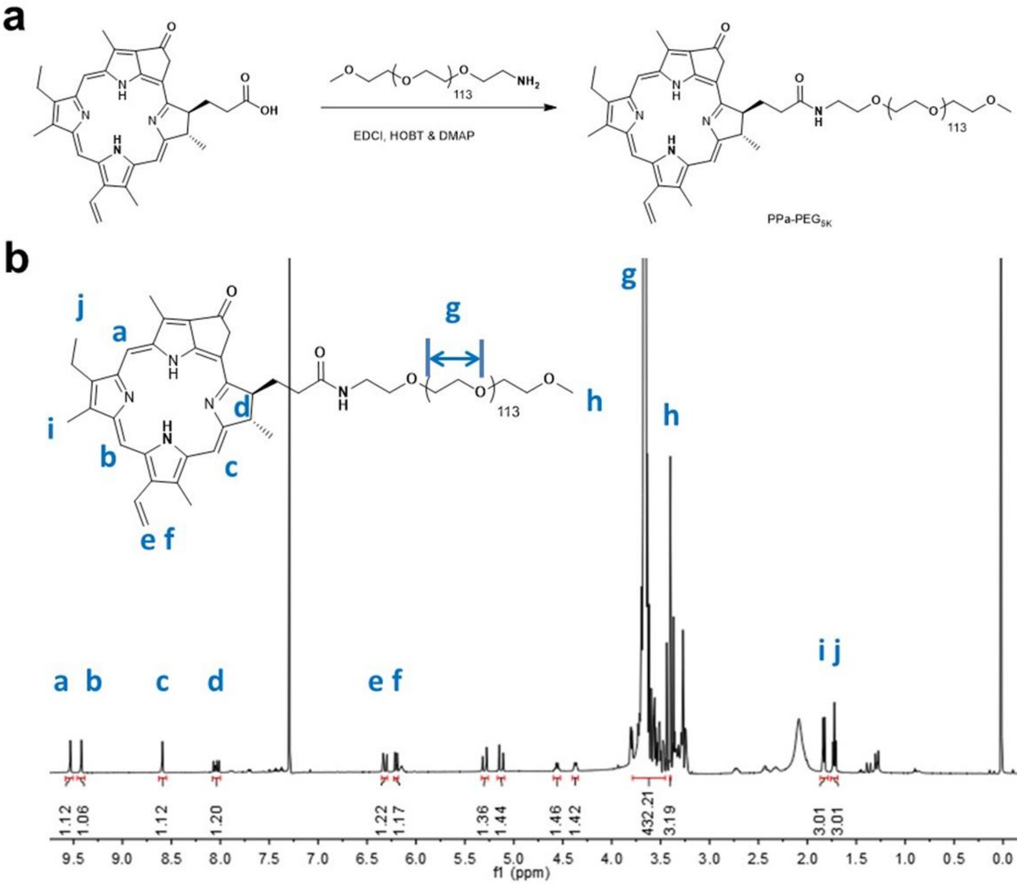

Supplementary Figure 17. Synthesis and characterization of PPa-PEG$_{5k}$. (**a**) The synthetic route of PPa-PEG$_{5k}$. (**b**) ^{1}H-NMR spectrum of PPa-GPLGLAG-PEG$_{5k}$.

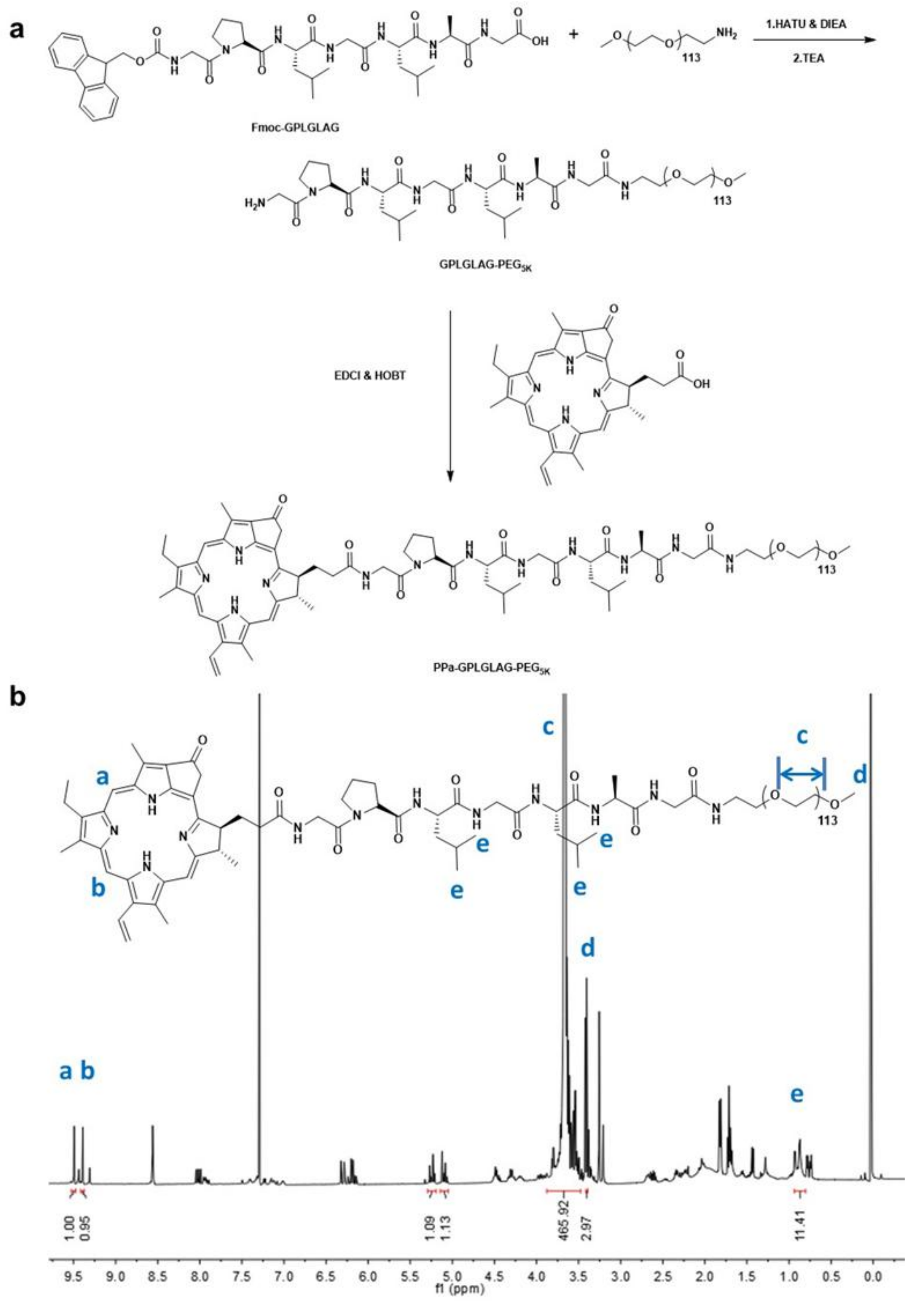

Supplementary Figure 18. Synthesis and characterization of PPa-GPLGLAG-PEG$_{5k}$. (**a**) The synthetic route of PPa-GPLGLAG-PEG$_{5k}$. (**b**) ^{1}H-NMR spectrum of PPa-GPLGLAG-PEG$_{5k}$ conjugate.

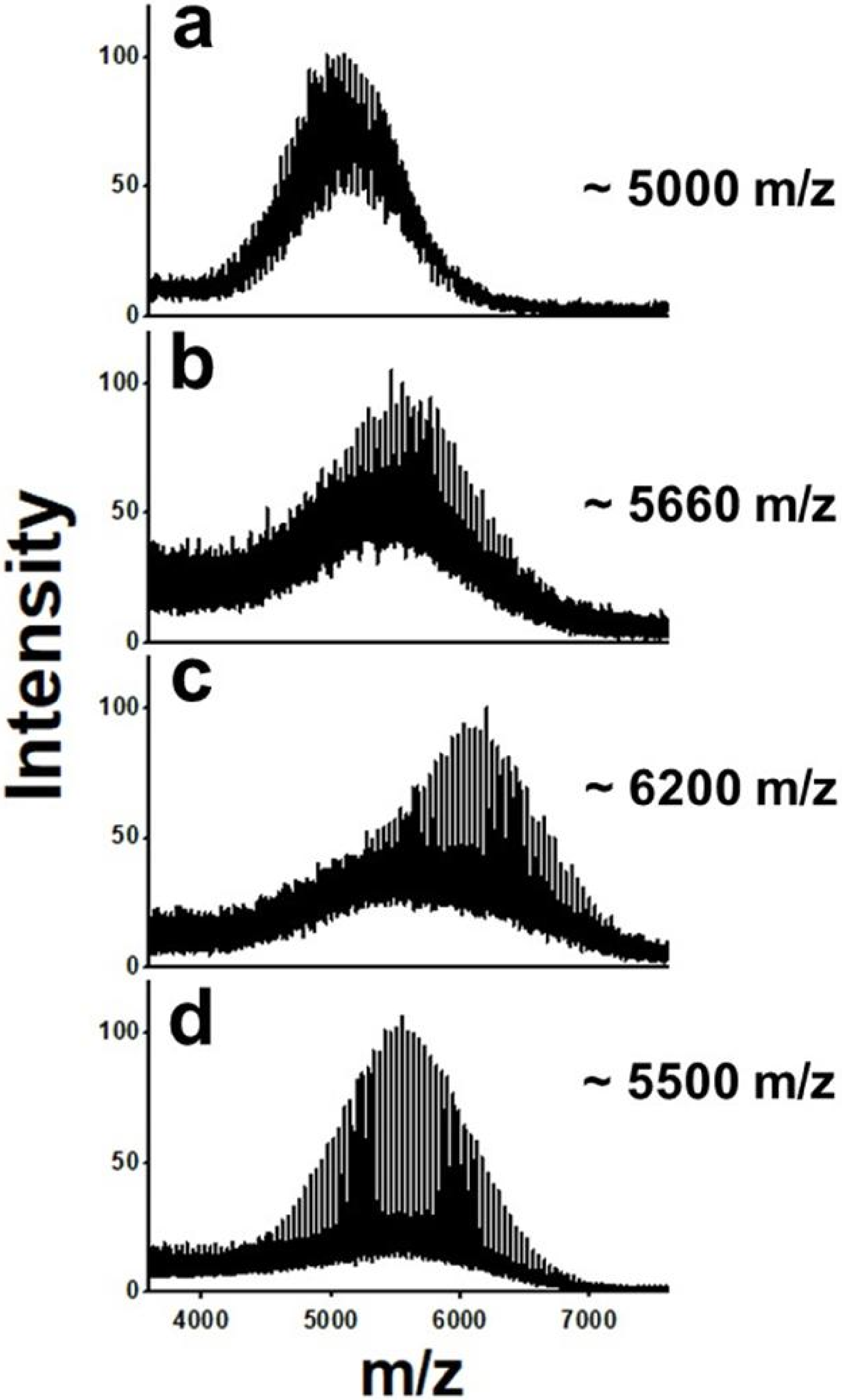

Supplementary Figure 19. Characterization of PEG derivatives. MALDI-TOF MS spectra of (**a**) PEG_{5k}-NH_2, (**b**) NH_2-GPLGLAG-PEG_{5k}, (**c**) PPa-GPLGLAG-PEG_{5k}, and (**d**) PPa-PEG_{5k}.

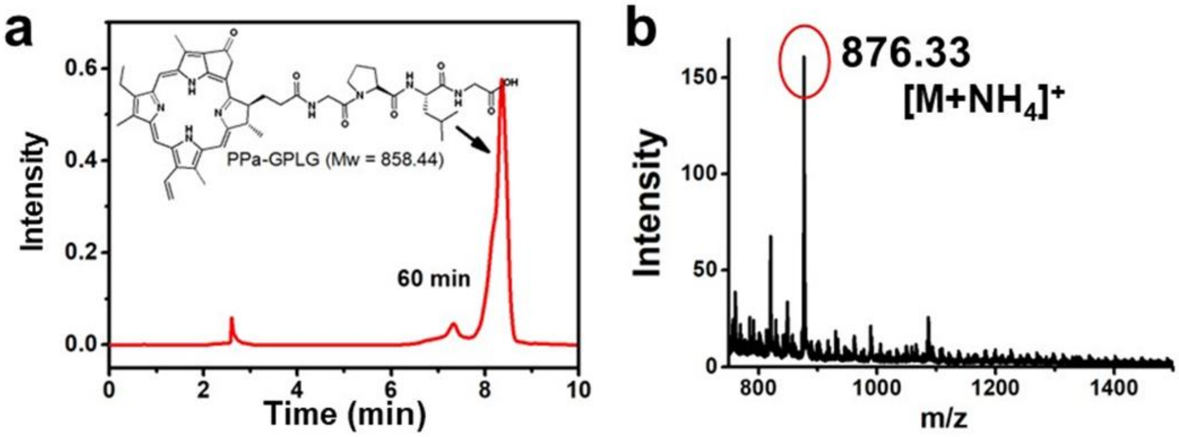

Supplementary Figure 20. (**a**) HPLC and (**b**) MALDI-TOF MS spectra examination of PPa-GPLGLAG-PEG_{5k} upon 60 min incubation with 40 μg/mL of MMP-2.

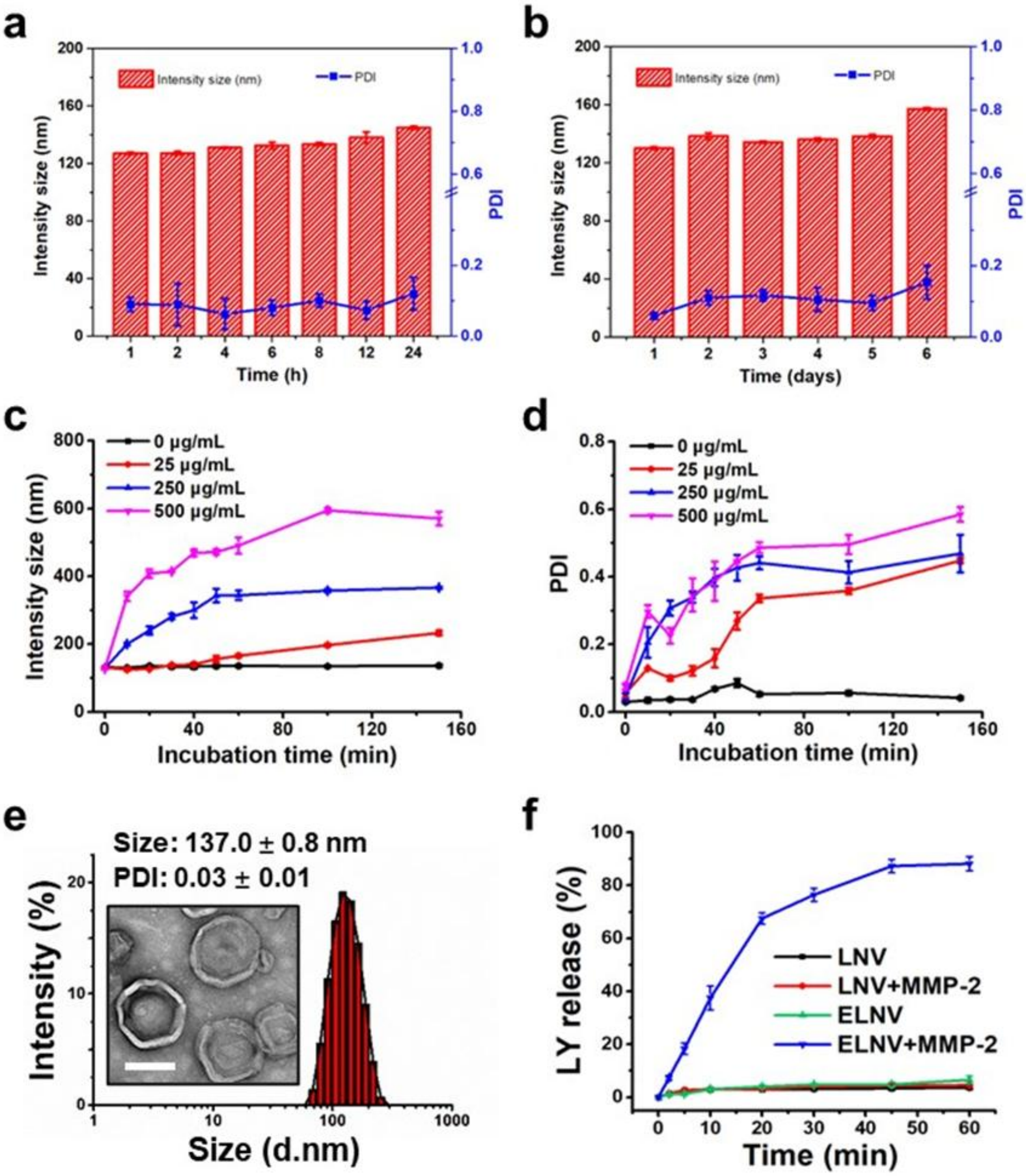

Supplementary Figure 21. Characterization of the physicochemical properties of the ELNV nanovesicles. (**a**) Serum stability. (**b**) DLS-determined long-term stability of ELNV. (**c** and **d**) Changes in size (**c**) and PDI (**d**) of ELNV after incubating with MMP-2 at different times. (**e**) The hydrodynamic diameters and representative TEM image of LNV 4 h post-incubation with MMP-2 (40 μg/mL, 37 °C), Scale bar = 100 nm. The experiment was repeated independently 3 times with similar results. (**f**) Cumulative LY release from LNV or ELNV nanovesicles with or without 40 μg/mL of MMP-2 incubation. All data are presented as mean ± SD. *n* = 3 independent experiments.

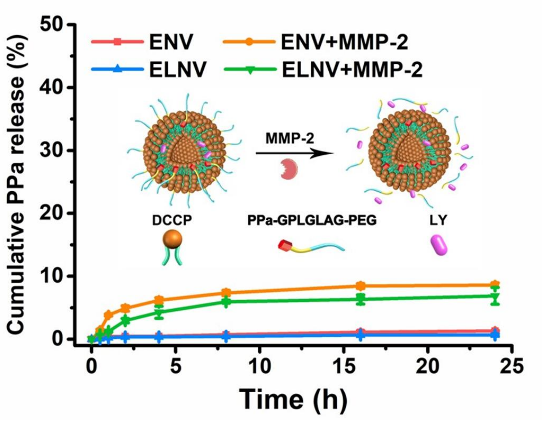

Supplementary Figure 22. The cumulative PPa release from ENV and ELNV with or without MMP-2 incubation (mean ± SD, n = 3 independent experiments).

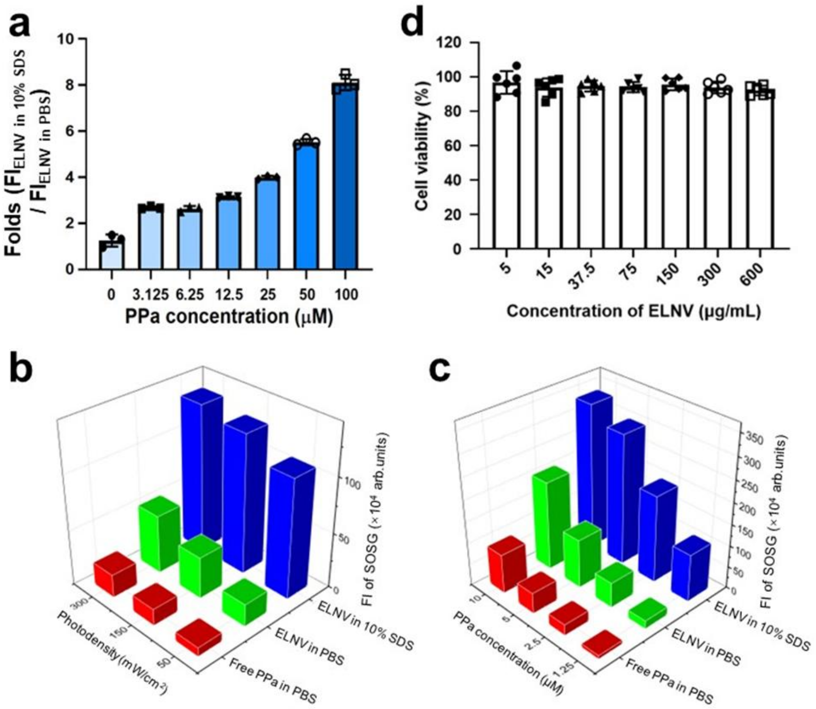

Supplementary Figure 23. The photoactivity and biocompatibility of ELNV nanovesicles. (**a**) The ratios of the PPa fluorescence intensity of ELNV in 10% SDS and PBS (upon the different PPa concentrations) (mean ± SD, n = 3 independent experiments). (**b**) The generation of $^1O_2$ by free PPa in PBS, ELNV in PBS, and 10% SDS, under different photodensity of 671 nm laser, was measured using SOSG as a fluorescent probe (Ex/Em = 504/525, the PPa concentration is 1.25 μM). (**c**) The generation of $^1O_2$ by free PPa in PBS, ELNV in PBS, and 10% SDS, under different concentrations of PPa, was measured using SOSG as a fluorescent probe (Ex/Em = 504/525, the photodensity is 150 mW/cm^2). (**d**) Cell viability of ELNV in 4T1 cells (mean ± SD, n = 6 biologically independent

cells).

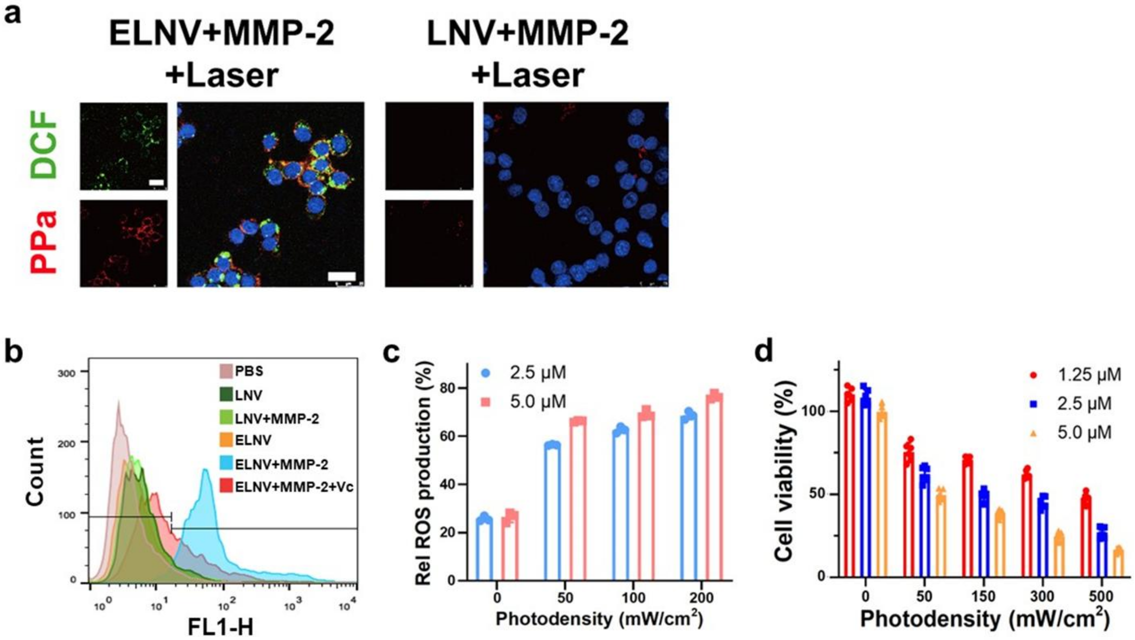

Supplementary Figure 24. The intracellular ROS generation and PDT of ELNV nanovesicles. (**a**) Fluorescence images of 4T1 cells stained with 10 μM of DCFH-DA upon different treatments. Scale bars = 25 μm. The experiment was repeated independently 3 times with similar results. (**b**) The histogram represents the intracellular ROS generation of different nanovesicles measured by flow cytometry. (**c**) Flow cytometry quantitative analysis of the production efficiency of ROS with different power densities and PPa concentrations (mean ± SD, n = 3 biologically independent cells). (**d**) The phototoxicity of ELNV in 4T1 cells upon different conditions (mean ± SD, n = 6 biologically independent cells).

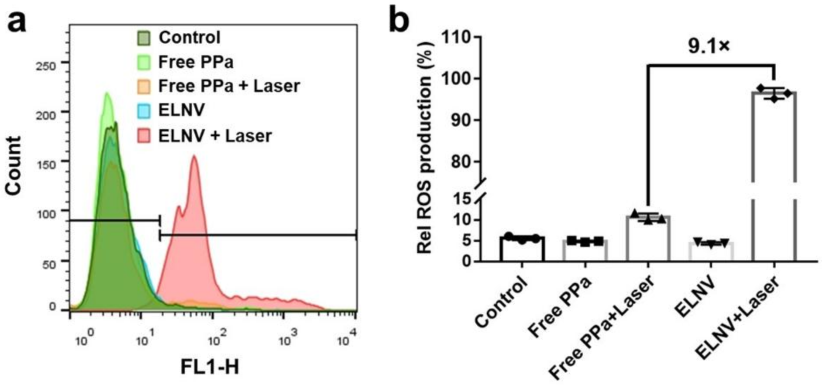

Supplementary Figure 25. (**a**) The histogram represents the intracellular ROS generation of different free PPa and nanovesicles measured by flow cytometry. (**b**) Flow cytometric analysis of intracellular ROS generation in 4T1 cells upon the 671 nm laser irradiation (1 min at a photodensitiy of 150 mW/cm^2) (mean ± SD, n = 3 biologically independent cells).

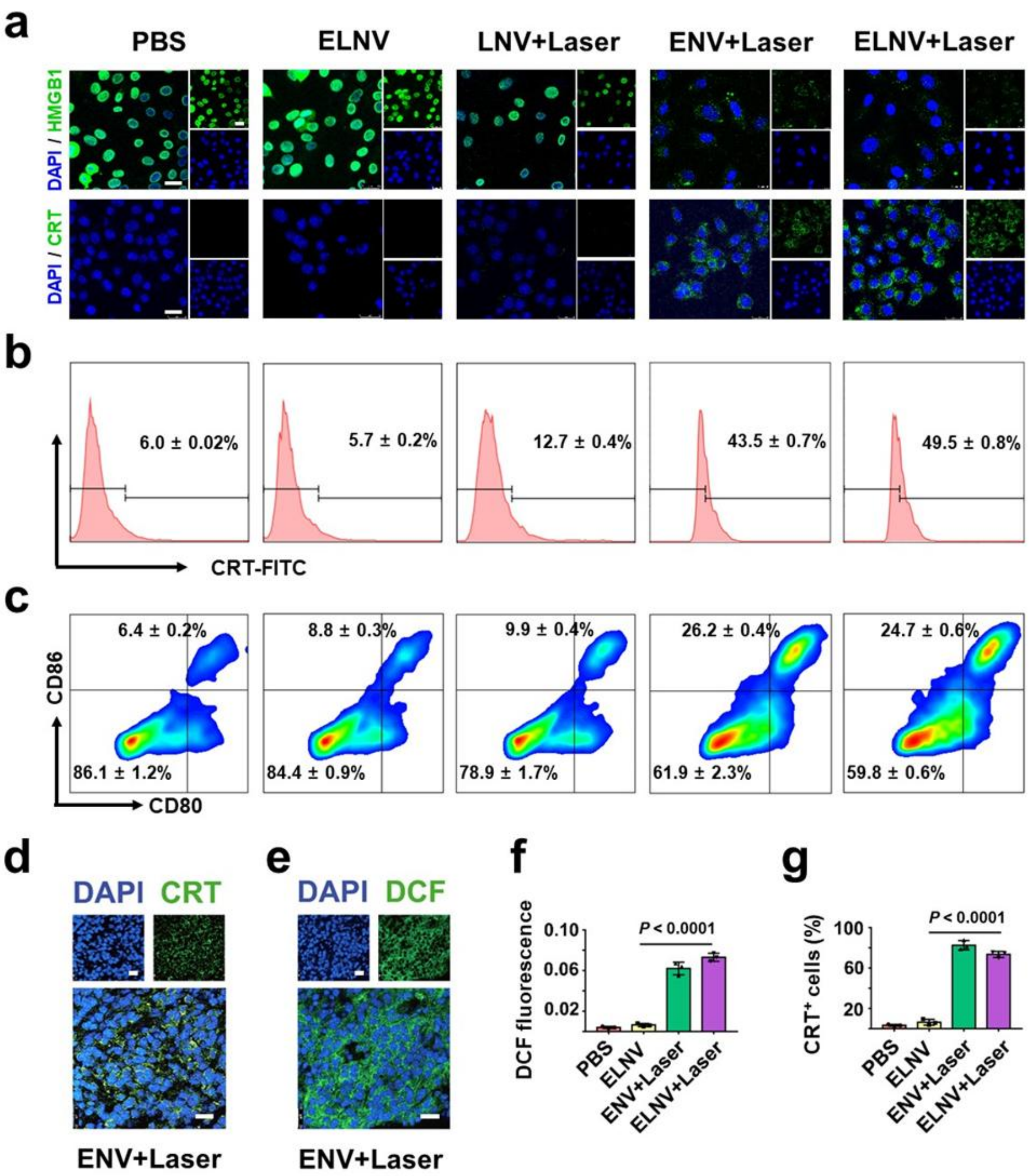

Supplementary Figure 26. PDT-induced ICD effect of ELNV nanovesicles *in vitro* and *in vivo*. (**a**) CLSM images of PDT-induced extracellular efflux of HMGB1 release and CRT exposure on the membrane of the tumor cells (Scale bar = 25 μm). The experiment was repeated independently 3 times with similar results. (**b**) Representative flow cytometry histogram of CRT exposure *in vitro*. (**c**) Flow cytometry examination of DCs maturation before co-incubation with different nanovesicles-pretreated 4T1 cells. (**d** and **e**) Fluorescence images of (**d**) CRT exposure and (**e**) ROS generation in 4T1 tumor sections *in vivo*. Scale bars = 25 μm. (**f** and **g**) Fluorescence semi-quantitative analysis of ROS generation (**f**) and CRT exposure (**g**) in Fig. 3**l**, and (**d** and **e**) (mean ± SD, n = 3 mice, P = 1.12×10^{-5} and 9.86×10^{-6}). P values derived from the Student's t-test (two-tailed, two-sample unequal variance).

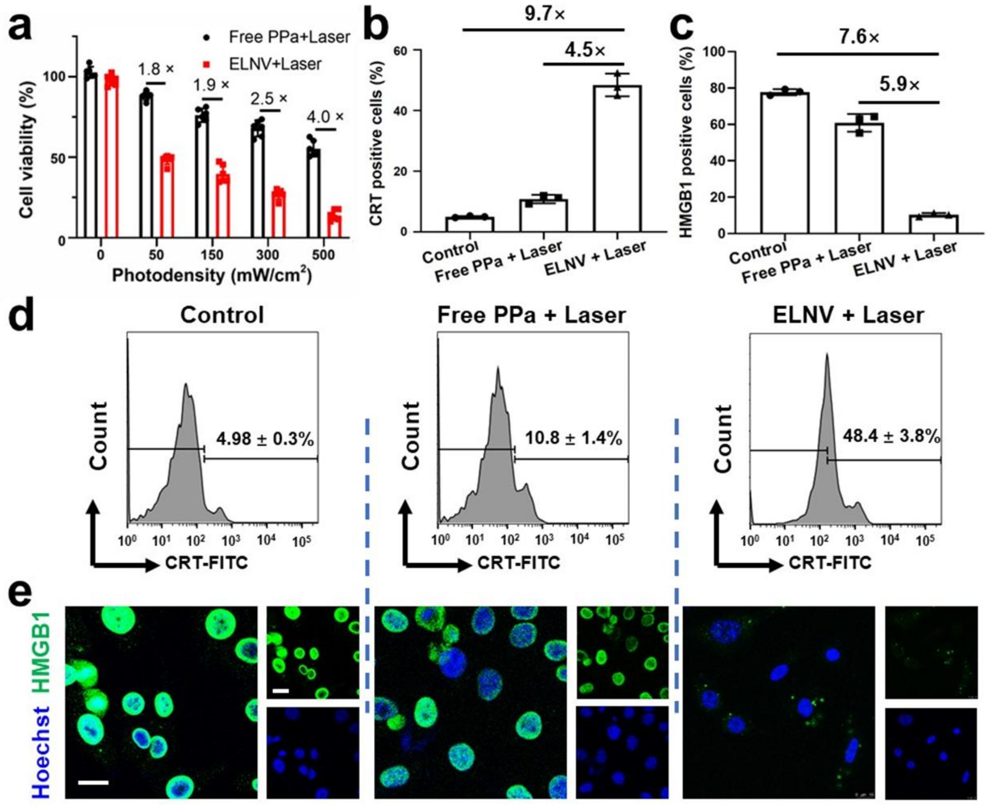

Supplementary Figure 27. (**a**) Phototoxicity of free PPa and ELNV in 4T1 cells upon different conditions (mean ± SD, n = 6 biologically independent cells). (**b** and **c**) PDT-triggered CRT exposure (**b**) and HMGB1 efflux (**c**) in 4T1 cells *in vitro* (mean ± SD, n = 3 biologically independent cells). 4T1 cells were pretreated with free PPa or ELNV for 4 h and irradiated with the 671 nm laser at a PPa concentration of 5 μM. (**d**) Representative flow cytometry histogram of CRT exposure *in vitro* (n = 3). (**e**) CLSM images of PDT-induced extracellular efflux of HMGB1 release of the tumor cells (Scale bar = 20 μm). The experiment was repeated independently 3 times with similar results. Nanovesicles were pretreated with 40 μg/mL of MMP-2 for 24 h at 37 °C before addition to cells (mean ± SD, n = 3).

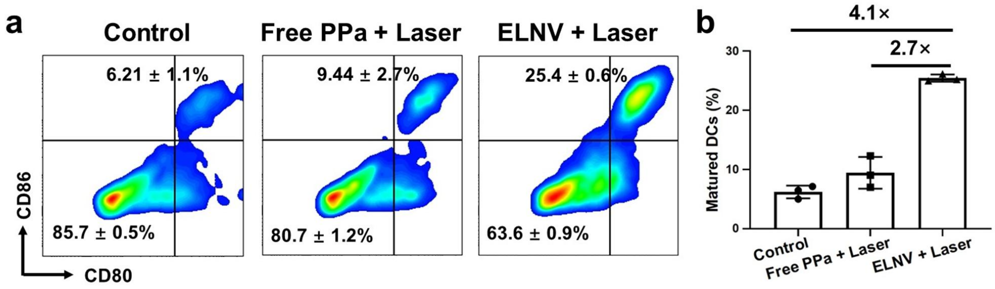

Supplementary Figure 28. (**a**) Flow cytometry examination of DC maturation upon co-incubation with nanovesicle-pretreated 4T1 cells. (**b**) The percentage of PDT-induced DC maturation *in vitro* (mean ± SD, n = 3 biologically independent cells).

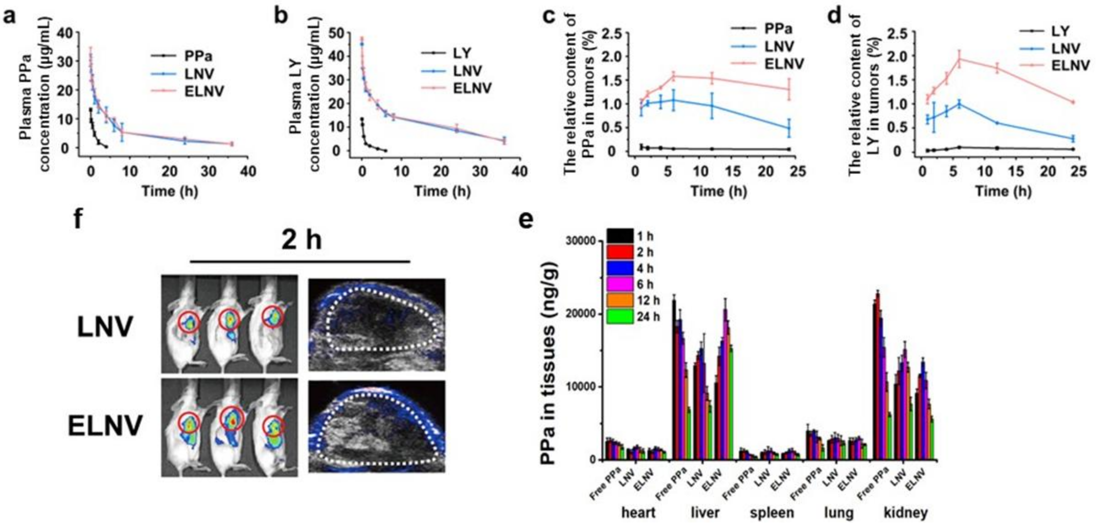

Supplementary Figure 29. Distribution of ELNV nanovesicles *in vivo*. (**a-d**) Plasma concentration or tumor tissue content–time profiles of (**a** and **c**) PPa and (**b** and **d**) LY in mice after intravenous administration of Free PPa, Free LY, LNV, and ELNV at a PPa and LY dose of 5 and 20 mg/kg, respectively. The bioavailability values of PPa, LNV, and ELNV are 14.33 ± 0.61, 182.4 ± 56.84, 202.81 ± 16.25 mg/L*h in (**a**). The bioavailability values of LY, LNV, and ELNV are 14.17 ± 0.60, 420.83 ± 26.70 and 436.019 ± 9.12 mg/L*h in (**b**). (**e**) Distributions of PPa in healthy tissues of mice treated with Free PPa, LNV, or ELNV at a dose of 5 mg/kg over time. (**f**) Fluorescence (left) and PA (right) images of 4T1 tumor-bearing mice 2 h after intravenous injection with LNV or ELNV. The red and white circles indicate the tumor sites. All data are presented as mean ± SD. n = 3 mice.

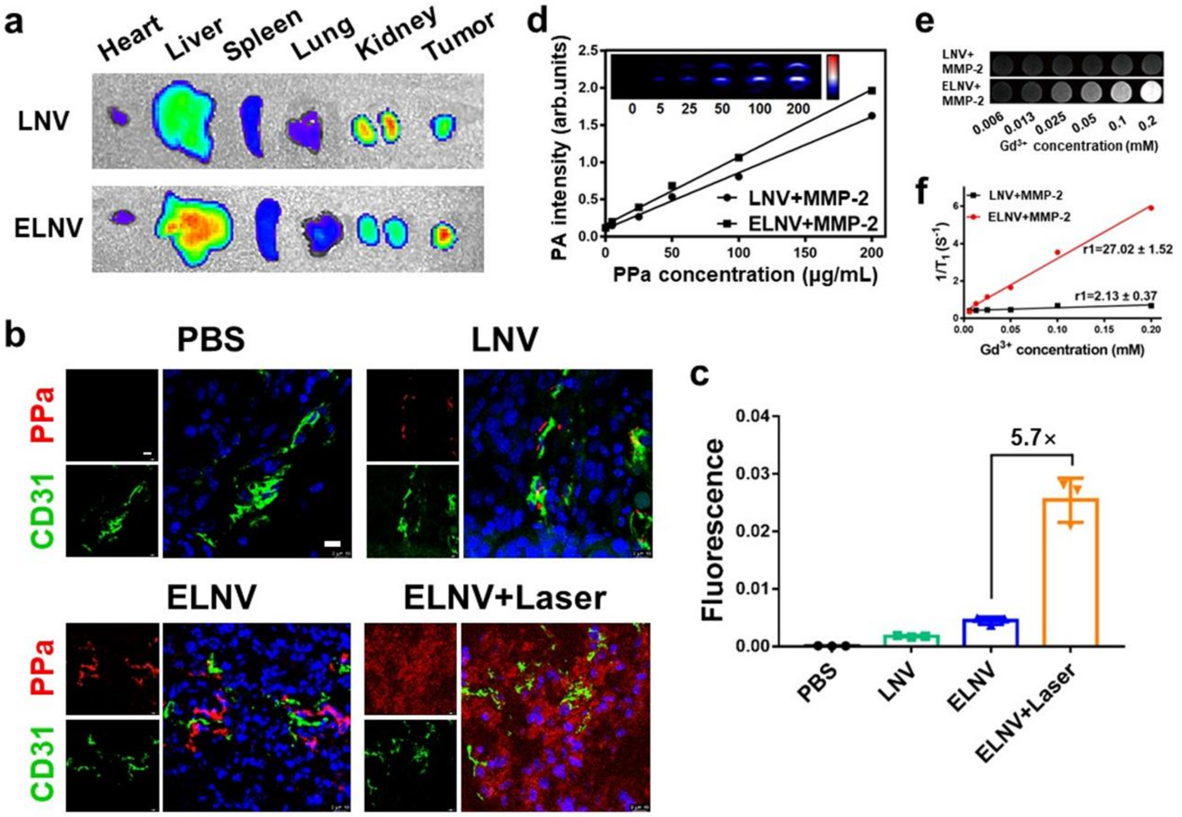

Supplementary Figure 30. Multimodality imaging and tumor penetration of ELNV nanovesicles. (**a**) Representative ex vivo fluorescence images of tumors and other tissues of mice upon LNV or ELNV. (**b**) Analysis of PPa distributions in tumors. The tumor blood vessels were immunostained with anti-CD31 antibody (*n* = 3 mice, Scale bar = 10 μm). The experiment was repeated independently 3 times with similar results. (**c**) PPa fluorescence of CD31-stained tumor tissues was quantified by ImageJ (mean ± SD, n = 4 mice). (**d**) Plots of *in vitro* PA signal versus various PPa concentrations of LNV or ELNV with MMP-2. Inset is the PA images of ELNV nanovesicles with various PPa concentrations of 0, 5, 25, 50, 100, and 200 μg/mL. (**e**) T_1-weighted MR images and (**f**) the corresponding T_1 relaxation rate of gadolinium-loaded ELNV or LNV at various concentrations of gadolinium with MMP-2.

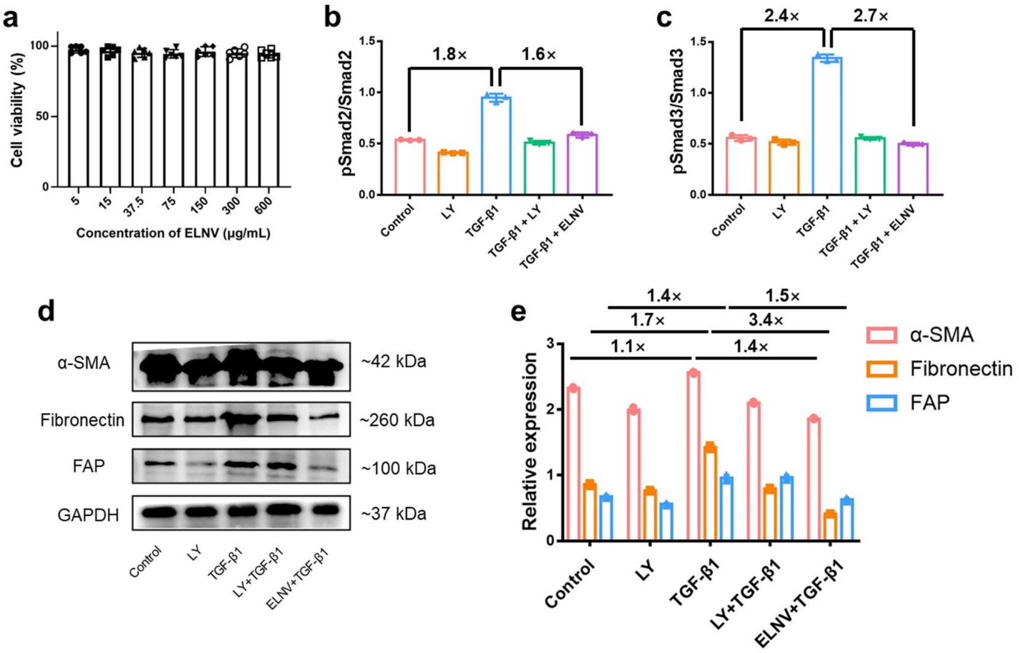

Supplementary Figure 31. The biocompatibility of ELNV nanovesicles and ELNV reverse the activation of CAFs *in vitro*. (**a**) Cell viability of ELNV in NIH3T3 cells (n = 6 biologically independent cells). (**b** and **c**) The GAPDH-normalized phosphorylated (p-) and total Smad2/3 expression of NIH3T3 cells *in vitro* in Fig. 4**a** (n = 3 biologically independent cells). (**d**) WB analysis of the expression of α-SMA, FAP, and fibronectin upon different conditions. (**e**) The GAPDH-normalized α-SMA, FAP and fibronectin expression of NIH3T3 cells *in vitro* (n = 3 biologically independent cells). All data are presented as mean ± SD.

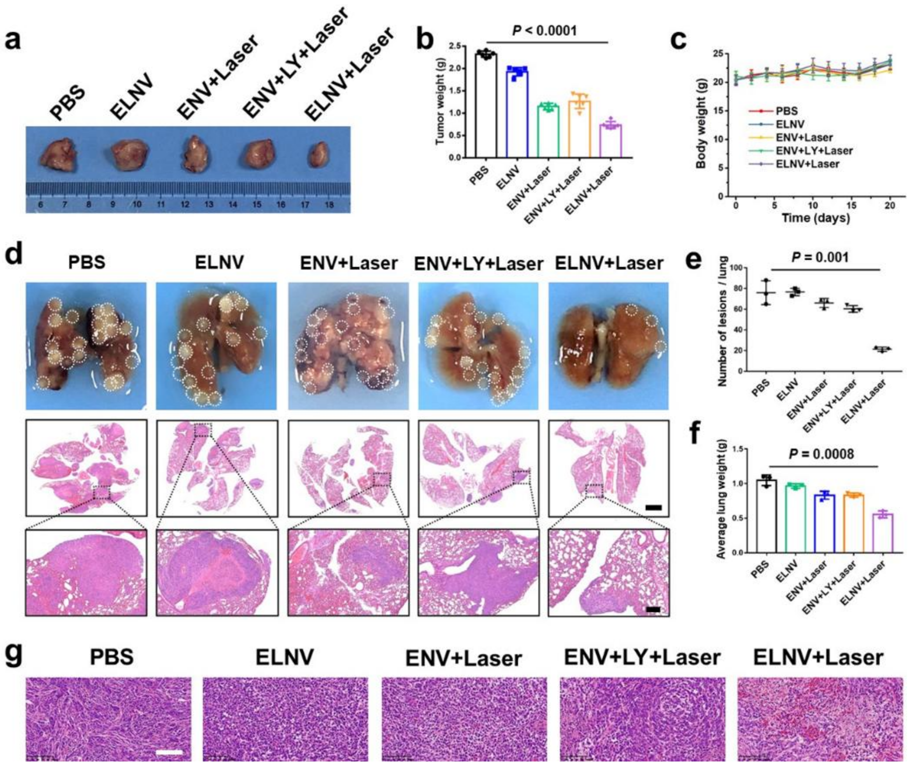

Supplementary Figure 32. Therapeutic efficacy of ELNV nanovesicles *in vivo*. (**a**) Typical photographs of excised tumors from different conditions. (**b**) The average weight of tumors in mice after treatments (n = 6 mice, $P = 5.01\times10^{-12}$). (**c**) Averaged body weight of mice under different conditions n = 6 mice). (**d**) Representative images of the lung tissue and hematoxylin and eosin (H&E) staining of the pulmonary metastasis nodules after different treatments for each group (day 20). Scale bar = 2 mm and 200 µm (enlarged image). (**e**) The number of lung tumors per mouse was scored and averaged for each treatment group (n = 3 mice). (**f**) Average lung weight of each group (n = 3 mice). (**g**) H&E-stained tumor slices from different groups as indicated (*n* = 3 mice, scale bar = 100 µm). The experiment was repeated independently 3 times with similar results. All data are presented as mean ± SD. *P* values derived from the Student's *t*-test (two-tailed, two-sample unequal variance).

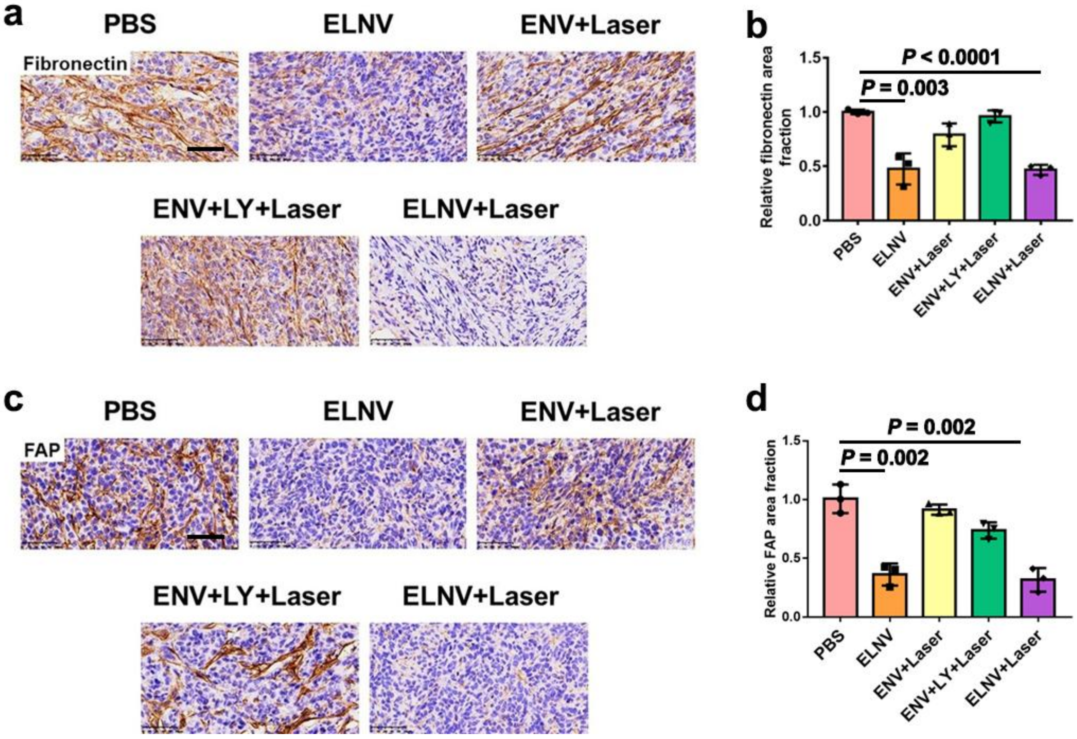

Supplementary Figure 33. Fibronectin and FAP expression in 4T1-tumors *in vivo* after different treatments. (**a** and **c**) Immunohistochemical staining of fibronectin (**a**) and FAP (**c**) expression at the end of antitumor study (scale bars = 50 μm). (**b** and **d**) Semi-quantitative of fibronectin (**b**, $P = 3.28\times10^{-3}$ and 5.79×10^{-5}) and FAP (**d**) area by Image J. All data are presented as mean ± SD. n = 3 mice. P values derived from the Student's t-test (two-tailed, two-sample unequal variance).

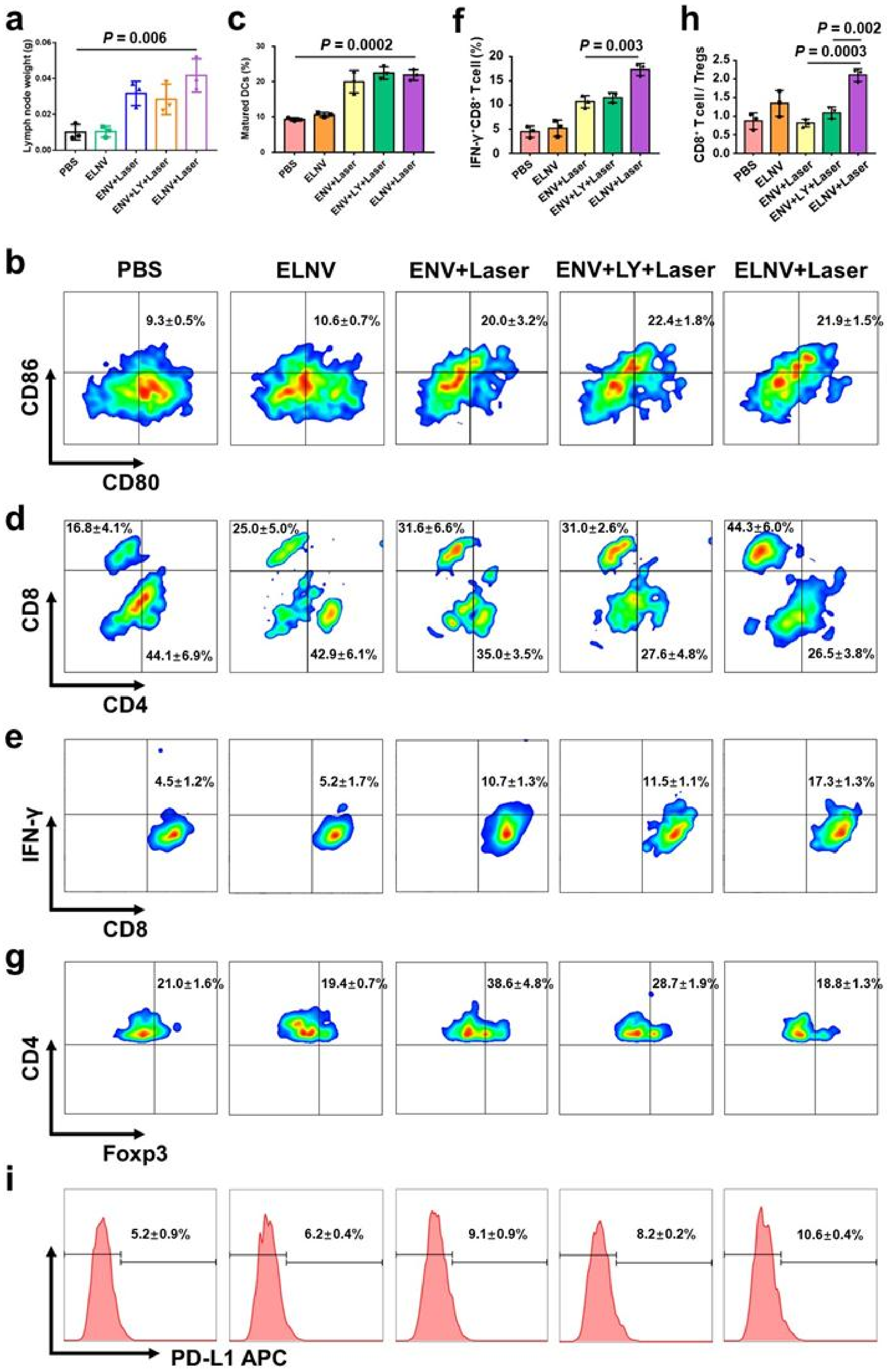

Supplementary Figure 34. Immunologic evaluation of ELNV nanovesicle-based immunotherapy of 4T1

tumor. (**a**) The weight of lymph nodes of 4T1 tumor-bearing mice at the end of treatments. (**b** and **c**) The frequency of maturated DCs (CD11c$^+$CD80$^+$CD86$^+$) in the tumor-draining LNs of 4T1 tumor. (**d**) Flow cytometric quantification of intratumoral infiltration of CD4$^+$ and CD8$^+$ T cells in 4T1 tumor model. (**e-g**) Flow cytometric quantification of (**e** and **f**) intratumoral infiltration of IFN-γ$^+$CD8$^+$ T cells (CD3$^+$CD8$^+$IFN-γ$^+$) and (**g**) Tregs (CD3$^+$CD4$^+$Foxp3$^+$) in 4T1 tumor model. (**h**) CD8$^+$ T cell-to-Treg ratio in 4T1 tumor-bearing mice. (**i**) Flow cytometric quantification of PD-L1 expression in 4T1 tumor model. All data are presented as mean ± SD. n = 3 mice. *P* values derived from the Student's *t*-test (two-tailed, two-sample unequal variance).

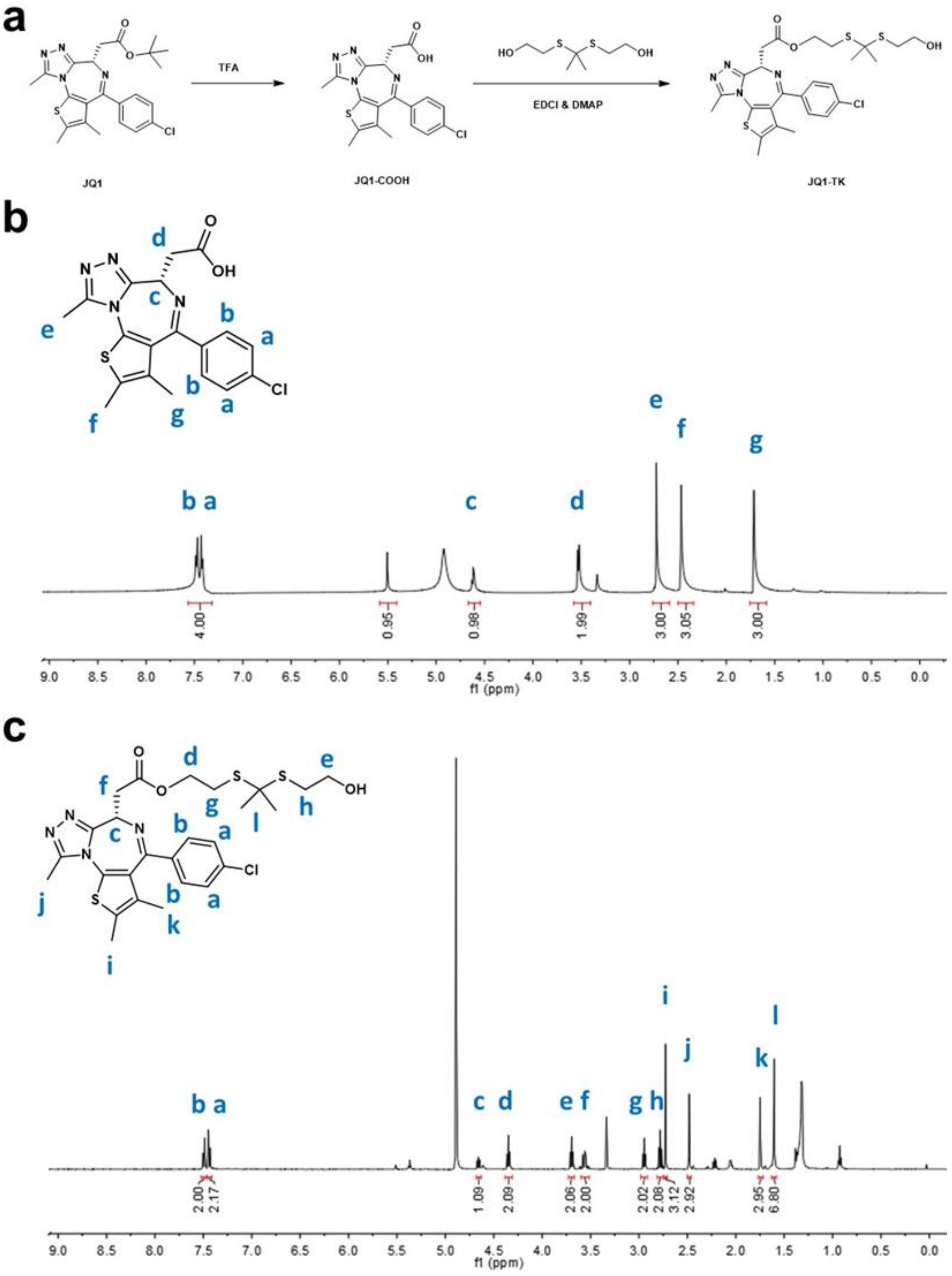

Supplementary Figure 35. Synthesis and characterization of JQ1-TK. (**a**) The synthetic route of JQ1-TK; (**b**) ^{1}H-NMR spectrum of JQ1-COOH. (**c**) ^{1}H-NMR spectrum of JQ1-TK.

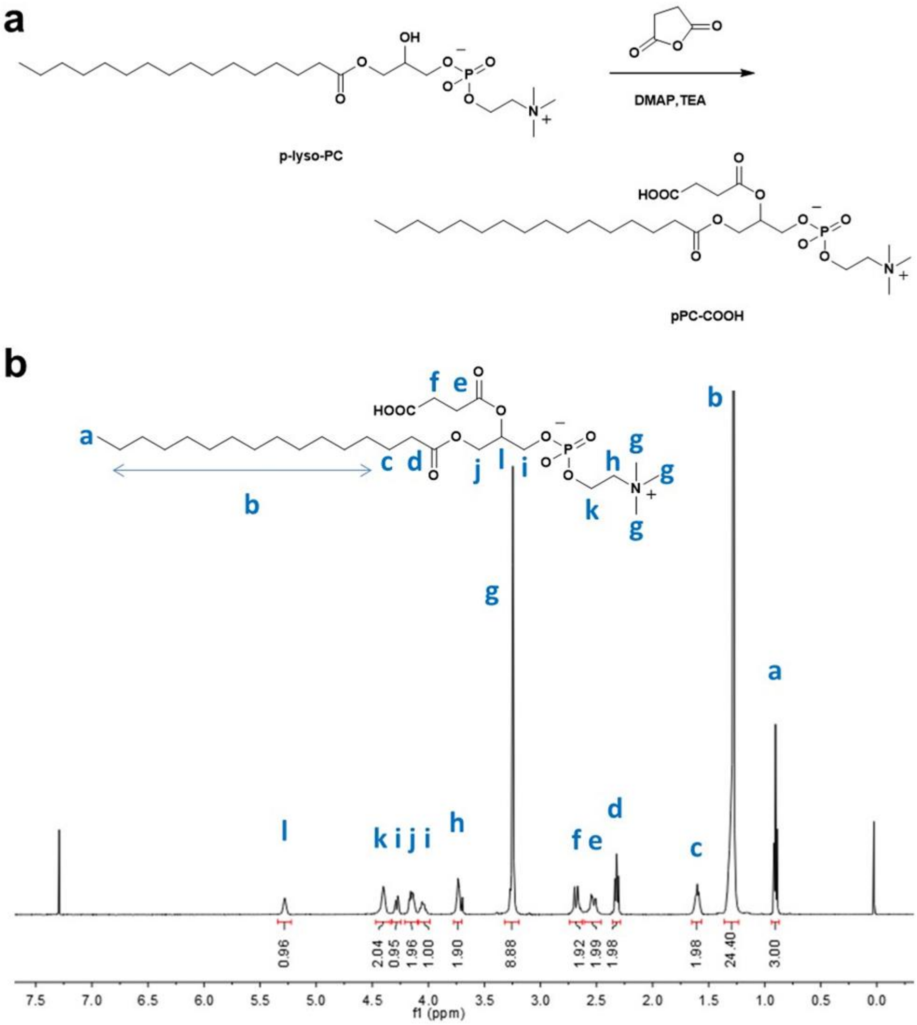

Supplementary Figure 36. Synthesis and characterization of pPC-COOH. (**a**) The synthetic route of pPC-COOH. (**b**) ^{1}H-NMR spectrum of pPC-COOH.

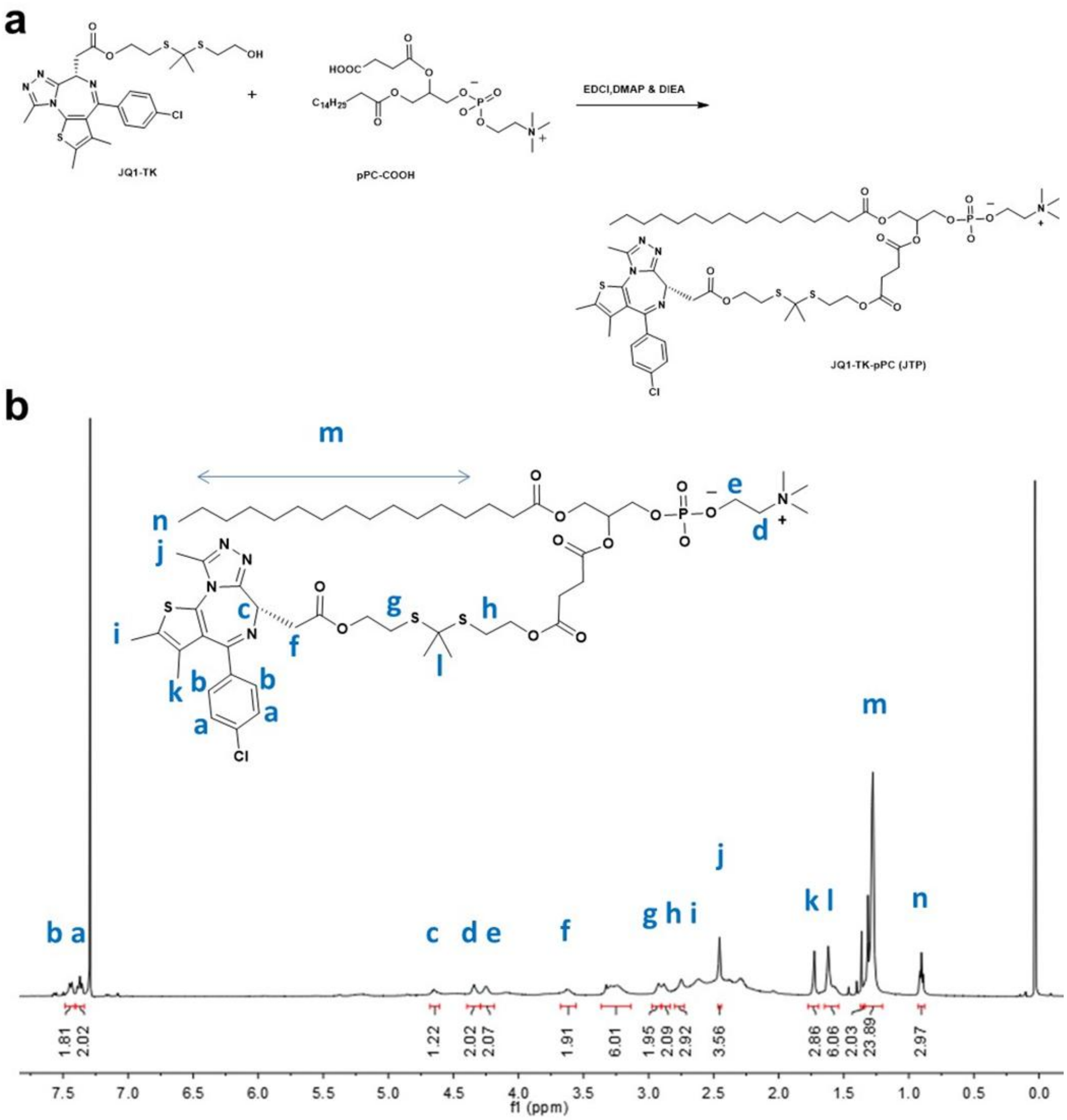

Supplementary Figure 37. Synthesis and characterization of JQ1-TK-pPC (JTP). (**a**) The synthetic route of JQ1-TK-pPC. (**b**) ^{1}H-NMR spectra of JQ1-TK-pPC.

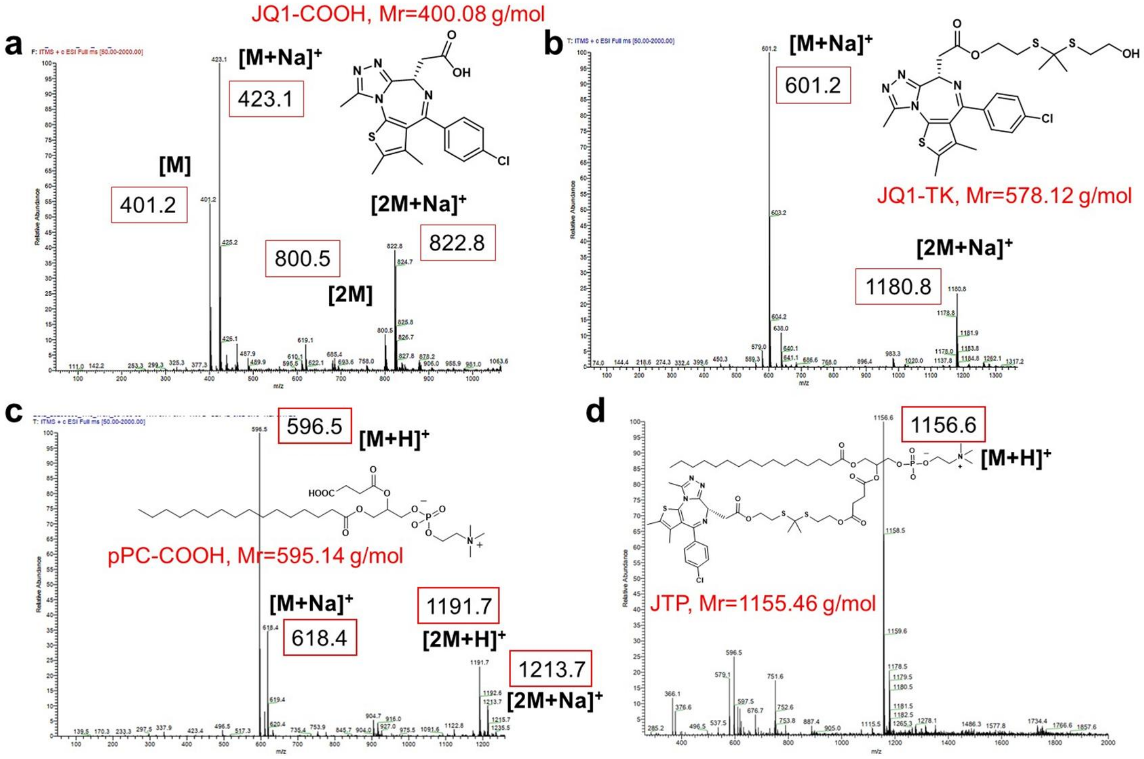

Supplementary Figure 38. (**a-d**) ESI-MS spectra of JQ1-COOH ($C_{19}H_{17}ClN_4O_2S$) (**a**), JQ1-TK ($C_{26}H_{31}ClN_4O_3S_3$) (**b**), pPC-COOH ($C_{28}H_{54}NO_{10}P$) (**c**), and JTP ($C_{54}H_{83}ClN_5O_{12}PS_3$) (**d**).

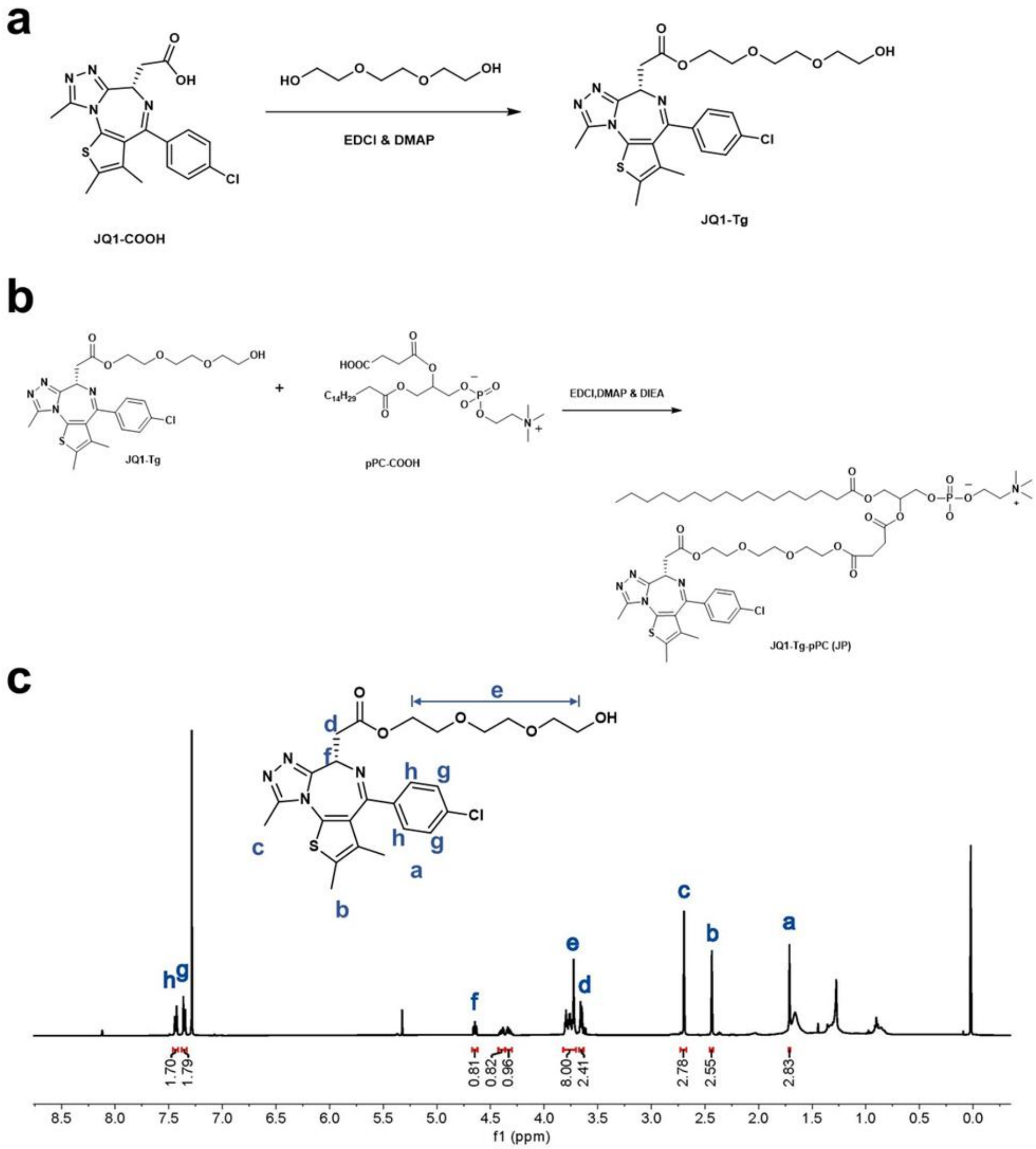

Supplementary Figure 39. (**a-c**) The synthetic route of JQ1-Tg (**a**), and JQ1-Tg-pPC (JP) (**b**), ^{1}H-NMR spectra of JQ1-Tg (**c**).

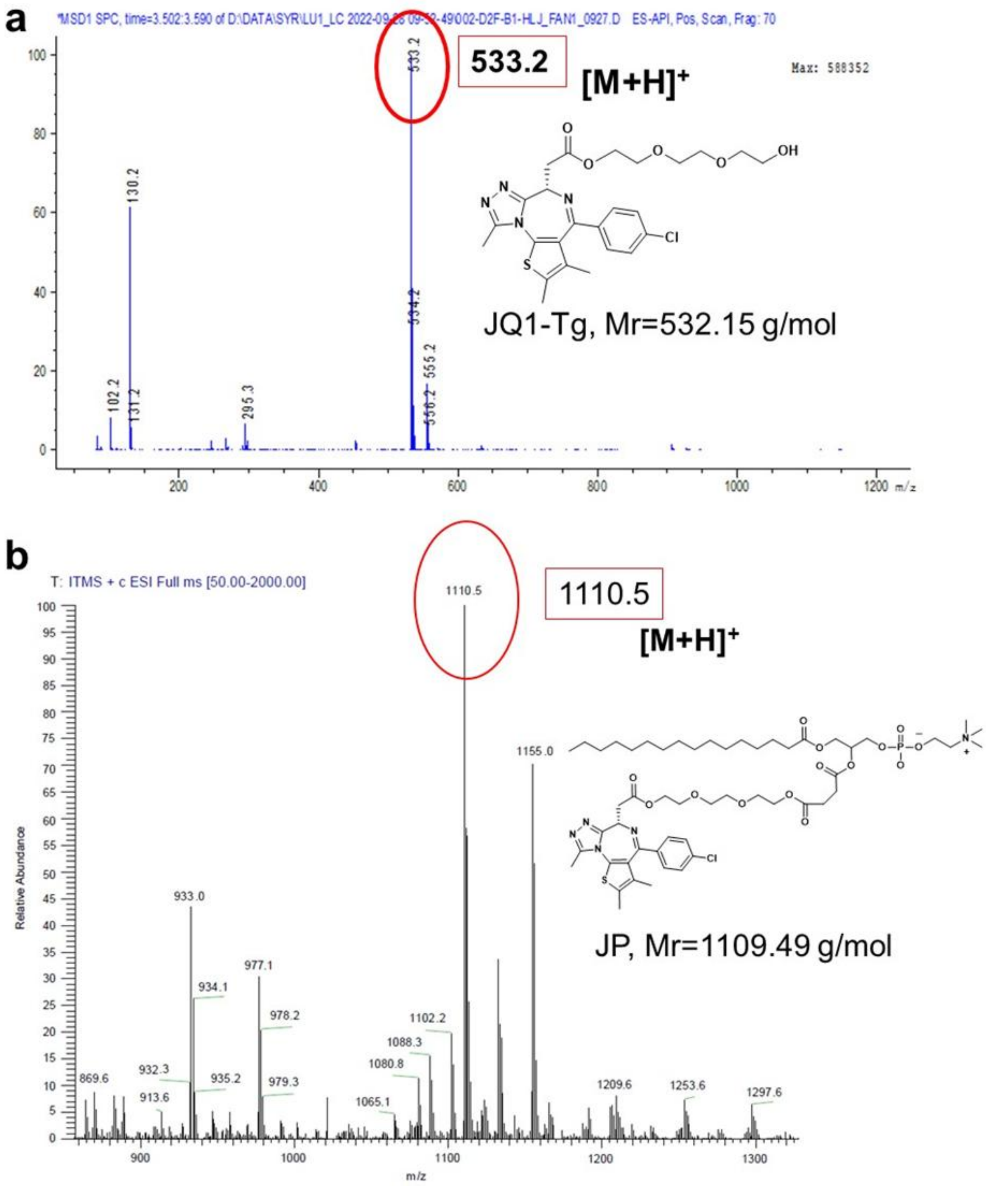

Supplementary Figure 40. (**a** and **b**) Mass spectra of JQ1-Tg (**a**) and JQ1-Tg-pPC (JP) (**b**).

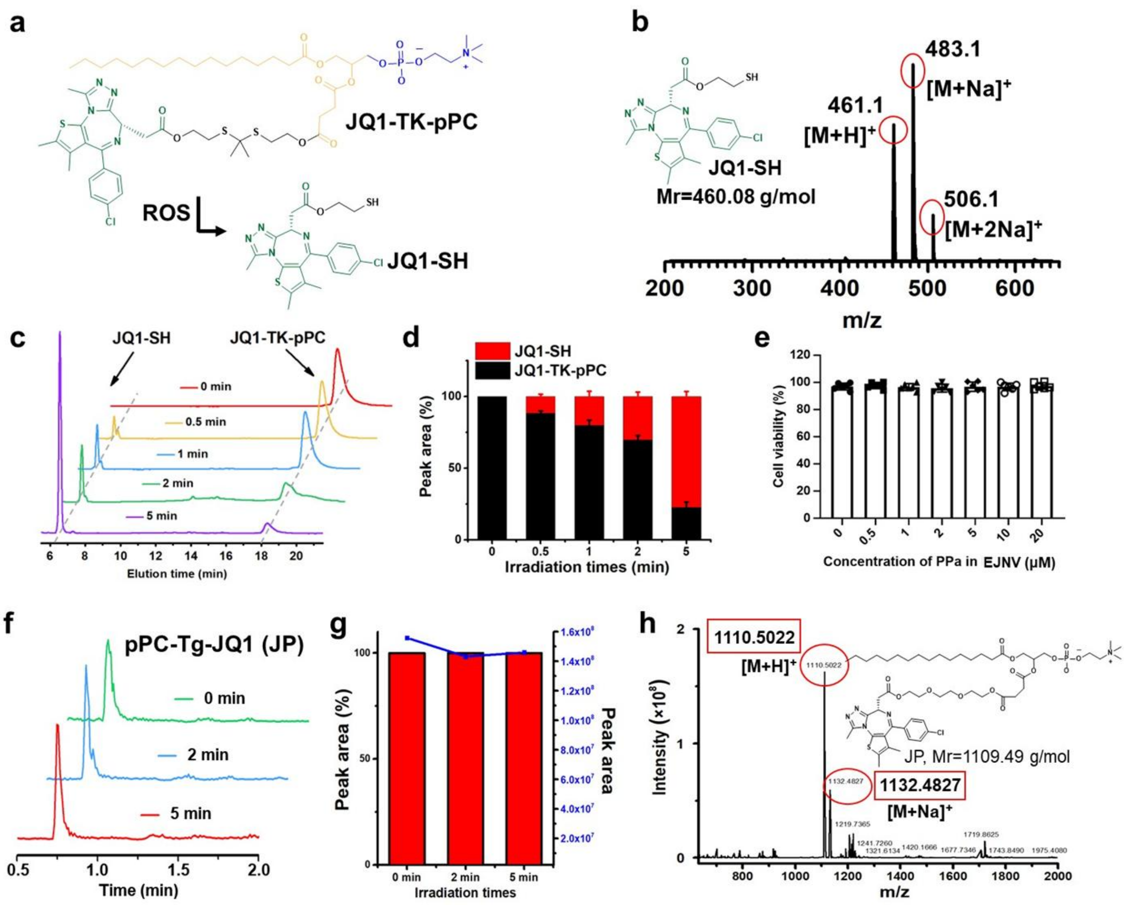

Supplementary Figure 41. ROS-responsive break behavior of pPC-TK-JQ1. (**a**) Reaction mechanism of ROS-triggered activation of the pPC-TK-JQ1 prodrug. (**b**) ESI-MS spectra of JQ1-SH release from JTP. (**c**) Time course total ions chromatogram (TIC) curves of pPC-TK-JQ1 activation kinetics. (**d**) PDT-induced JQ1 release profile of the pPC-TK-JQ1 prodrug (mean ± SD, n = 3 independent experiments). (**e**) Cell viability of EJNV in 4T1 cells *in vitro* (mean ± SD, n = 6 biologically independent cells). (**f**) Time course total ions chromatogram (TIC) curves of pPC-Tg-JQ1 (JP) inactivation kinetics. (**g**) Peak area and percent peak area of JP from (**f**). (**h**) ESI-MS spectra of JP at 5 min after PDT treatment.

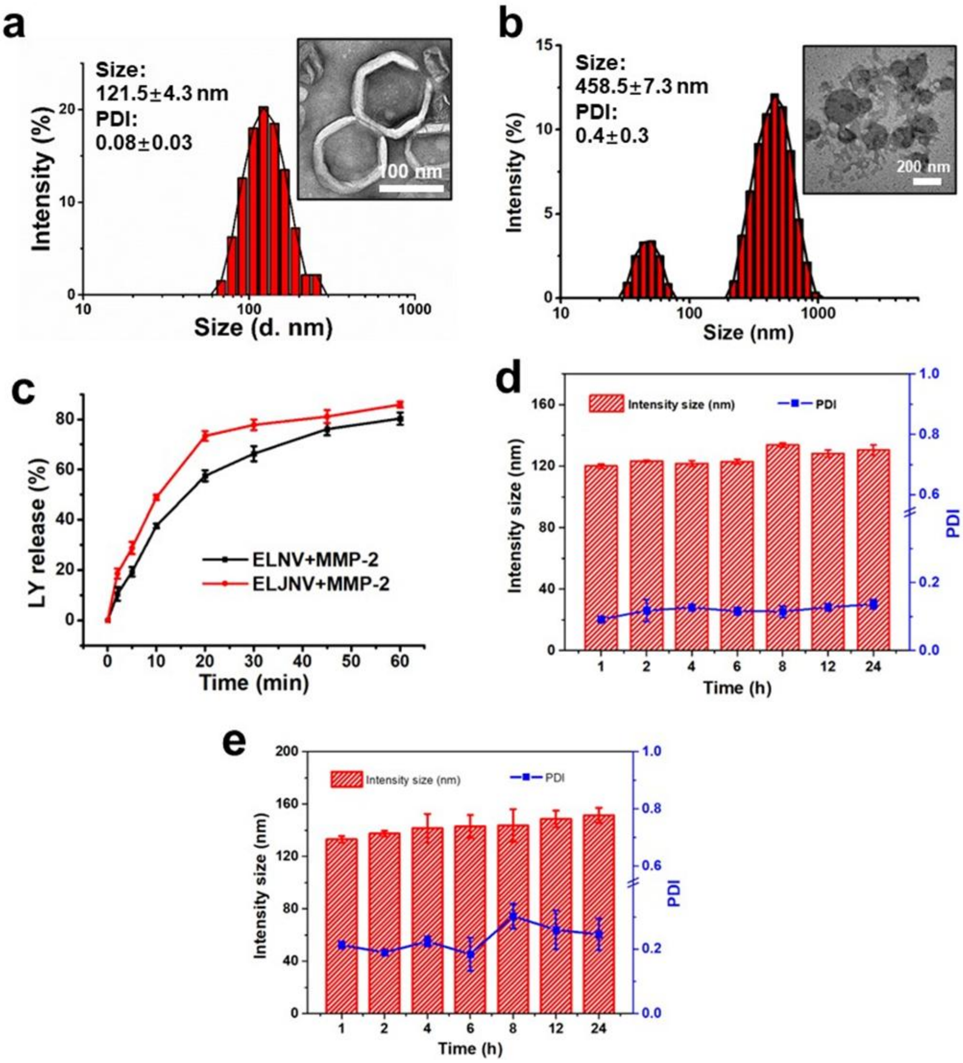

Supplementary Figure 42. Construction of ELJNV nanovesicles. (**a**) Hydrodynamic diameters and the representative TEM images of ELJNV. (**b**) Hydrodynamic diameters and the representative TEM images of ELJNV upon 4 h incubation with MMP-2 (40 μg/mL). (**c**) LY release profiles of ELNV and ELJNV nanovesicles in the presence of MMP-2 (40 μg/mL) (n = 3 independent experiments). (**d**) DLS-determined colloidal stability of ELJNV nanovesicle in 10% FBS-containing phosphate buffer solution (n = 3 independent experiments). (**e**) DLS-determined stability of the ELJNV nanovesicle in whole blood serum of mouse (n = 3 independent experiments). All data are presented as mean ± SD.

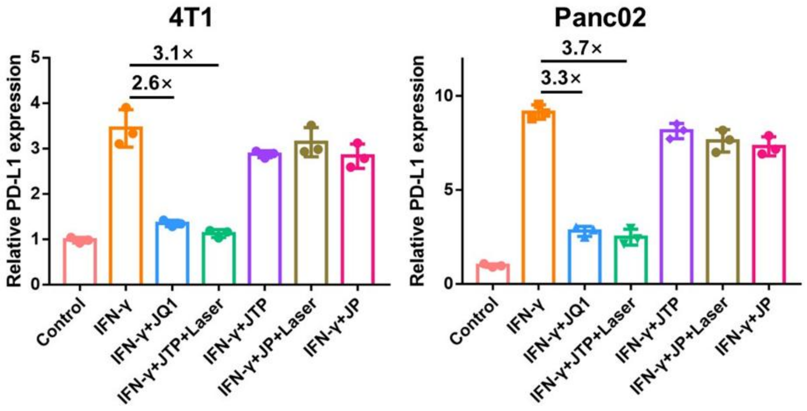

Supplementary Figure 43. PD-L1 expression of flow cytometry data of relative PD-L1 expression on the surface of 4T1 or Panc02 tumor cells upon different treatment conditions *in vitro* (mean ± SD, n = 3 biologically independent cells).

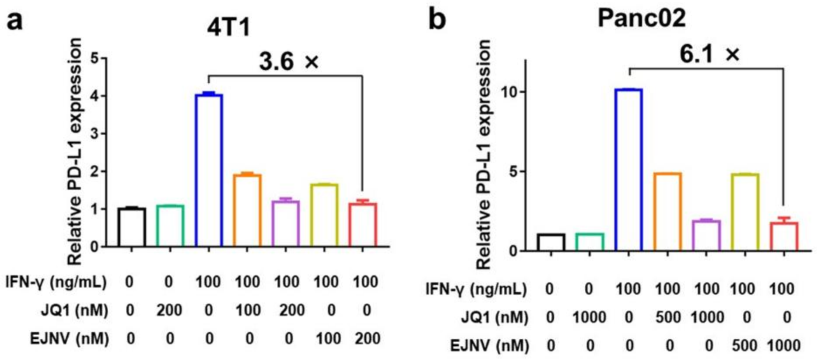

Supplementary Figure 44. EJNV nanovesicles block IFN-γ induced PD-L1 expression *in vitro*. Flow cytometry data of relative PD-L1 expression on the surface of **a** 4T1 or **b** Panc02 tumor cells upon different treatments *in vitro* (mean ± SD, n = 3 biologically independent cells).

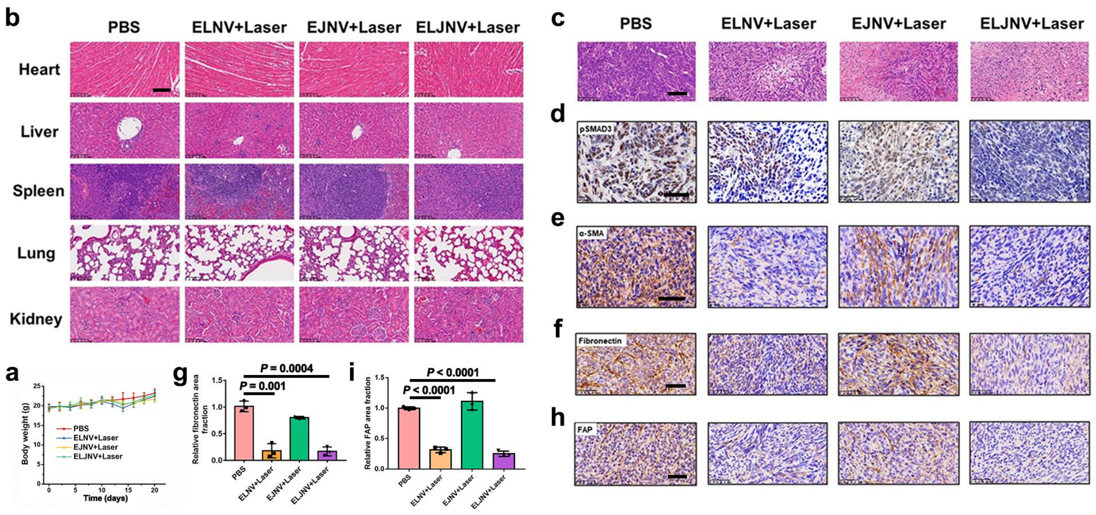

Supplementary Figure 45. H&E and immunohistochemical staining images of normal or tumor tissues after different treatments. (**a**) Averaged body weight of mice under different conditions (mean ± SD, n = 6 mice). (**b**) H&E-stained sections of the heart, liver, spleen, lung, and kidney of 4T1 tumor-bearing mice with different treatments (*n* = 3 mice, Scale bar = 100 μm). (**c-f**) H&E staining images (**c**), the immunohistochemical staining of pSmad3 (**d**), α-SMA (**e**), and fibronectin (**f**) of the excised tumors from each group after the treatments, respectively. Scale bars = 100 μm (**c**), Scale bars = 50 μm (**d-f**) (*n* = 3 mice). (**g**) Semi-quantitative of fibronectin area by image J. (mean ± SD, n = 3 mice). (**h**) The immunohistochemical staining of FAP of the excised tumors from each group after the treatments. Scale bars = 50 μm (*n* = 3 mice). (**i**) Semi-quantitative of FAP area by image J (mean ± SD, n = 3 mice, $P = 2.28\times10^{-5}$ and 1.28×10^{-5}). *P* values derived from the Student's *t*-test (two-tailed, two-sample unequal variance). The experiment was repeated independently 3 times with similar results in **b-f**, **h**.

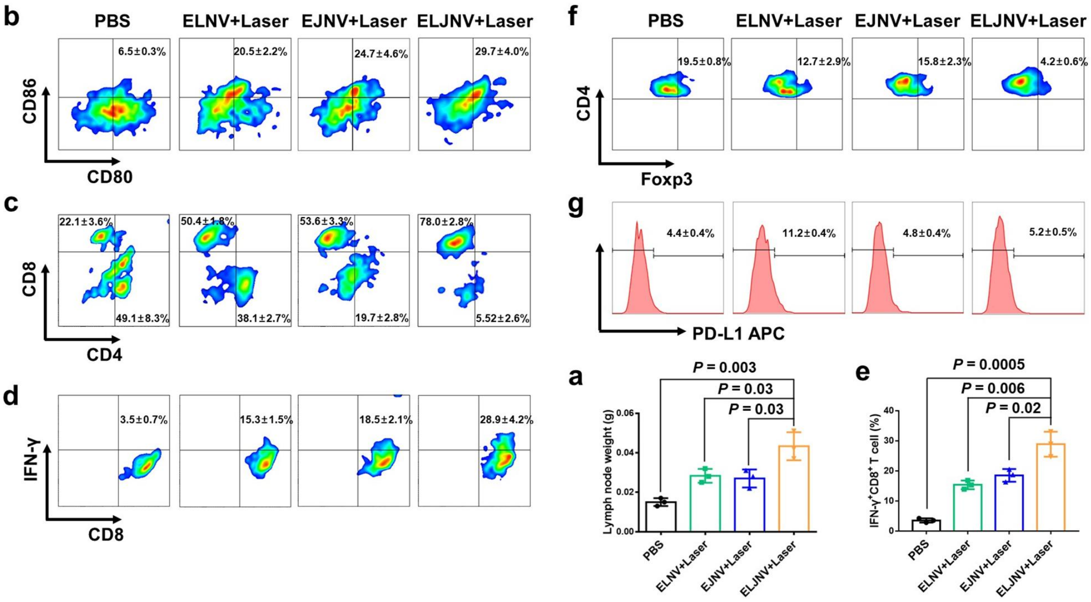

Supplementary Figure 46. Immunologic evaluations after different treatments. (**a**) The weight of lymph nodes of 4T1 tumor-bearing mice at the end of treatments (mean ± SD, n = 3 mice). (**b**) The frequency of maturated DCs ($CD11c^+CD80^+CD86^+$) in the tumor-draining LNs of 4T1 tumor (mean ± SD, n = 3). (**c**) Flow cytometric quantification of intratumoral infiltration of $CD4^+$ and $CD8^+$ T cells in 4T1 tumor model (mean ± SD, n = 3). (**d-f**) Flow cytometric quantification of (**d** and **e**) intratumoral infiltration of IFN-γ^+CD8^+ T cells ($CD3^+CD8^+$IFN-γ^+), and (**f**) Tregs ($CD3^+CD4^+Foxp3^+$) in 4T1 tumor model (mean ± SD, n = 3 mice). (**g**) Flow cytometric quantification of PD-L1 expression in 4T1 tumor model. *P* values derived from the Student's *t*-test (two-tailed, two-sample unequal variance).

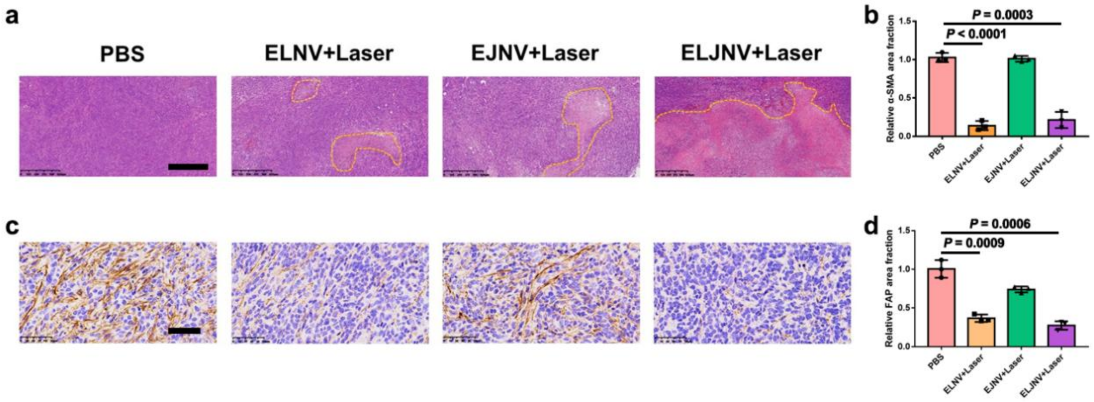

Supplementary Figure 47. H&E staining images of tumor tissues and FAP expression of tumor tissues after different treatments. (**a**) H&E staining of the tumor slices from different groups as indicated (Scale bars = 625 μm). Yellow circles highlight the regions of tumor necrotic. (**b**) Semi-quantitative of α-SMA area in Fig. 6**i** by Image J (mean ± SD, n = 3 mice, P = 5.60×10^{-5} and 3.06×10^{-4}). (**c**) The immunohistochemical staining of FAP of the excised tumors from each group after the treatments. Scale bars = 50 μm. (**d**) Semi-quantitative of FAP area by image J (mean ± SD, n = 3 mice). P values derived from the Student's t-test (two-tailed, two-sample unequal variance).

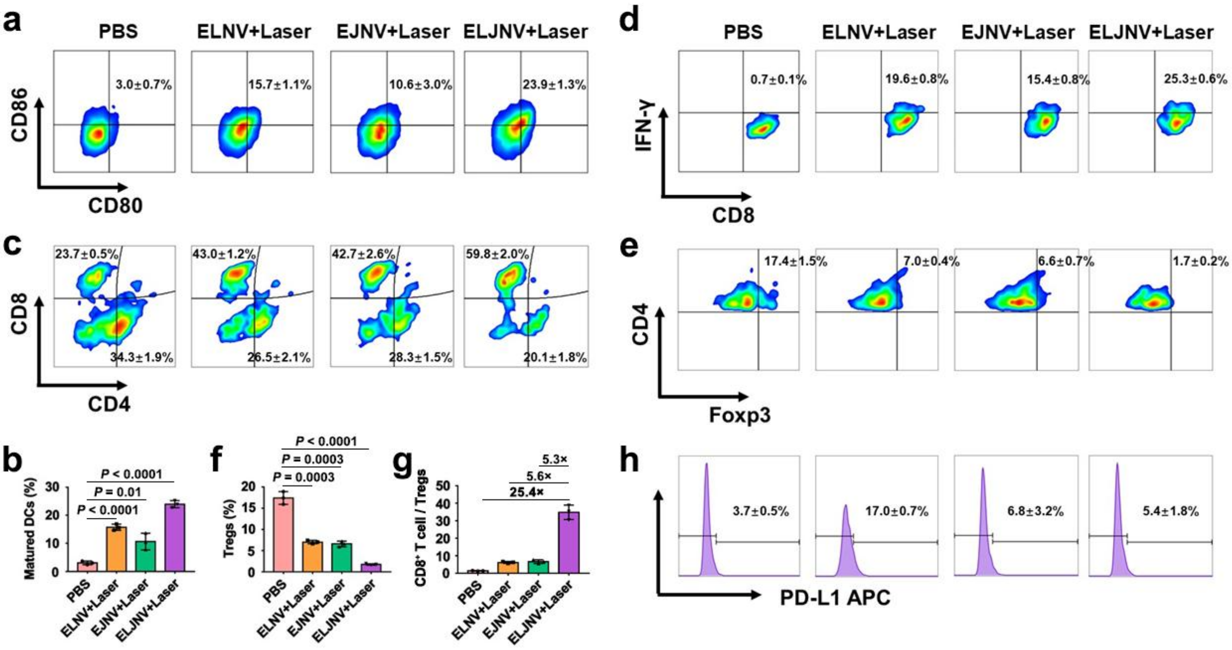

Supplementary Figure 48. Immunologic evaluation after different treatments. (**a** and **b**) The frequency of maturated DCs ($CD11c^+CD80^+CD86^+$) in the tumor-draining LNs of Panc02 tumor ($P = 6.79\times10^{-5}$, 1.22×10^{-2} and 1.37×10^{-5}). (**c**) Flow cytometric quantification of tumor-infiltrating $CD4^+$ and $CD8^+$ T cells in Panc02 tumor model. (**d-f**) Flow cytometric quantification of (**d**) tumor-infiltrating IFN-γ^+CD8^+ T cells ($CD3^+CD8^+$IFN-γ^+), and (**e** and **f**) Tregs ($CD3^+CD4^+Foxp3^+$) in the Panc02 tumor model *in vivo* ($P = 3.15\times10^{-4}$, 3.29×10^{-4} and 5.60×10^{-5}). (**g**) $CD8^+$ T cell-to-Treg ratio in Panc02 tumor-bearing mice. (**h**) Flow cytometric quantification of PD-L1 expression on the surface of the Panc02 tumor cells *in vivo*. All data are presented as mean ± SD. n = 3 mice. *P* values derived from the Student's *t*-test (two-tailed, two-sample unequal variance).

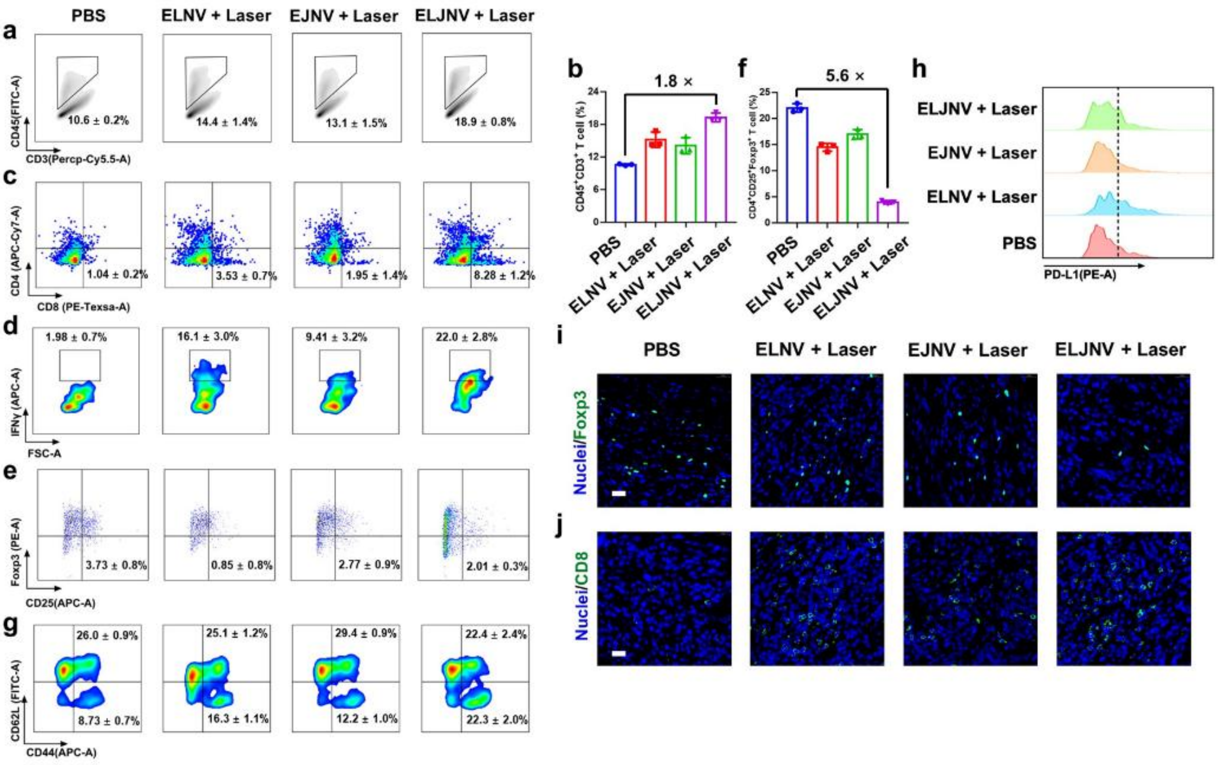

Supplementary Figure 49. (**a** and **b**) Representative flow cytometry plots (**a**), and quantification of tumor-infiltrating $CD3^+$ T cells ($CD45^+CD3^+$) (**b**) in KPC tumor model. (**c**) Flow cytometry plots of $CD4^+$ T cells ($CD45^+CD3^+CD4^+$) and $CD8^+$ T cells ($CD45^+CD3^+CD8^+$). (**d**) Representative flow cytometry plots of IFN-γ^+CD8^+ T cells ($CD45^+CD3^+CD8^+$IFN-γ^+). (**e** and **f**) Representative flow cytometry plots (**e**), and quantification of tumor-infiltrating Tregs ($CD45^+CD3^+CD4^+CD25^+Foxp3^+$) (**f**) in KPC tumor model. (**g**) Flow cytometric quantification of T_{CM} ($CD45^+CD3^+CD8^+CD44^+CD62L^+$) and T_{EM} ($CD45^+CD3^+CD8^+CD44^+CD62L^-$) in the spleens of KPC tumor-bearing C57BL/6 mice post-21-days treatment. (**h**) Flow cytometry analysis of intratumoral PD-L1 expression in KPC tumors after different treatments. (**i** and **j**) IF staining of tumor-infiltrating Tregs (**i**), and $CD8^+$ T cells (**j**) in KPC tumor sections upon different treatments. Scale bar = 20 μm. All data are presented as mean ± SD. n = 3 mice.

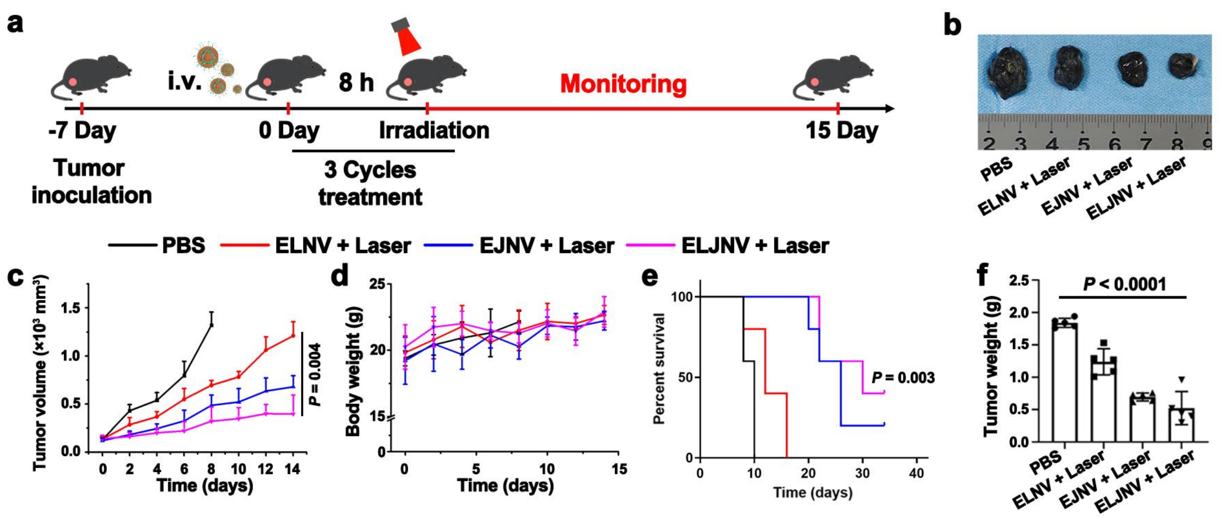

Supplementary Figure 50. (**a**) Treatment schedule of ELJNV-mediated combination immunotherapy of B16-F10 melanoma tumor model. (**b**) Representative photographs of tumor tissues at the end of the antitumor study. (**c**) Growth profiles of B16-F10 tumor upon different treatments. (**d**) Body weight change curves, and (**e**) Survival curves of B16-F10 tumor-bearing mice upon different treatments. (**f**) Averaged tumor weight was examined at the end of the antitumor study ($P = 3.95\times10^{-6}$). All data are presented as mean ± SD. n = 5 mice. *P* value derived from the Student's *t*-test (two-tailed, two-sample unequal variance).

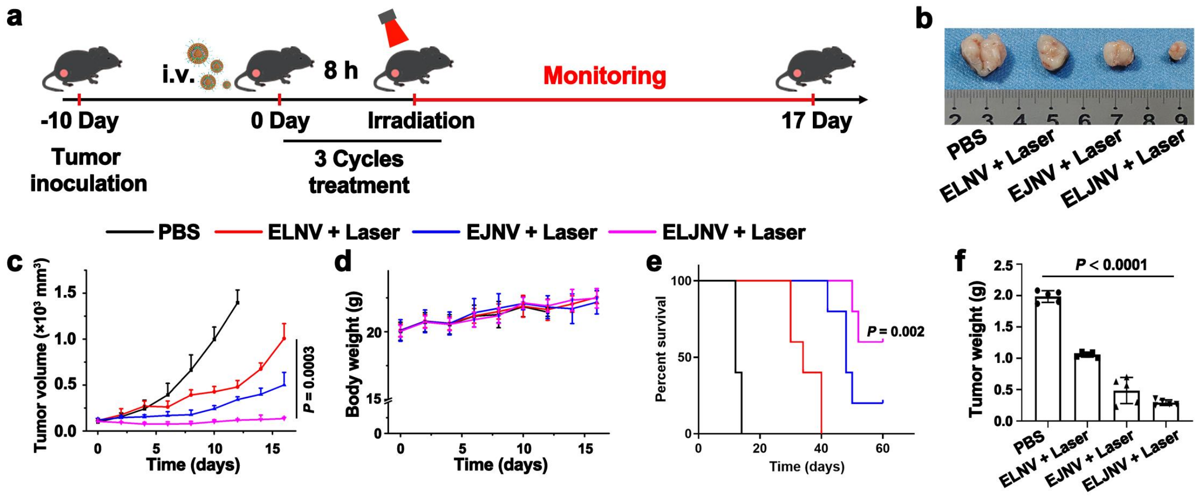

Supplementary Figure 51. (**a**) Treatment schedule of ELJNV-mediated combination immunotherapy of MC38 colorectal tumor model. (**b**) Representative photographs of MC38 tumors were collected at the end of the antitumor study. (**c**) Growth profiles of MC38 tumor upon different treatments. (**d**) Body weight change curves, and (**e**) Survival curves of MC38 tumor-bearing mice upon different treatments. (**f**) Averaged tumor weight was examined at the end of the antitumor study ($P = 3.53\times10^{-10}$). All data are presented as mean ± SD. n = 5 mice. *P* value derived from the Student's *t*-test (two-tailed, two-sample unequal variance).

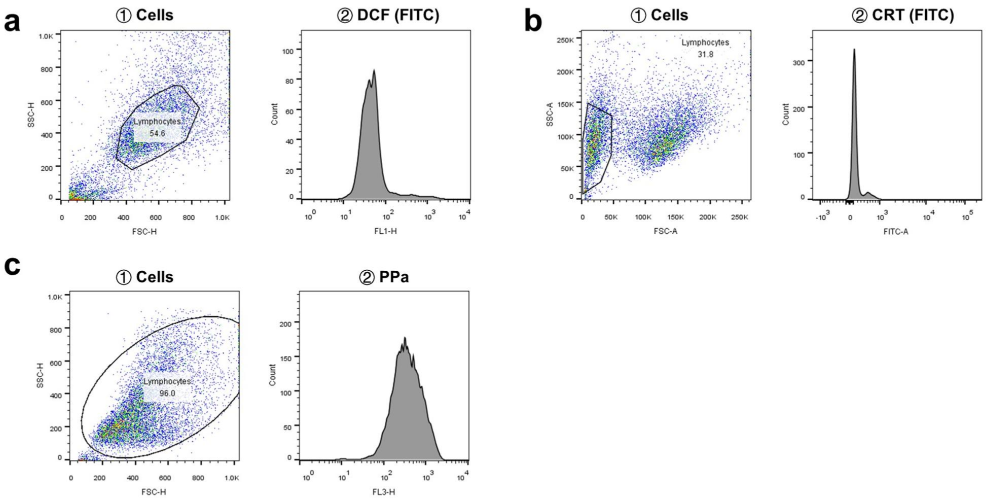

Supplementary Figure 52. The gating strategies of flow cytometry analyses for (**a**) ROS generation (related to Fig. 3**h**, Supplementary Figs. 24**b**, **c** and **25**); (**b**) CRT positive cells (related to Fig. 3**i**, Supplementary Figs. 26**b**, 27**b**, **d**) and (**c**) cellular uptake (related to Fig. 3**f**).

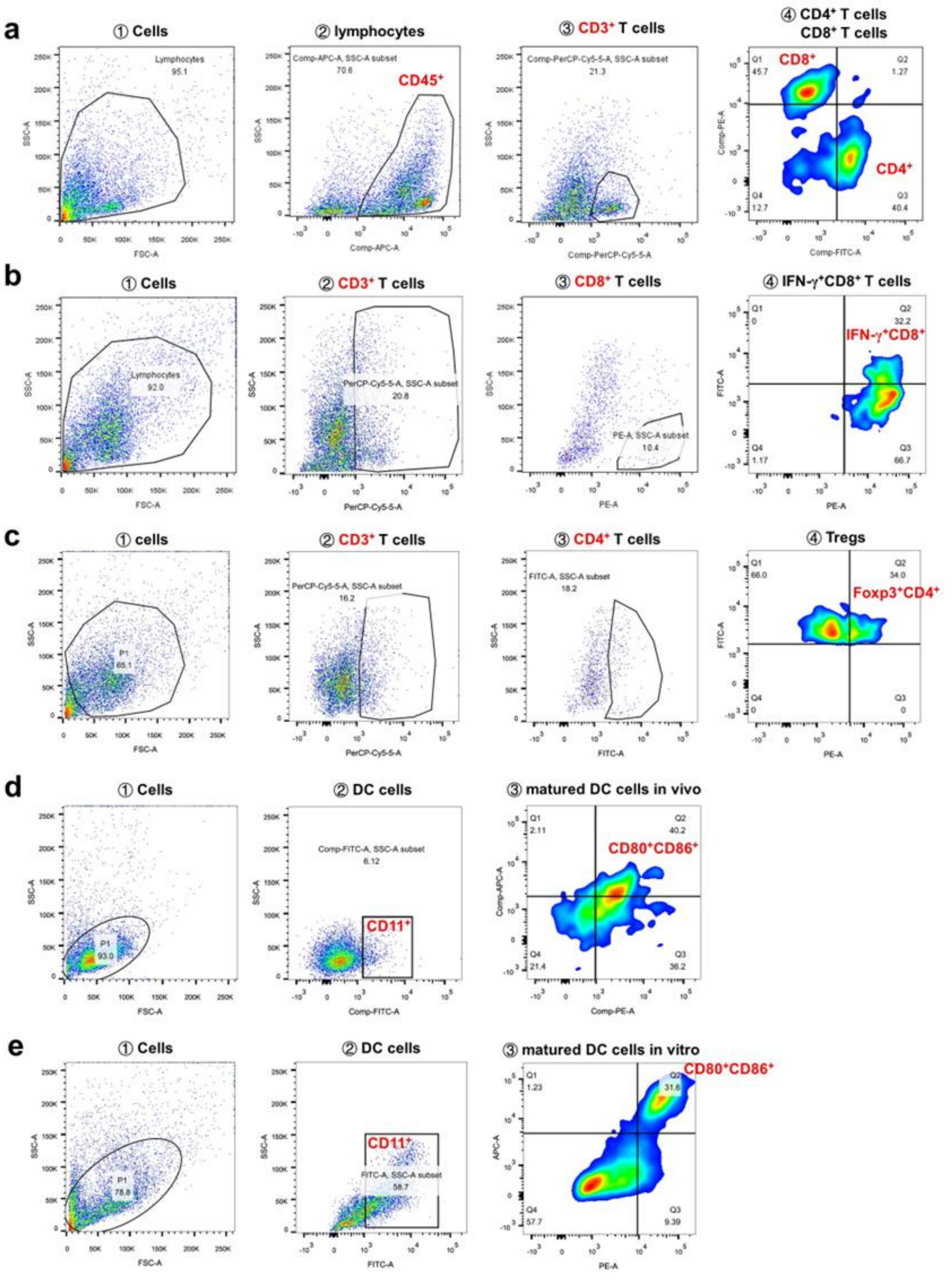

Supplementary Figure 53. The gating strategies of flow cytometry analyses in **Figs. 3k**, **4-6** and **Supplementary Figs. 26c**, **28**, **34**, **46**, **48**. (a) $CD3^+CD4^+CD8^+$ T cells; (b) IFN-γ^+CD8^+ T cells; (c) Tregs; (d) matured DCs in vivo; (e) matured DCs in vitro.

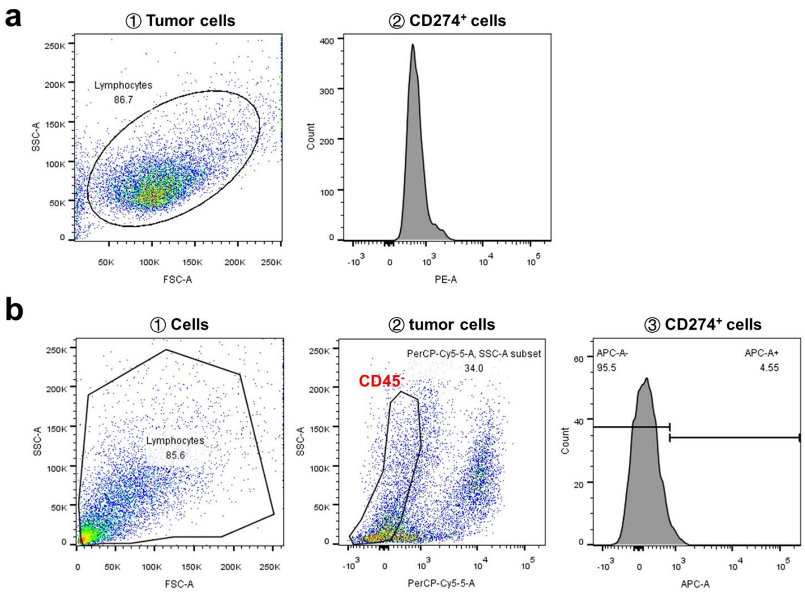

Supplementary Figure 54. The gating strategies of flow cytometry analyses for PD-L1 expression on the surface of tumor cells (**a**) in vitro (related to Supplementary Figs. **43**, **44**) and (**b**) in vivo (related to **Figs. 4-6** and **Supplementary Figs. 34**, **46**, **48**).

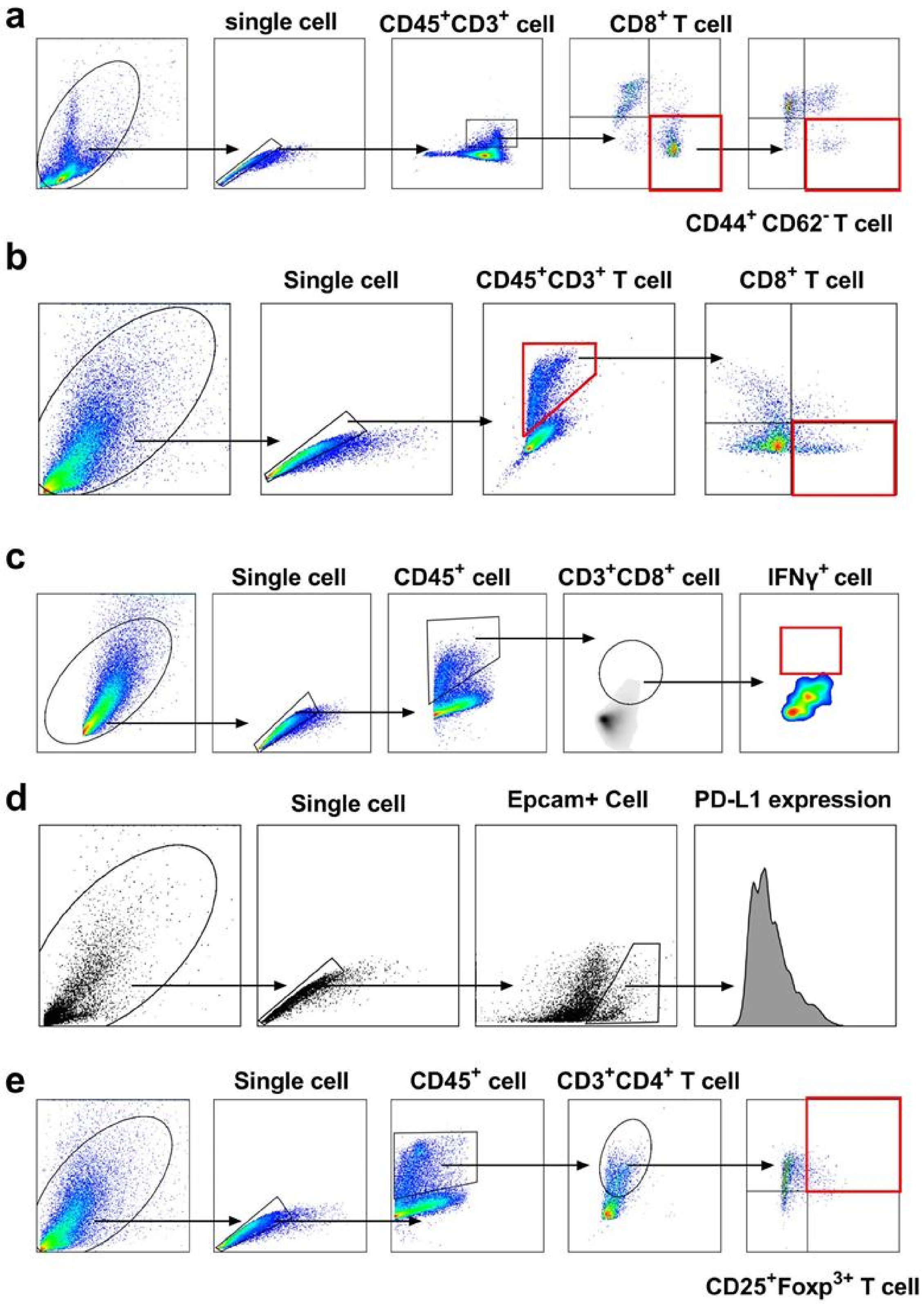

Supplementary Figure 55. The gating strategies of flow cytometry analyses in **Fig. 7** and **Supplementary Fig. 49**. (**a**) $CD62L^{-}CD44^{+}$ effector memory $CD8^{+}$ T cells in the spleens of mice; (**b**) $CD3^{+}CD4^{+}CD8^{+}$ T cells; (**c**) IFN-$\gamma^{+}CD8^{+}$ T cells; (**d**) PD-$L1^{+}$ tumor cells; (**e**) Tregs.

Supplementary Table 1. A list of abbreviations used in the context.

Abbreviation	Full name	Abbreviation	Full name
α-SMA	alpha-smooth muscle actin	AUC	area under the time-concentration curve
BCA	Bicinchoninic Acid	BMDCs	bone marrow dendritic cells
BRD4	Bromodomain-containing protein 4	CAFs	Cancer-associated fibroblasts
CAR-T	chimeric antigen receptor T-cell immunotherapy	CLSM	Confocal laser-scanning microscopy
CRT	calreticulin	CTLs	Cytotoxic T lymphocytes
CVF	collagen volume fraction	DAPI	4’,6-diamidino-2-phenylindole dihydrochloride
DCs	Dendritic cells	DCFH-DA	2’,7’-dichlorofluorescein diacetate
DCM	dichloromethane	DIEA	N,N-Diisopropylethylamine
DL%	drug loading ratio	DLS	dynamic light scattering
DMAP	4-dimethylaminopyridine	DMEM	Dulbecco’s modified Eagle’s medium
DMF	N,N-dimethylformamide	DMSO	dimethyl sulfoxide
DPPC	1,2-dipalmitoyl-sn-glycero-3-phosphocholine	DSPC	1,2-dioctadecanoyl-sn-glycero-3-phosphocholine
ECM	Extracellular matrix	EDCI	N-(3-(dimethylamino)-propyl)-N-ethylcarbodiimide hydrochloride
EE%	encapsulation efficiency	ELISA	enzyme-linked immunosorbent assay
ELJNV	LYand JQ1 co-loaded enzyme-sensitive nanovesicles	ELNV/LNV	LY-loaded enzyme-sensitive nanovesicles/their enzyme-insensitive analogs
Em	Emission	ENV	ELNV nanovesicles without LY
Ex	Excitation	FAP	fibroblast activation protein
FBS	fetal bovine serum	FI	Fluorescence imaging
Fmoc-GPLGLAG	Fmoc-protected heptapeptide Gly-Pro-Leu-Gly-Leu-Ala-Gly	$GdCl_3$	Gadolinium chloride
HMGB1	high mobility group protein B1	HOBT	1-hydroxybenzotriazole
HPLC	High-performance liquid chromatography	H&E	Hematoxylin and eosin
ICB	immune-checkpoint blockade	ICD	immunogenic cell death
ICP-MS	inductively coupled plasma mass spectrometry	IDO	indoleamine 2,3-dioxygenase
IETs	Immune-excluded tumors	IF	immunofluorescence
IFN-γ	interferon-gamma	IHC-P	Immunohistochemistry (Paraffin)
Int.	intensity	i.t.	intratumoral injection
ITM	immunosuppressive tumor microenvironment	i.v.	tail vein injection
JAK/STAT	Janus kinase/signal transducer and activator of transcription	JP	JQ1- triethylene glycol (Tg)-pPC

JQ1	(S)-tert-butyl 2-(4-(4-chlorophenyl)-2,3,9-triMethyl-6H-thieno[3,2-f][1, 2,4]triazolo[4,3-a][1,4]diazepin-6-yl)acetate	JTP	JQ1-thioketal (TK)-pPC
LC-MS	liquid chromatograph mass spectrometer	LJNV	enzyme-insensitive nanovesicles JNV with LY
LNs	lymph nodes	LNV	enzyme-insensitive nanovesicles
LR-ESI-MS	Low-resolution electrospray ionization mass spectrometry	LY	LY2157299
MALDI-TOF MS	matrix-assisted laser desorption/ionization time-of-flight mass spectrometry	MMP-2	Matrix metallopeptidase-2
$mPEG_{5k}$-NH_2	Methoxypoly(ethylene glycol) amine	MRI	magnetic resonance imaging
MSOT	multispectral optoacoustic tomography	MWCO	Molecular Weight Cut Off
NMR	nuclear magnetic resonance	O.C.T.	Optimal Cutting Temperature
PAI	photoacoustic imaging	PDAC	pancreatic ductal adenocarcinoma
PD-L1	programmed death ligand 1	PDI	Polydispersity Index
PDT	Photodynamic therapy	PEG	poly(ethylene glycol)
p-lysoPC	1-palmitoyl-2-hydroxy-sn-glycero-3-phosphocholine	PPa	Pyropheophorbide a
pSmad2/3	phospho-small mothers against decapentaplegic 2/3	ROS	reactive oxygen species
RT	room time	s.c.	subcutaneously injection
SDS	sodium dodecyl sulfate	SOSG	sodium dodecyl sulfate
TCGA	The Cancer Genome Atlas	TEA	triethylamine
TEM	Transmission electron microscopy	TFA	trifluoroacetic acid
TGF-β	Transforming growth factor-β	TGFR1	TGF-β receptor 1
TGFRs	TGF-β receptors	TIC	total ions chromatogram
TIL	tumor-infiltrating lymphocyte	TPM	Transcripts Per Kilobase Million
TNBC	triple-negative breast cancer	Tregs	regulatory T cells
$t_{1/2\beta}$	blood-elimination half-life	v	Volume
WB	Western blot	$^1O_2$	singlet oxygen

Supplementary Table 2. PPa encapsulation efficiency and loading ratio of ENV and ELNV nanovesicles (mean ± SD, n = 3 independent experiments).

	ENV	ELNV
Encapsulation efficiency	99.5 ± 0.2%	98.4 ± 0.9%
Loading ratio	2.1 ± 0.5%	1.9 ± 0.3%

Supplementary Table 3. The Gd^{3+}encapsulation efficiency and loading ratio of ENV and ELNV nanovesicles (mean ± SD, n = 3 independent experiments).

	ENV	ELNV
Encapsulation efficiency	18.4 ± 0.4%	17.9 ± 0.4%
Loading ratio	1.9 ± 0.1%	1.7 ± 0.1%

Supplementary Table S4. Pharmacokinetic profiles of PPa, LY, LNV, and ELNV in BALB/c mice (mean ± SD, n = 3 mice).

	LY			PPa		
	Free LY	LNV	ELNV	Free PPa	LNV	ELNV
$AUC_{(0\text{-}t)}$ (mg/L*h)	14.17 ± 0.6	420.83 ± 26.7	436.02 ± 9.12	14.33 ± 0.6	182.4 ± 56.84	202.81 ± 16.25
$MRT_{(0\text{-}t)}$ (h)	1.24 ± 0.06	12.39 ± 0.47	12.51 ± 0.46	0.95 ± 0.09	9.16 ± 1.45	9.82 ± 1.3
$t_{1/2\beta}$ (h)	1.63 ± 0.2	16.62 ± 1.41	17.36 ± 2.0	0.86 ± 0.2	9.8 ± 0.61	12.48 ± 3.02

AUC: area under the time-concentration curve; MRT: mean residence time; $t_{1/2\beta}$: blood-elimination half-life.

Supplementary Table 5. The LY encapsulation efficiency and loading ratio of ELNV and ELJNV nanovesicles (mean ± SD, n = 3 independent experiments).

	ELNV	ELJNV
Encapsulation efficiency	94.0 ± 1.2%	95.7 ± 0.5%
Loading ratio	19.1 ± 0.3%	18.8 ± 2.1%

Supplementary Methods

Antibodies used for flow cytometry, western blot, immunofluorescence and immunohistochemistry analysis

Anti-alpha smooth muscle actin antibody [EPR5368] (ab124964) for immunohistochemistry (IHC-P, 1:1000)/immunofluorescence (IF, 1:250)/Western Blot (WB, 1:10000), anti-CD3 antibody [SP162] (ab135372) for IHC-P (1:150), anti-CD31 antibody [RM1006] (ab281583, 1:50) for IHC-Fr, anti-Calreticulin antibody [EPR3924] - ER Marker (ab92516) for flow cytometry (1:50) and IF (1:300), anti-HMGB1 antibody (ab18256) for IF (1 μg/mL) were purchased from Abcam (UK). Recombinant mouse TGF-β1 (HEK293 expressed, HY-P7117) was obtained from MedChemExpress (MCE, Shanghai, China). Recombinant anti-Smad2 (phospho S467) antibody [EPR23681-40] (ab280888, 1:1000), recombinant anti-Smad2 antibody [EP567Y] (ab33875, 1:1000), anti-Smad3 antibody (ab84177, 1:500), recombinant anti-Smad3 (phospho S423 + S425) antibody [EP823Y] (ab52903, 1:2000) for WB or IHC-P (1:100) were obtained from Abcam (UK). Recombinant anti-collagen I antibody [EPR24331-53] (ab270993, 1:500), goat Anti-Rabbit IgG H&L (Alexa Fluor® 488) (ab150077, 1:500) and goat anti-rabbit IgG H&L (Alexa Fluor® 555) (ab150078, 1:500) for IHC-P were purchased from Abcam. Anti-fibronectin antibody (ab2413) for IHC-P (1:100) and WB (1:50) was obtained from Abcam. Anti-fibroblast activation protein alpha antibody (NB110-85534) for WB (1:1000) was obtained from Novus Biologicals. Anti-fibroblast activation protein, alpha antibody (ab218164) for IHC-P (1:200) was obtained from Abcam. Anti-CD4 antibody [EPR19514] (ab183685, 1:1000), anti-CD8 alpha antibody [EPR21769] (ab217344, 1:2000) and Anti-PD-L1 antibody (ab233482, 10 μg/mL) for IHC-P were obtained from Abcam. Anti-CD3-PerCP-Cy5.5 (551163), anti-CD45-APC (559864), anti-CD45-PerCP-Cy5.5 (550994),

anti-CD8-PE (553033), anti-CD4-FITC (553046), anti-IFN-γ-FITC (554411), anti-Foxp3-PE (560414), anti-CD11c-FITC (557400), anti-CD80-PE (553769), anti-CD86-APC (558703), anti-CD274-APC (564715) and anti-CD274-PE (558091) for flow cytometry (1:100) were all purchased from BD Biosciences (Shanghai). Anti-CD45-FITC (BD, 553079), anti-CD3-PerCP-Cy5.5 (BioLegend, 100218), anti-CD4-APC/Cy7 (BD, 552051), anti-CD8-PE (BD, 553032), anti-IFN-γ-APC (BD, 554413), anti-CD25-APC (Invitrogen, 17-0257-42), anti-Foxp3-PE (Invitrogen, 72-5775-40), anti-CD45-PE (Multi Sciences, F2104502), anti-CD8-PE/Texas red (Abcam, ab25294), anti-CD44-APC (BD, 559250), anti-CD62L-FITC (BioLegend, 104405) and anti-CD326 (Ep-CAM)-PE antibody (BioLegend, 118205) were used for flow cytometry (1:100). Anti-CD163 antibody [EPR19518] (ab182422, 1:800), anti-CD68 antibody (Cell Signaling Technology, 97778, 1:400), anti-CD86 antibody (Cell Signaling Technology, 19589, 1:100) and anti-MMP2 antibody (ab86607, 2 μg/mL) for IHC-Fr or IHC-P were purchased from Abcam. All other reagents and solvents were analytical grade and obtained from SinoPharm Chemical Reagent Co., Ltd. (Shanghai, China).

Synthesis of PPa-PEG$_{5k}$

63.84 mg of PPa (0.12 mmol), 34.42 mg of EDCI (0.18 mmol), 24.35 mg of HOBT (0.18 mmol), and 21.96 mg of DMAP (0.18 mmol) were dissolved in 5.0 mL DMF and stirred for 1.5 h with an ice bath to active the carboxyl group. Then 500 mg of mPEG-NH_2 (0.1 mmol) was dissolved in 15 mL DMF, slowly dripped into the above solution, and stirred for another 24 h at room temperature (RT). Then dialyzed with deionized water (MWCO = 3500 Da) for 24 h and vacuum dried to obtain white powder (379.5 mg, 69.0%). ^{1}H-NMR (500 MHz, $CDCl_3$) δ = 3.40 (s, 3 H), 3.45-3.85 (m, 466 H), 9.35 (s, 1 H), 9.48 (s, 1 H). *Mw* (MALDI-TOF): 5500 Da.

Synthesis of GPLGLAG-PEG_{5k}

149.6 mg of Fmoc-GPLGLAG (0.2 mmol), 114.1 mg of HATU (0.3 mmol), and 38.76 mg of DIEA were dissolved in 5.0 mL DMF and stirred for 1.5 h under an ice bath to active the carboxyl group. Then 500 mg of mPEG-NH_2 (0.1 mmol) was dissolved in 15 mL DMF, slowly dripped into the above solution, and stirred for 24 h at RT. Use a syringe to add 6 mL of TEA to the above reaction solution, continue stirring to remove Fmoc, and then use deionized water dialyzing (MWCO = 3500 Da) for 24 h. GPLGLAG-PEG was obtained as a white powder (386.6 mg, 68.3%) by lyophilization. ^{1}H-NMR (500 MHz, $CDCl_3$) δ = 0.88 (m,12 H), 3.45-3.85 (m, 460 H). *Mw* (MALDI-TOF): 5660 Da.

Synthesis of JQ1-COOH

JQ1 (300 mg, 0.66 mmol) was dissolved in 10.0 mL reaction solvent of TFA and DCM (the volume ratio is TFA: DCM=1:1) and stirred for 4 h at RT. The solution was vacuum condensed. After isolation by silica gel column chromatography, JQ1-COOH was obtained as a light yellow powder (316.7 mg, yield 93.2%). ^{1}H-NMR (400 MHz, $CDCl_3$) δ = 1.70 (s, 3H), 2.42 (s, 3H), 2.71 (s, 3H), 3.57-3.74 (m, 2H), 4.59-4.62 (t, 1H), 7.33-7.44 (d, 4H). ESI (electrospray ionization) MS: m/z = 401.05 $[M + H]^+$.

Synthesis of JQ1-thioketal (TK)

JQ1-COOH (250 mg, 0.625 mmol), EDCI (359 mg, 1.9 mmol), and DMAP (229 mg, 1.9 mmol) were dissolved in 2.0 mL of anhydrous DCM and stirred for 2 h at 0 °C to activate the carboxyl group. After that, 2,2-bis-(2-hydroxy-ethylsulfanyl)-propane (306 mg, 1.55 mmol) was rapidly added to the mixture solution and stirred for 24 h at RT. JQ1-TK was obtained after isolation by silica gel column chromatography as a light yellow powder (252 mg, yield 70.0 %). ^{1}H-NMR (400 MHz, MeOD) δ = 1. 62 (s, 6H), 1.70 (s, 3H), 2.49 (s, 3H), 2.67 (s, 3H), 2.71-2.79 (t, 2H), 2.98-3.01 (m,

2H), 3.53-3.62 (m, 2H), 3.68-3.74 (m, 2H), 4.36-4.51 (m, 2H), 4.61-4.65 (t, 1H), 7.33-7.43 (d, 4H), ESI-MS: m/z = 601.2 $[M + Na]^+$.

Synthesis of pPC-COOH

Succinic anhydride (120 mg, 1.2 mM), p-lysoPC (300 mg, 0.6 mM), and DMAP (150 mg, 1.2 mM) were dissolved in 2.0 mL anhydrous DCM and stirred for 48 h at 40 °C with N_2 protection. The solution was vacuum condensed. The raw product was purified by silica chromatography using an octadecyl (C18) column and methanol as the eluent to obtain pPC-COOH as a white powder (289.7 mg, yield 80.5%). ^{1}H-NMR (400 MHz, $CDCl_3$) δ = 0.86-0.90 (t, 3H), 1.25 (m, 24H), 1.56-1.60 (t, 2H), 2.27-2.31 (t, 2H), 2.42-2.47 (m, 2H), 2.64-2.67 (d, 2H), 3.27 (s, 9H), 3.76 (s, 2H), 3.96-4.16 (m, 4H), 4.25-4.28 (m, 2H), 5.26 (s, 1H). ESI-MS: m/z = 596.4 $[M + H]^+$.

Synthesis of PPa-GPLGLAG-PEG$_{5k}$

To synthesize PPa-GPLGLAG-PEG$_{5k}$, 15.3 mg of N-(3-(dimethylamino)-propyl)-N-ethylcarbodiimide hydrochloride (EDCI) (0.08 mmol), 10.81 mg of 1-hydroxybenzotriazole anhydrous (HOBT) (0.08 mmol), and 21.65 mg of PPa (0.04 mmol) were dissolved in 3 mL of anhydrous N,N-dimethylformamide (DMF) and stirred for 1.5 h at 0 °C to activate the carboxyl group of PPa under dark. Then 160 mg of the synthesized GPLGLAG-PEG$_{5k}$ (0.03 mmol) was dissolved in 6 mL DMF, slowly dripped into the above reaction solution, followed by stirring for 24 h at room temperature (RT), and then dialyzed with 95% ethanol for 24 h and deionized water (Molecular Weight Cut Off [MWCO] = 3500 Da) for 4 hours and vacuum dried to obtain a pale yellow solid powder (122.7 mg, 70.1%). ^{1}H-NMR (500 MHz, $CDCl_3$) δ = 0.89 (m, 12H), 3.40 (s, 3 H), 3.45-3.85 (m, 466 H), 9.35 (s, 1 H), 9.48 (s, 1 H). *Mw* (MALDI-TOF): 6200 Da.

Synthesis of ROS-sensitive JQ1-TK-pPC (JTP) and ROS-insensitive JQ1-pPC (JP)

To synthesize JTP, pPC-COOH (256 mg, 0.43 mmol), 4-dimethylaminopyridine (DMAP) (157 mg, 1.29 mmol), EDCI (247.1 mg, 1.29 mmol), N,N-Diisopropylethylamine (DIEA) (225 μL, 1.29 mmol) were dissolved in 4 mL of anhydrous dichloromethane (DCM) and stirred for 2 h at 0 °C to activate the carboxyl group. Then JQ1-TK (300 mg, 0.52 mmol) in anhydrous DCM was slowly added into the above mixture and stirred for 48 h at RT. The solution was vacuum condensed and precipitation by cold diethyl ether to obtain purified JTP (357.8 mg, yield 72.1%). ^{1}H-NMR (400 MHz, $CDCl_3$) δ = 0.86-0.89 (t, 3H), 1.25 (m, 24H), 1.70 (s, 2H), 2.41-2.48 (s, 3H), 3.68-3.74 (m, 2H), 4.22-4.41 (t, 4H), 4.61-4.65 (t, 1H), 7.33-7.43 (d, 4H). ESI (electrospray ionization) -MS: m/z = 1156.6 $[M + H]^+$.

To synthesize ROS-insensitive JP, JQ1-COOH (200 mg, 0.5 mmol), EDCI (288 mg, 1.5 mmol), DMAP (183 mg, 1.5 mmol), DIEA (194 mg, 1.5 mmol) was dissolved in 2.0 mL of anhydrous DCM and stirred for 2 h at 0 ℃ to activate the carboxyl group. After that, triethylene glycol (Tg, 225 mg, 1.5 mmol) was rapidly added to the mixture solution and stirred for 24 h at RT. JQ1-Tg was obtained after isolation by silica gel column chromatography (226 mg, yield 84.8 %). ^{1}H-NMR (400 MHz, MeOD) δ = 1.70 (s, 3H), 2.41 (s, 3H), 2.69 (s, 3H), 3.63-3.67 (m, 2H), 3.69-3.81 (m, 12H), 4.62-4.67 (t, 1H), 7.33-7.38 (m, 2H), 7.40-7.46 (m, 2H), API-ESI-LC-MS: m/z = 533.2 $[M + H]^+$. Then, pPC-COOH (50.0 mg, 0.084 mmol), 4-dimethylaminopyridine (DMAP) (37.8 mg, 0.252 mmol), EDCI (48.3 mg, 0.252 mmol), N,N-Diisopropylethylamine (DIEA) (32.5 mg, 0.252 mmol) was dissolved in 2 mL of anhydrous dichloromethane (DCM) and stirred for 2 h at 0 ℃ to activate the carboxyl group. Then JQ1-Tg (53.8 mg, 0.101 mmol) in anhydrous DCM was slowly added into the above mixture and stirred for 48 h at RT. The solution was vacuum condensed and was further purified via C18 silica gel column chromatography to obtain JP (22.4 mg, yield 24.0%). ESI-MS: m/z = 1110.5 $[M + H]^+$.

MMP-2 cleavage activity of PPa-GPLGLAG-PEG_{5k}

PPa-GPLGLAG-PEG_{5k} (1.0 mg/mL) and MMP-2 (40 μg/mL) in 20 mM Tris buffer (100 mM $CaCl_2$, 50 mM NaCl, 0.05% Brij35, pH 7.4) were mixed and incubated at 37 °C for 0, 5, 20, 40, 60 min. The assay was monitored using HPLC (Waters e2695, C18 column, 5 μm, 4.6×250 mm) with 60%-100% MeOH (0-4 min) and 100% MeOH (4-10 min) elution. And the molecular weights of the MMP-2 degradation solution were measured by MALDI-TOF MS.

ROS cleavage activity of JQ1-TK-pPC

JQ1-TK-pPC and PEG_{5k}-PPa were dissolved in 200 μL of methanol at an identical concentration of 1.0 mg/mL in 96-well plate, followed by 671 nm laser irradiation (150 mW/cm^2) for 0.5, 1, 2 or 5 min under dark. Afterward, LC-MS was used to examine the ROS responsiveness of pPC-TK-JQ1 (elution phase: methanol/water (9/1, v/v); Flow rate: 0.5 mL/min).

Fabrication and characterization of the Gd^{3+}-loaded ELNV nanovesicles

The filming-rehydration method was used to fabricate the prodrug nanovesicles[1]. To prepare Gd^{3+}-loaded ELNV or LNV nanovesicles, an aqueous solution of gadolinium chloride ($GdCl_3$) was added to the methanol solution of PPa-GPLGLAG-PEG_{5k} or PPa-PEG_{5k} at a Gd^{3+} to PPa molar ratio of 5:1. The solution was incubated overnight at room temperature to load Gd^{3+} into PPa. The methanol solution was dropwise added to 1.0 mL of DI water under sonication. The excess Gd^{3+} and organic solvent were removed by dialyzing against DI water. The Gd^{3+} concentration in the resultant nanovesicles was determined using inductively coupled plasma mass spectrometry (ICP-MS) measurements.

Fluorescence properties of ELNV and LNV nanovesicles

To evaluate aggregation-caused quenching of the fluorescence properties of ELNV, the prepared

ELNV nanovesicles with different PPa concentrations of 0, 3.125, 6.25, 12.5, 25, 50, and 100 μM were dissolved in 10% SDS and PBS. The fluorescence intensity of different solutions was detected by the microplate reader (Ex/Em = 410 nm/670 nm). the ELNV (with different PPa concentrations) in 10% SDS and PBS were further imaged with the IVIS-Imaging System (Ex/Em = 670 nm/690 nm).

Colloid stability of ELNV

To examine the serum stability of the prodrug vesicles, ELNV was incubated in PBS with 10% FBS at 37 °C, and the size of ELNV nanovesicles was monitored at 1, 2, 4, 6, 8, 12, and 24 h by DLS. To examine the long-term stability, ELNV in PBS with 10% FBS was placed at 4 °C and the size of ELNV nanovesicles was monitored at 1, 2, 3, 4, 5, and 6 d by DLS.

De-PEGylation of ELNV

The ELNV nanovesicles were incubated with different concentrations of MMP-2 (0 μg/mL, 25 μg/mL, 250 μg/mL, and 500 μg/mL) and the intensity size and PDI change of ELNV nanovesicles were detected at incubation times of 0, 10, 20, 30, 40, 50, 60, 100 and 150 min by DLS.

LY and PPa release from ELNV

The LY release profile of ELNV and LNV were evaluated by HPLC. Briefly, the ELNV and LNV prodrug nanovesicles were incubated with or without 40 μg/mL of MMP-2 at 37 °C. LY released from the nanovesicles was then monitored using HPLC with 70% acetonitrile elution at different times.

To investigate PPa release from the nanovesicles, 1 mL of ENV and ELNV suspension (100 μg/mL PPa) were added in dialysis tubes (MWCO = 3500 Da), respectively. 10 mL of 40 μg/mL MMP-2 was added outside the dialysis bags. Samples were taken out on different time points from 0 to 24 h.

PPa release from the nanovesicles into the solution was measured using a fluorescence spectrophotometer.

Cellular uptake

To investigate cellular uptake profile of the nanovesicles *in vitro*, 4T1 cells were incubated in 24-well tissue culture plate (6×10^4 cells/well) for 24 h. The cells were cultured with PBS, free PPa, ELNV, ELNV + MMP-2, LNV, and LNV + MMP-2 (the concentration of MMP-2 was 40 μg/mL) at the identical PPa concentration of 5.0 μM for 1, 2, 4, and 12 h, respectively. Then 4T1 cells were collected and examined by flow cytometry (BD FACS Calibur, BD, USA). To observe the cellular uptake of the nanovesicles more intuitively *in vitro*, 4T1 cells were incubated in 24-well plates (3×10^4 cells/well) for 24 h and then cultured with LNV, LNV + MMP-2, ELNV, and ELNV + MMP-2 (the nanovesicles were treated with 40 μg/mL MMP-2 for 60 min before adding to the cells) for 4 h. Afterwards, the cells were stained with DAPI and lysotracker green and examined by CLSM.

Photoactivity of the nanovesicles *in vitro* and *in vivo*

To investigate laser-induced ROS generation of ELNV *in vitro*, 5×10^4 4T1 cells in a 24-well plate were incubated with ELNV (PPa concentrations of 2.5 μM and 5 μM) for 4 h. After that, the cells were washed with PBS and added with 10 μM of 2',7'-dichlorofluorescein diacetate (DCFH-DA) for 30 min. The cells were irradiated with 671 nm laser for 1 min to generate ROS. The cells were collected and washed with PBS for FACS analysis. ELNV was pretreated with 40 μg/mL of MMP-2 for 24 h before adding to the cells.

To study the photoactivity *in vitro*, the cells were treated with PBS, free PPa, LNV, LNV + MMP-2, ELNV, ELNV + MMP-2, or ELNV + MMP-2 + Vc for 4 h (5 μM of PPa and 5 mM of Vc). After that, the cell samples were washed 3 times with 4 °C PBS. Then, 10 μM DCFH-DA was added to

each well and the DCFH-DA was incubated for a total of 30 min, followed by 671 nm laser irradiation for 1 min at 150 mW/cm^2 to generate ROS. The cells were collected and washed with PBS for FACS analysis. The MMP-2 group was pretreated with 40 μg/mL MMP-2 for 24 h before adding to the cells.

To analyze ROS generation by ELNV and ENV, 4T1 cells were incubated on a coverslip placed in 24 well plates at a density of 3×10^4 cells/well for 24 h and then cultured with LNV and ELNV (5 μM of PPa) for 4 h. LNV and ELNV were treated with 40 μg/mL MMP-2 for 60 min before adding to the cells. Then the cells were washed twice and stained with ready-used 4',6-diamidino-2-phenylindole dihydrochloride (DAPI) for 10 min. After that, the cells were washed with PBS 3 times. Then, 10 μM DCFH-DA was added and irradiated with 671 nm laser for 1 min at 150 mW/cm^2 to generate ROS. The cells were collected and washed with PBS for CLSM analysis.

To detect ROS generation *in vivo*, the subcutaneous 4T1 tumor models were established in BALB/c mice by subcutaneously inoculating 1×10^7 tumor cells/mouse. The tumor-bearing mice were randomly grouped (n = 3) when the tumor volume reached 150 mm^3. 4T1 tumor-bearing BALB/c mice were with i.v. injected with PBS, ENV or ELNV at the identical PPa dose of 5 mg/kg and LY dose of 20 mg/kg. After 8 h, 20 μL 1 mM DCFH-DA was injected intratumorally. The tumor tissues were irradiated with a 671 nm laser for 1 min (400 mW/cm^2) half an hour post-injection. After 12 h of the end of the irradiation, the mice were sacrificed and the tumor tissues were frozen sectioned and analyzed ROS generation by CLSM.

Immunofluorescence staining of macrophages

To further determine whether LY can polarize TAM into M1 phenotype, 4T1 tumor-bearing mice were intratumor injected with LY (0.75 or 1.5 mg/kg, n = 3) every other day. After one week, all mice were sacrificed and the tumors were sampled, immunofluorescence staining of the

tumor-infiltrating M1/M2 macrophages was subsequently performed on the tumor sections. We measured the percentage of M1 and M2 macrophages to the total macrophages in tumor tissues by ImageJ.

Supplementary References

1. Zhou F, et al. Overcoming immune resistance by sequential prodrug nanovesicles for promoting chemoimmunotherapy of cancer. *Nano Today* **36**, 101025 (2021).
2. Áron B, et al. TNMplot.com: A Web Tool for the Comparison of Gene Expression in Normal, Tumor and Metastatic Tissues. *Int. J. Mol. Sci.* **22**(5), 2622 (2021).
