# Supplementary material for: Nanovesicles loaded with a TGF-β receptor 1 inhibitor overcome immune resistance to potentiate cancer immunotherapy
Source: Nat Commun. 2023 Jun 16;14:3593. doi: 10.1038/s41467-023-39035-x (PMC10275881; doi:10.1038/s41467-023-39035-x)
Supplement: Supplementary file 2 — Reporting Summary [file 41467_2023_39035_MOESM2_ESM.pdf]

nature portfolio

Corresponding author(s): Prof. Haijun Yu

Last updated by author(s): May 03, 2023

Reporting Summary

Nature Portfolio wishes to improve the reproducibility of the work that we publish. This form provides structure for consistency and transparency in reporting. For further information on Nature Portfolio policies, see our Editorial Policies and the Editorial Policy Checklist.

Statistics

For all statistical analyses, confirm that the following items are present in the figure legend, table legend, main text, or Methods section.

n/a	Confirmed	
☐	☒	The exact sample size (*n*) for each experimental group/condition, given as a discrete number and unit of measurement
☐	☒	A statement on whether measurements were taken from distinct samples or whether the same sample was measured repeatedly
☐	☒	The statistical test(s) used AND whether they are one- or two-sided<br>*Only common tests should be described solely by name; describe more complex techniques in the Methods section.*
☒	☐	A description of all covariates tested
☒	☐	A description of any assumptions or corrections, such as tests of normality and adjustment for multiple comparisons
☐	☒	A full description of the statistical parameters including central tendency (e.g. means) or other basic estimates (e.g. regression coefficient) AND variation (e.g. standard deviation) or associated estimates of uncertainty (e.g. confidence intervals)
☐	☒	For null hypothesis testing, the test statistic (e.g. *F*, *t*, *r*) with confidence intervals, effect sizes, degrees of freedom and *P* value noted<br>*Give P values as exact values whenever suitable.*
☒	☐	For Bayesian analysis, information on the choice of priors and Markov chain Monte Carlo settings
☒	☐	For hierarchical and complex designs, identification of the appropriate level for tests and full reporting of outcomes
☒	☐	Estimates of effect sizes (e.g. Cohen's *d*, Pearson's *r*), indicating how they were calculated

Our web collection on statistics for biologists contains articles on many of the points above.

Software and code

Policy information about availability of computer code

Data collection	Malvern Zetasizer Nano ZS 7.11, Leica Application SuiteX 3.00, Image Lab 3.0, BD Accuri C6 software, BD FACSDiva software, PerkinElmer Living Image software
Data analysis	Statistical calculations were performed using Graphpad prism 8.0, Flow cytometry results were analyzed by FlowJoV10, Images were processed by ImageJ 1.52a and Zeiss ZEN 2.3 imaging software, NMR spectra were analyzed using Mestre Nova6.1.0-6224, Pharmacokinetic profiles was DAS (Data Analysis System) software (version 3.0).

For manuscripts utilizing custom algorithms or software that are central to the research but not yet described in published literature, software must be made available to editors and reviewers. We strongly encourage code deposition in a community repository (e.g. GitHub). See the Nature Portfolio guidelines for submitting code & software for further information.

Data

Policy information about availability of data

All manuscripts must include a data availability statement. This statement should provide the following information, where applicable:

- Accession codes, unique identifiers, or web links for publicly available datasets
- A description of any restrictions on data availability
- For clinical datasets or third party data, please ensure that the statement adheres to our policy

The data of Fig. 2a, c and supplementary Figures 1, 3-8 were downloaded directly from the online database TNMplot (https://tnmplot.com/analysis/). The data of Fig. 2b and supplementary Figure 16 were downloaded directly from the online database GEPIA (http://gepia.cancer-pku.cn/). The data of Fig. 2d was downloaded

directly from the online database TIMER (https://cistrome.shinyapps.io/timer/). The data of Fig. 2e and supplementary Figures 2, 9-10 were downloaded directly from the online database TIMER2.0 (http://timer.comp-genomics.org/). The authors declare that the data supporting the findings of this study are available within the article, source data, and its Supplementary Information. The source data underlying Figs. 2, 3, 4, 5, 6, 7, supplementary Figures 3-8, 11-13, 19-34, 41-51, supplementary Tables 2, 3, 4, 5 and western blot are provided with this paper. A reporting summary for this article is available as a Supplementary Information file. Source data are provided with this paper.

Human research participants

Policy information about studies involving human research participants and Sex and Gender in Research.

Reporting on sex and gender	*Use the terms sex (biological attribute) and gender (shaped by social and cultural circumstances) carefully in order to avoid confusing both terms. Indicate if findings apply to only one sex or gender; describe whether sex and gender were considered in study design whether sex and/or gender was determined based on self-reporting or assigned and methods used. Provide in the source data disaggregated sex and gender data where this information has been collected, and consent has been obtained for sharing of individual-level data; provide overall numbers in this Reporting Summary. Please state if this information has not been collected. Report sex- and gender-based analyses where performed, justify reasons for lack of sex- and gender-based analysis.*
Population characteristics	*Describe the covariate-relevant population characteristics of the human research participants (e.g. age, genotypic information, past and current diagnosis and treatment categories). If you filled out the behavioural & social sciences study design questions and have nothing to add here, write "See above."*
Recruitment	*Describe how participants were recruited. Outline any potential self-selection bias or other biases that may be present and how these are likely to impact results.*
Ethics oversight	*Identify the organization(s) that approved the study protocol.*

Note that full information on the approval of the study protocol must also be provided in the manuscript.

Field-specific reporting

Please select the one below that is the best fit for your research. If you are not sure, read the appropriate sections before making your selection.

☒ Life sciences ☐ Behavioural & social sciences ☐ Ecological, evolutionary & environmental sciences

For a reference copy of the document with all sections, see nature.com/documents/nr-reporting-summary-flat.pdf

Life sciences study design

All studies must disclose on these points even when the disclosure is negative.

Sample size	Sample size was chosen to ensure reproducibility of the experiments in accordance with the replacement, reduction and refinement principles of animal ethics regulation. Sample sizes employed in this study were referenced previously published studies (Nature Nanotechnology 2021,16(1): 103-113).
Data exclusions	No data was excluded in this study.
Replication	All experimental findings were reliably reproduced. At least three independent samples were performed for each experiment. All experiments were performed as technical or biological replications as appropriate for the experiment design. Details of experimental replicates are given in the figure legends.
Randomization	All samples were randomly allocated into experimental groups.
Blinding	No blinding was used throughout experiments. The investigators should keep careful track of protocols because that most of the experiments needed multiple treatments (including formulation, cells or mouse tumor treatment, sample collection, and so on). Hence, it would be difficult to blind the investigators to group allocation during data collection and analysis.

Reporting for specific materials, systems and methods

We require information from authors about some types of materials, experimental systems and methods used in many studies. Here, indicate whether each material, system or method listed is relevant to your study. If you are not sure if a list item applies to your research, read the appropriate section before selecting a response.

Materials & experimental systems

n/a	Involved in the study
☐	☒ Antibodies
☐	☒ Eukaryotic cell lines
☒	☐ Palaeontology and archaeology
☐	☒ Animals and other organisms
☒	☐ Clinical data
☒	☐ Dual use research of concern

Methods

n/a	Involved in the study
☒	☐ ChIP-seq
☐	☒ Flow cytometry
☒	☐ MRI-based neuroimaging

Antibodies

Antibodies used

Anti-alpha smooth muscle actin antibody [EPR5368] (ab124964), anti-CD3 antibody [SP162] (ab135372), anti-CD31 antibody [RM1006] (ab281583), anti-Calreticulin antibody [EPR3924] - ER Marker (ab92516), anti-HMGB1 antibody (ab18256) were purchased from Abcam. Recombinant anti-Smad2 (phospho S467) antibody [EPR23681-40] (ab280888), recombinant anti-Smad2 antibody [EP567Y] (ab33875), anti-Smad3 antibody (ab84177), recombinant anti-Smad3 (phospho S423 + S425) antibody [EP823Y] (ab52903) were obtained from Abcam. Recombinant anti-collagen I antibody [EPR24331-53] (ab270993), goat Anti-Rabbit IgG H&L (Alexa Fluor® 488) (ab150077) and goat anti-rabbit IgG H&L (Alexa Fluor® 555) (ab150078) were purchased from Abcam. Anti-fibronectin antibody (ab2413) was obtained from Abcam. Anti-fibroblast activation protein alpha antibody (NB110-85534) was obtained from Novus Biologicals. Anti-fibroblast activation protein, alpha antibody (ab218164) was obtained from Abcam. Anti-CD4 antibody [EPR19514] (ab183685), anti-CD8 alpha antibody [EPR21769] (ab217344) and Anti-PD-L1 antibody (ab233482) were obtained from Abcam. Anti-CD3-PerCP-Cy5.5 (551163), anti-CD45-APC (559864), anti-CD45-PerCP-Cy5.5 (550994), anti-CD8-PE (553033), anti-CD4-FITC (553046), anti-IFN-γ-FITC (554411), anti-Foxp3-PE (560414), anti-CD11c-FITC (557400), anti-CD80-PE (553769), anti-CD86-APC (558703), anti-CD274-APC (564715) and anti-CD274-PE (558091) were all purchased from BD Biosciences (Shanghai). Anti-CD45-FITC (BD, 553079), anti-CD3-PerCP-Cy5.5 (BioLegend, 100218), anti-CD4-APC/Cy7 (BD, 552051), anti-CD8-PE (BD, 553032), anti-IFN-γ-APC (BD, 554413), anti-CD25-APC (Invitrogen, 17-0257-42), anti-Foxp3-PE (Invitrogen, 72-5775-40), anti-CD45-PE (Multi Sciences, F2104502), anti-CD8-PE/Texas red (Abcam, ab25294), anti-CD44-APC (BD, 559250), anti-CD62L-FITC (BioLegend, 104405) and anti-CD326 (Ep-CAM)-PE antibody (BioLegend, 118205) were used . Anti-CD163 antibody [EPR19518] (ab182422), anti-CD68 antibody (Cell Signaling Technology, 97778), anti-CD86 antibody (Cell Signaling Technology, 19589) and anti-MMP2 antibody (ab86607) were purchased from Abcam.

Validation

All antibodies were commercially available and were validated by the supplier. All antibodies were used in the study according to the profile of manufacturers. All validation statements are available on the antibody websites, respectively.
1. https://www.abcam.cn/products/primary-antibodies/alpha-smooth-muscle-actin-antibody-epr5368-ab124964.html
2. https://www.abcam.cn/products/primary-antibodies/cd3-antibody-sp162-ab135372.html
3. https://www.abcam.cn/products/primary-antibodies/cd31-antibody-rm1006-ab281583.html
4. https://www.abcam.cn/products/primary-antibodies/smad2-phospho-s467-antibody-epr23681-40-ab280888.html
5. https://www.abcam.cn/products/primary-antibodies/smad2-antibody-ep567y-ab33875.html
6. https://www.abcam.cn/products/primary-antibodies/smad3-antibody-ab84177.html
7. https://www.abcam.cn/products/primary-antibodies/smad3-phospho-s423--s425-antibody-ep823y-ab52903.html
8. https://www.abcam.cn/products/primary-antibodies/collagen-i-antibody-epr24331-53-ab270993.html
9. https://www.abcam.cn/products/secondary-antibodies/goat-rabbit-igg-hl-alexa-fluor-488-ab150077.html
10. https://www.abcam.cn/products/secondary-antibodies/goat-rabbit-igg-hl-alexa-fluor-555-ab150078.html
11. https://www.abcam.cn/products/primary-antibodies/fibronectin-antibody-ab2413.html
12. https://www.novusbio.com/products/fibroblast-activation-protein-alpha-fap-antibody_nb110-85534
13. https://www.abcam.cn/products/primary-antibodies/fibroblast-activation-protein-alpha-antibody-ab218164.html
14. https://www.abcam.cn/products/primary-antibodies/cd4-antibody-epr19514-ab183685.html
15. https://www.abcam.cn/products/primary-antibodies/cd8-alpha-antibody-epr21769-ab217344.html
16. https://www.abcam.cn/products/primary-antibodies/pd-l1-antibody-ab233482.html
17. https://www.bdbiosciences.com/zh-cn/products/reagents/flow-cytometry-reagents/research-reagents/single-color-antibodies-ruo/percp-cy-5-5-hamster-anti-mouse-cd3e.551163
18. https://www.bdbiosciences.com/zh-cn/products/reagents/flow-cytometry-reagents/research-reagents/single-color-antibodies-ruo/apc-rat-anti-mouse-cd45.559864
19. https://www.bdbiosciences.com/zh-cn/products/reagents/flow-cytometry-reagents/research-reagents/single-color-antibodies-ruo/percp-cy-5-5-rat-anti-mouse-cd45.550994
20. https://www.bdbiosciences.com/zh-cn/products/reagents/flow-cytometry-reagents/research-reagents/single-color-antibodies-ruo/pe-rat-anti-mouse-cd8a.553033
21. https://www.bdbiosciences.com/zh-cn/products/reagents/flow-cytometry-reagents/research-reagents/single-color-antibodies-ruo/fitc-rat-anti-mouse-cd4.553046
22. https://www.bdbiosciences.com/zh-cn/products/reagents/flow-cytometry-reagents/research-reagents/single-color-antibodies-ruo/fitc-rat-anti-mouse-ifn.554411
23. https://www.bdbiosciences.com/zh-cn/products/reagents/flow-cytometry-reagents/research-reagents/single-color-antibodies-ruo/pe-rat-anti-mouse-foxp3.560414
24. https://www.bdbiosciences.com/zh-cn/products/reagents/flow-cytometry-reagents/research-reagents/single-color-antibodies-ruo/fitc-hamster-anti-mouse-cd11c.557400
25. https://www.bdbiosciences.com/zh-cn/products/reagents/flow-cytometry-reagents/research-reagents/single-color-antibodies-ruo/pe-hamster-anti-mouse-cd80.553769
26. https://www.bdbiosciences.com/zh-cn/products/reagents/flow-cytometry-reagents/research-reagents/single-color-antibodies-

ruo/apc-rat-anti-mouse-cd86.558703
27. https://www.bdbiosciences.com/zh-cn/products/reagents/flow-cytometry-reagents/research-reagents/single-color-antibodies-ruo/apc-rat-anti-mouse-cd274.564715
28. https://www.bdbiosciences.com/zh-cn/products/reagents/flow-cytometry-reagents/research-reagents/single-color-antibodies-ruo/pe-rat-anti-mouse-cd274.558091
29. https://www.bdbiosciences.com/zh-cn/products/reagents/flow-cytometry-reagents/research-reagents/single-color-antibodies-ruo/fitc-rat-anti-mouse-cd45.553079
30. https://www.biolegend.com/en-us/products/percp-cyanine5-5-anti-mouse-cd3-antibody-5596
31. https://www.bdbiosciences.com/zh-cn/products/reagents/flow-cytometry-reagents/research-reagents/single-color-antibodies-ruo/apc-cy-7-rat-anti-mouse-cd4.552051
32. https://www.bdbiosciences.com/zh-cn/products/reagents/flow-cytometry-reagents/research-reagents/single-color-antibodies-ruo/pe-rat-anti-mouse-cd8a.553032
33. https://www.bdbiosciences.com/zh-cn/products/reagents/flow-cytometry-reagents/research-reagents/single-color-antibodies-ruo/apc-rat-anti-mouse-ifn.554413
34. https://www.thermofisher.cn/cn/zh/antibody/product/CD25-Antibody-clone-CD25-4E3-Monoclonal/17-0257-42
35. https://www.thermofisher.cn/order/catalog/product/72-5775-40?SID=srch-srp-72-5775-40
36. https://www.liankebio.com/product/anti-mouse-cd45-pe-clone-30-f11-15416.html
37. https://www.abcam.cn/products/primary-antibodies/petexas-red-cd8-alpha-antibody-53-67-ab25294.html
38. https://www.bdbiosciences.com/zh-cn/products/reagents/flow-cytometry-reagents/research-reagents/single-color-antibodies-ruo/apc-rat-anti-mouse-cd44.559250
39. https://www.biolegend.com/en-us/products/fitc-anti-mouse-cd62l-antibody-384
40. https://www.biolegend.com/en-us/products/pe-anti-mouse-cd326-ep-cam-antibody-4726
41. https://www.abcam.cn/products/primary-antibodies/cd163-antibody-epr19518-ab182422.html
42. https://www.cellsignal.cn/products/primary-antibodies/cd68-e3o7v-rabbit-mab/97778?site-search-type=Products&N=4294956287&Ntt=97778&fromPage=plp&_requestid=275142
43.https://www.cellsignal.cn/products/primary-antibodies/cd86-e5w6h-rabbit-mab/19589?site-search-type=Products&N=4294956287&Ntt=19589&fromPage=plp&_requestid=275384
44. https://www.abcam.cn/products/primary-antibodies/mmp2-antibody-6e3f8-ab86607.html
45. https://www.abcam.cn/products/primary-antibodies/hmgb1-antibody-ab18256.html
46. https://www.abcam.cn/products/primary-antibodies/calreticulin-antibody-epr3924-er-marker-ab92516.html

Eukaryotic cell lines

Policy information about cell lines and Sex and Gender in Research

Cell line source(s)	Panc02 murine pancreatic tumor cells, 4T1 murine breast cancer cells, B16-F10 murine melanoma tumor cells, and MC38 murine colorectal tumor cells were all obtained from the cell bank of the Chinese Academy of Sciences (Shanghai, China). NIH3T3 cells were purchased from Shanghai Bogoo Biotechnology. Co., Ltd. KPC pancreatic tumor cells were kindly provided by Dr. Y. Huang from SIMM of CAS, China. KPC cells were obtained from the primary KPC tumors in Pdx-Cre; KrasG12D/+; Trp53R172H/+ mice.
Authentication	These cell lines were authenticated by the supplier using STR analysis.
Mycoplasma contamination	All cell lines were tested negative for mycoplasma contamination. Contamination was detected by the supplier using Hoechst DNA stain method, agar culture method, and PCR-based assay.
Commonly misidentified lines (See ICLAC register)	These all cell lines that we used were not listed in commonly misidentified lines in ICLAC register.

Animals and other research organisms

Policy information about studies involving animals; ARRIVE guidelines recommended for reporting animal research, and Sex and Gender in Research

Laboratory animals	BALB/c mice (female, 6~ 8 weeks, 18 ~ 20 g), C57BL/6 (female, 6 ~ 8 weeks, 18~20 g), and BALB/c nude mice (female, 6 ~ 8 weeks, 18 ~ 20 g). Animals were housed under SPF conditions in groups of 4–5 mice per cage, and maintained at a temperature of ~25 °C in a humidity-controlled environment with a 12 h light/dark cycle, with free access to standard food and water.
Wild animals	No wild animals were used in this study.
Reporting on sex	We did not consider the influence of sex in the study design. Female mice were used for all the animal assay as reported in the literature studies.
Field-collected samples	No field-collected samples were used in this study.
Ethics oversight	All animal procedures were carried out under the guidelines approved by the Institutional Animal Care and Use Committee (IACUC) of Shanghai Institute of Material Medica, Chinese Academy of Sciences.

Note that full information on the approval of the study protocol must also be provided in the manuscript.

Flow Cytometry

Plots

Confirm that:

- [x] The axis labels state the marker and fluorochrome used (e.g. CD4-FITC).
- [x] The axis scales are clearly visible. Include numbers along axes only for bottom left plot of group (a 'group' is an analysis of identical markers).
- [x] All plots are contour plots with outliers or pseudocolor plots.
- [x] A numerical value for number of cells or percentage (with statistics) is provided.

Methodology

Sample preparation	To research the cellular uptake of the nanovesicles in vitro, 4T1 cells were incubated in 24-well plates (6×104 cells/well) for 24 h. The cells were cultured with PBS, free PPa, ELNV, ELNV + MMP-2, LNV, and LNV + MMP-2 (the concentration of MMP-2 was 40 µg/mL) at the identical PPa concentration of 5.0 µM for 1, 2, 4, and 12 h, respectively. The cells were washed three times with PBS, detached by trypsin-EDTA and finally collected by centrifugation at 1000 rpm for 3 min. The bottom cells were washed three times with PBS and then suspended cells were analyzed by flow cytometry.<br>Immune cells in isolated tumor tissues, lymph nodes or spleen were further analyzed to evaluate the immune activation ability of nanovesicles in vivo. Single-cell suspensions were prepared using a GentleMACs dissociator (Milltenyi). Then, the cell suspensions (100 µL) were stained with antibodies for 30 min at 4 °C. The cells were washed with cold PBS three times and analyzed by flow cytometry.
Instrument	BD Accuri C6 flow cytometer for analysis; BD FACSAria II flow cytometer for cell sorting
Software	BD Accuri C6 software and BD FACSDiva software were used to collect the data. FlowJoV10 software was used to analyse the data.
Cell population abundance	At least 10000 cells were used for flow cytometric analysis, while at least 1000000 cells were used for flow cytometric sorting.
Gating strategy	Cells were gated on FSC/SSC in general.

- [x] Tick this box to confirm that a figure exemplifying the gating strategy is provided in the Supplementary Information.
